# Society for Cardiovascular Magnetic Resonance (SCMR) expert consensus for CMR imaging endpoints in clinical research: part I - analytical validation and clinical qualification

**DOI:** 10.1186/s12968-018-0484-5

**Published:** 2018-09-20

**Authors:** Valentina O. Puntmann, Silvia Valbuena, Rocio Hinojar, Steffen E. Petersen, John P. Greenwood, Christopher M. Kramer, Raymond Y. Kwong, Gerry P. McCann, Colin Berry, Eike Nagel, on behalf of SCMR Clinical Trial Writing Group, Colin Berry, David Bluemke, Jens Bremerich, Rene Botnar, Chiara Bucciarelli-Ducci, Robin P. Choudhury, Marc Dweck, Ingo Eitel, Vic Ferrari, Matthias Friedrich, John Greenwood, Rocio Hinojar, Greg Hundley, Christopher M. Kramer, Raymond Y. Kwong, Massimo Lombardi, Teresa Lopez Fernandez, Thomas Marwick, Eike Nagel, Jagat Narula, Stefan Neubauer, Amit Patel, Dudley Pennell, Steffen E. Petersen, Sven Plein, Sanjay Prasad, Valentina O. Puntmann, Frank Rademakers, Subha Raman, Hajime Sakuma, Javier Sanz, Jeannette Schulz-Menger, Orlando Simonetti, Andrew Swift, Andrew J. Taylor, T. Teixeira, Holger Thiele, Martin Ugander, Silvia Valbuena, Jos J. Westenberg, Alistair A. Young

**Affiliations:** 1 Institute of Experimental and Translational Cardiovascular Imaging, Goethe University Hospital Frankfurt, Frankfurt, Germany; 2 Department of Cardiology, Goethe University Hospital Frankfurt, Frankfurt, Germany; 3 Department of Cardiology, University Hospital La Paz, Madrid, Germany; 4 Department of Cardiology, University Hospital Ramón y Cajal, Madrid, Spain; 5 William Harvey Research Institute, Queen Mary University of London, Barts and the London NIHR Biomedical Research Centre at Barts, London, UK; 6 Leeds Institute of Cardiovascular and Metabolic Medicine, University of Leeds, Leeds, UK; 7 Department of Medicine (Cardiology) and Radiology, Cardiovascular Imaging Center, University of Virginia Health System, Charlottesville, Virginia USA; 8 Cardiovascular Division, Department of Medicine, Brigham and Womens’ Hospital, Boston, Massachusetts USA; 9 Department of Cardiovascular Sciences, University of Leicester, Leicester, UK; 10 the NIHR Leicester Cardiovascular Biomedical Centre, University Hospitals of Leicester NHS Trust, Glenfield Hospital, Leicester, UK; 11 British Heart Foundation Glasgow Cardiovascular Research Centre, University of Glasgow, Glasgow, UK; 12 West of Scotland Heart and Lung Centre, Golden Jubilee National Hospital, Clydebank, UK

**Keywords:** Cardiac magnetic resonance, Imaging, Biomarker, Position paper, SCMR

## Abstract

Cardiovascular disease remains a leading cause of morbidity and mortality globally. Changing natural history of the disease due to improved care of acute conditions and ageing population necessitates new strategies to tackle conditions which have more chronic and indolent course. These include an increased deployment of safe screening methods, life-long surveillance, and monitoring of both disease activity and tailored-treatment, by way of increasingly personalized medical care. Cardiovascular magnetic resonance (CMR) is a non-invasive, ionising radiation-free method, which can support a significant number of clinically relevant measurements and offers new opportunities to advance the state of art of diagnosis, prognosis and treatment. The objective of the SCMR Clinical Trial Taskforce was to summarizes the evidence to emphasize where currently CMR-guided clinical care can indeed translate into meaningful use and efficient deployment of resources results in meaningful and efficient use. The objective of the present initiative was to provide an appraisal of evidence on *analytical validation*, including the accuracy and precision, and *clinical qualification* of parameters in disease context, clarifying the strengths and weaknesses of the state of art, as well as the gaps in the current evidence This paper is complementary to the existing position papers on standardized acquisition and post-processing ensuring robustness and transferability for widespread use. Themed imaging-endpoint guidance on trial design to support drug-discovery or change in clinical practice (part II), will be presented in a follow-up paper in due course. As CMR continues to undergo rapid development, regular updates of the present recommendations are foreseen.

**Electronic supplementary material:**

The online version of this article (10.1186/s12968-018-0484-5) contains supplementary material, which is available to authorized users.

## Outline

1. Rationale
2. Imaging measures as biomarkers and surrogate endpoints – review of the definitions and guiding principles
3. Executive Statements of the SCMR CT Committee Writing Group
4. Quantification of ventricular volumes and mass
  i. Global volumes, mass, thickness and function
  ii. Regional wall motion, deformation and dyssynchrony
  iii. Diastolic function
5. Tissue characterisation - visualisation
  i. LGE (myocardial scar, infarct size, enhancement score)
  ii. T2 imaging (myocardial inflammation and area of risk)
6. Quantitative tissue characterization (T1, T2, T2* mapping)
  i. T1 mapping
  ii. T2 mapping
  iii. T2* mapping
7. Myocardial perfusion imaging
8. Vascular endpoints

## 1. Rationale

**Fig. 1:**
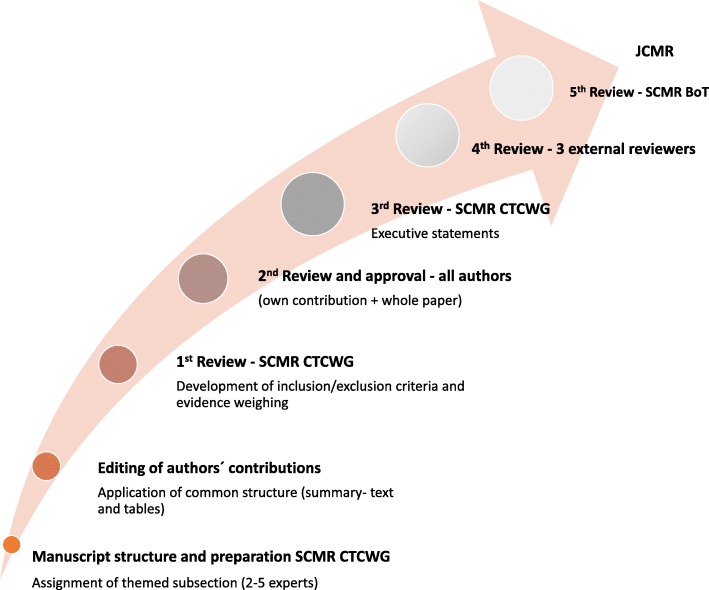
The illustration of reviewing steps involved in generation of this position paper. The manuscript structure, preparation and evidence appraisal procedures were based on a prior agreement within the SCMR Clinical Trial (CT) Writing Group (WG), as well as general guidance of the SCMR on Expert Consensus publications. Please see the Rationale for details

Cardiovascular disease (CVD) remains the greatest cause of morbidity and mortality globally. The changing natural history of CVD due to improved care of acute conditions and ageing population necessitates new strategies to tackle conditions with a more chronic and indolent course. These include an increased deployment of safe screening methods, life-long surveillance, and monitoring of both disease activity and tailored-treatment, by way of increasingly personalised medical care. Cardiovascular magnetic resonance (CMR), is a non-invasive, radiation-free method, which can support a significant number of clinically relevant measurements, offers many new opportunities to advance the state of art of diagnosis, prognosis and treatment of patients with CVD. Several key CMR measurements are highly accurate and reproducible, providing gold-standard measures in cardiovascular imaging. Published agreements on standardized acquisition and post-processing ensure robustness and transferability for widespread use [1, 2]. With the growing evidence on diagnostic and prognostic role, CMR measurements may be well-suited as imaging biomarkers for assessment of novel clinical management pathways and therapies. Yet, despite the enthusiasm, the body of evidence, and the overt potential to improve patients care, the impact of CMR towards clinical cardiology practice remains limited, by way of access to the technology (scanner, scan-time), operational imaging skill and allocation of the healthcare resources. Hence, the objective of this taskforce is to emphasize the evidence where CMR-guided clinical care indeed means that deployment of resources results in meaningful and efficient use, by providing an appraisal of evidence on *analytical validation*, including the accuracy and precision, and *qualification* of parameters in disease context (part I). The manuscript structure, preparation and evidence appraisal procedures were based on a prior agreement within the SCMR CT Writing Group (WG) (Fig. 1**-** Flowchart), as well as general guidance of the SCMR on Expert Consensus publications. This included the assignment of themed subsection to a minimum of 2 and a maximum of 5 authors with background of contribution to the field, which are included in the authors’ list (Table 1). The resulting material was subsequently reviewed and edited to adopt a common reporting format of a summary-text and evidence-rich tables. As per SCMR CT WG consensus, the studies were included, if providing a robust independent (non-CMR) comparator (validation) or including > 50 subjects (normal values) or > 25 in patient group (proof of concept), and > 100 for outcome study. Smaller studies were included if no other evidence was available and with consensus of the writing group. We strived to set out qualified recommendations for appropriate surrogate use of imaging measures biomarkers using consensus statements produced by the SCMR CT WG upon the presentation of summarised data. The weighing of evidence was based on consensus criteria of the SCMR CT WG, and assigned as promising, if multiple (3 or more) publications from independent groups existed, and favourable, if also cited by the practice guidelines. The final steps included a review and approval by all co-authors, followed by 3 independent external reviewers, commissioned by the SCMR Board of Trustees, in line with the societal rules on consensus statements. This report clarifies the strengths and weaknesses of the state of art, as well as the gaps in the current evidence (Table 2). The SCMR CT WG statements are based on the available evidence up to and including April 2017. The target audience includes clinical investigators considering the application of CMR-imaging endpoints in clinical studies and trials involving human subjects. Themed imaging-endpoint guidance on trial design to support drug-discovery or change in clinical practice (part II), will be presented in a follow-up paper in due course. As CMR continues to undergo rapid development, regular updates of the present recommendations are foreseen.

**Table 1:** Characteristics of contributing authors

Contributing authors’ characteristics	Count (%); Median(IQR)
MR Vendors	
Siemens	26 (53)
Philips	17(35)
General Electrics	6(12)
Field Strength	
1.5 Tesla	37(76)
3.0 Tesla	17(35)
Both	15(31)
Specialty	
Cardiology	25(51)
Radiology	18(38)
Other	5(10)
Number of previously co-authored Societal consensus papers (any)	3(1–4.5)
Number of previously co-authored SCMR Consensus papers	2(1–3)
^a^Number of previously co-authored papers in the CMR field	86(57–141)
^b^Number of previously co-authored papers in the author’s themed field	44(23–68)

Search criteria (Pubmed): ^a^Surname, First initial + cardiac + magnetic; ^b^Surname, First initial + magnetic+ theme

**Table 2:** Summary table for Executive statements for CMR endpoints

		Number of studies	Total number of subjects
Ventricular volumes and function			
CMR is the reference standard for quantification of LV and RV volumes, function and mass. CMR should be considered as the first line technique in clinical trials requiring one of these parameters for in- or exclusion or as an endpoint. The evidence for the use of quantification of cardiac function and volumes is favourable.			
*Analytical validation*	Excellent validation of LV mass and volumes	7	121
*Precision*	Large body of evidence on interstudy, inter- and intraobserver reproducibility	2*	32
*Normal values*	Available for various field strengths, imaging sequences, post-processing approaches, age-, sex- and ethnicity groups	9	6895
*Qualification/Utilisation*	The original evidence base by transthoracic echocardiography has been revalidated and expanded upon by CMR	7	14,711
Regional wall motion, deformation and dyssynchrony			
CMR-based strain-imaging techniques seem similarly suited as echocardiographic techniques for assessing longitudinal motion and strain. The evidence for the use of CMR-based strain imaging techniques is promising.			
*Analytical validation*	CMR tagging techniques have been well validated. Other MR based strain imaging techniques have been either directly compared with tagging or indirectly against a technique originally compared to tagging.	11	600
*Precision*	Limited data on inter-study reproducibility	9	168
*Normal values*	Normal values are available, but show considerable regional variation as well as variation between different studies	11	3191
*Qualification/Utilisation*	Outcome data suggest utility in addition to standard measures of care in clinical management.	5	2462
Diastolic function			
CMR may have advantages over other techniques by direct assessment of myocardial tissue. The evidence for the use of CMR-based assessment of diastolic function is promising.			
*Analytical validation*	Reasonably well validated versus PV loops and echocardiography for diastolic filling, atrial volumes and function and transmitral and pulmonary venous flow	4	212
Late gadolinium enhancement			
CMR based LGE should be used as the first line technique in clinical trials requiring the assessment of regional scar or fibrosis for inclusion or exclusion or as an endpoint. CMR should also be employed for optimal risk-classification of trial subjects with ischemic or non-ischemic cardiomyopathies. The evidence for the use of LGE imaging for visual detection of regional myocardial fibrosis and quantification of ischaemic scar is favourable. The quantification of non-ischaemic scar remains promising.			
*Analytical validation*	Extensively validated as a marker of irreversible damage post myocardial infarction in animals as well as versus biopsies, in explanted hearts and versus other imaging techniques	8	406
*Precision*	Strong data on inter-study reproducibility	7	200
*Qualification/Utilisation*	Strong parameter to predict outcome, superior to volumes and function.	37	12,562
T2-weighted imaging			
Due to the availability of many different sequences no generally accepted standard has been defined. For clinical trials, it is important to use a validated and standardized approach amongst different centres and vendors and use normal values and effect sizes specifically for these sequences. The evidence for the use of T2W imaging of AAR is promising.			
*Analytical validation*	Well validated in animals, phantoms and humans	15	817
*Precision*	Scarce data on inter-study reproducibility in acute myocardial infarction. Lack of reproducibility data and outcome studies for T2W-oedema imaging in inflammatory cardiac conditions	4	234
*Qualification/Utilisation*	Small number of outcome studies using AAR	17	1509
T1 mapping			
Due to the availability of many different sequences, no generally accepted standard has been defined. To employ T1 mapping in clinical trials, the use of validated (well understood sequence) and standardized approach amongst different centres and vendors is mandatory, due to the different normal values and effect sizes between various sequences. CMR T1-mapping may be considered as a standard for adequate risk-assessment of patients with non-ischemic dilated cardiomyopathy in clinical trials. The evidence for the use of T1 mapping is promising.			
*Analytical validation*	Well validated in phantoms, animal models, human biopsies and explanted hearts.	15	267
*Precision*	Evidence on interstudy, inter- and intraobserver variability	10	270
*Normal values*	Sequence-specific normal values available	4	1735
*Qualification/Utilisation*	Strong predictors of outcome in non-ischaemic dilated cardiomyopathies, superior to volumes, function and LGE.	37	6153
T2 mapping			
Due to the availability of many different sequences no generally accepted standard has been defined. The use a validated and standardized approaches amongst different centres and vendors is mandatory for the use in clinical trials, due to the different normal values and effect sizes specifically for these sequences. The timing of imaging after an acute event must be highly standardized. The evidence for the use of T2 mapping is promising.			
*Analytical validation*	Well validated against phantoms, animal models, human biopsies and other imaging biomarkers	11	340
*Precision*	Evidence on interstudy, inter- and intraobserver variability	1*	73
*Normal values*	Sequence-specific normal values available	3	205
*Qualification/Utilisation*	Useful in detecting myocardial oedema and inflammation	12	680
T2* mapping			
T2* can be regarded as the clinical reference standard in thalassemia and provide superior outcome data if used for therapy guidance. T2* measurements during or shortly after an acute coronary or vascular event provides important prognostic information in terms of short-term LV remodelling. The evidence for the use of T2* mapping is favourable.			
*Analytical validation*	Excellently validated and standardized in iron-overload	17	1728
*Precision*	Evidence on interscanner, intercenter, interstudy, inter- and intraobserver variability	4*	59
*Normal values*	Normal values and established clinically relevant cut-offs available	5	100
*Qualification/Utilisation*	Outcome data in thalassaemia major. Prognostic information after a coronary event	19	1778
Stress myocardial perfusion			
Perfusion imaging should be considered as a first line technique for assessing the presence, extent and localization of inducible ischemia. Its use for full quantification requires locally validated and standardized sequences with specific normal values. The evidence for the use of myocardial perfusion imaging for visual detection of ischaemia is favourable. The quantification remains promising.			
*Analytical validation*	Well-validated against animal models and alternative techniques	63	10,916
*Precision*	Limited evidence on interstudy, inter- and intraobserver reproducibility due to need of stress and contrast injection	5*	73
*Normal values*	Limited data on normal values due to lack of standardization of image acquisition and post-processing	3	42
*Qualification/Utilisation*	Large body of evidence showing significant predictive association for the presence/severity of myocardial ischemia with outcome	14	26,494
Vascular			
CMR vascular imaging is well suited to assess vascular anatomy and function. Aortic and carotid vessel wall imaging, are robust markers of atherosclerotic burden in these vessels and can be used in clinical trials.			
*Analytical validation*	Validation of PWV against alternative techniques and T2 mapping against histology	7	237
*Precision*	Limited evidence on interstudy reproducibility of anatomical and tissue measurements. Excellent evidence for PWV	6	95
*Normal values*	Available for different anatomical and functional measurements	7	4112
*Qualification/Utilisation*	Aortic wall imaging and PWV serve robust biomarkers of cardiovascular risk	2	5797

*LV* left ventricle, *RV* right ventricle, *PV* pressure-volume, *LGE* ate gadolinium enhancement, *AAR* area at risk, *PWV* pulse wave velocity. *Only studies reporting interstudy variability are included

## 2. Imaging parameters as biomarkers and endpoints

A biomarker is a characteristic that can be objectively measured and evaluated as an indicator of normal biological processes, pathogenic processes or pharmacological response to a therapeutic intervention [3]. They can serve as indicators of a disease presence or activity and reflect the rate of disease progression and response to treatment. Reliable biomarker’s characteristics include an accurate measurement, which is reproducible across multiple laboratories, and in a clinical setting, an adequate sensitivity and specificity for disease detection, severity and prognostic outcome. The biomarker evaluation framework foresees the following steps of biomarker characterisation: analytical validation, clinical qualification and utilization, and subsequently, a constant re-evaluation of the preceding steps [3]. It is also a sequence of interdependent steps, which continually inform each other. This process clarifies the biomarker’s application within a defined disease context and possible roles, from exploratory use to surrogate endpoint. ***Analytical validation*** involves assessment of assays or techniques supporting the acquisition of measurements, as well as establishing the range of conditions, under which the measurement will give reproducible and accurate data. Important performance metrics include precision, accuracy, lab-to-lab reproducibility, limits of detection and signal-to-noise, as well as determination of the reference values. ***Qualification*** refers to summation of the available evidence about the biomarker-disease-relationship, including its relevance, diagnostic and prognostic value. Also, causal relationships to disease pathogenesis are considered, including the effects of therapeutic intervention on the imaging marker and to the clinical endpoints of interest. **(4)** ***Utilization*** is a contextual analysis of the above evidence with regards to the definition of the context for the biomarker’ proposed clinical use. In addition, factors such as prevalence, heterogeneity, morbidity and mortality of the disease, as well as the risks and benefits of an intervention are considered.

## 3. Executive statements - SCMR Clinical Trial Writing Group

CMR uniquely provides quantitative information on cardiac function (LV, RV and valves) and myocardial tissue characteristics that are diagnostic of acute and chronic disease. CMR involves contrast- and non-contrast media imaging techniques. CMR does not involve ionising radiation and can be safely repeated.

### Ventricular volumes and function

The ability of CMR to assess left ventricular (LV) and right ventricular (RV) volumes and function accurately and precisely has been demonstrated in excellent validation studies with a large body of evidence on inter-study reproducibility. Standardised approach to quantification is available. There are extensive sets of normal values for gradient echo (GRE) sequences, whereas smaller sets support the modern acquisition techniques (balanced steady state free precession (bSSFP)). Thus, the appropriate reference ranges need to be selected for the technique used. The accuracy and reproducibility of novel post-processing algorithms based on signal intensity thresholding remains unknown. CMR is rightly regarded as the reference standard for the assessment of left and right ventricular volumes and left ventricular mass. CMR should be considered as the first line technique in clinical trials requiring one of these parameters for in- or exclusion or as an endpoint. The evidence for the use of quantification of cardiac function and volumes is favourable.

### i. Regional wall motion, deformation and dyssynchrony

CMR tagging techniques have been well validated. Other CMR based strain imaging techniques have been either directly compared with tagging or indirectly against a technique originally compared to tagging. Accuracy, precision and normal values are still to be further improved especially for radial strain and strain velocities. While normal values are available they show considerable regional variation as well as variation between different studies. There is very limited data on inter-study reproducibility. CMR-based strain-imaging techniques seem similarly suited as echocardiographic techniques for assessing longitudinal motion and strain. The evidence for the use of CMR-based strain imaging techniques is promising.

### Diastolic function

CMR has been reasonably well validated versus pressure volume (PV) loops and echocardiography for diastolic filling, atrial volumes and function and transmitral and pulmonary venous flow. More work is required to fully establish its role based on classic LV inflow (filling) parameters (e.g. E/e’). Although CMR may have advantages over other techniques by direct assessment of myocardial tissue, more evidence to support the use of CMR-based assessment of diastolic function is needed.

### Late gadolinium enhancement (LGE)

Late gadolinium enhancement (LGE) has been extensively validated as a marker of irreversible damage post myocardial infarction in animals as well as versus biopsies and in explanted hearts, demonstrating excellent accuracy and precision superior to alternative techniques. There are strong data on inter-study reproducibility. LGE is a strong parameter to predict outcome and has been shown to be superior to volumes and function. CMR based LGE should be used as the first line technique in clinical trials requiring the assessment of regional scar or fibrosis for inclusion or exclusion or as an endpoint. CMR should also be employed for optimal risk-classification of trial subjects with ischemic or non-ischemic cardiomyopathies. The evidence for the use of LGE imaging for visual detection of regional myocardial fibrosis and quantification of ischaemic scar is favourable. The quantification of non-ischaemic scar remains promising.

### T2-weighted imaging

T2-weighted imaging (T2W) has been well validated in animals, phantoms and humans and demonstrates an excellent ability to visualize areas of significantly increased tissue water or myocardial haemorrhage. There are scarce data on inter-study reproducibility in acute myocardial infarction. There is a small number of outcome studies in area at risk (AAR), including an application in a clinical trial. There is lack of reproducibility data and outcome studies for T2W-oedema imaging (or Lake-Louise criteria) in inflammatory cardiac conditions. Due to the availability of many different sequences no generally accepted standard has been defined. For clinical trials, it is important to use a validated and standardized approach amongst different centres and vendors and use normal values and effect sizes specifically for these sequences. Timing of imaging after an acute event must be highly standardized. Contrast-enhanced cine bSSFP imaging is emerging as possible time-efficient option for imaging the AAR. The evidence for the use of T2W imaging of AAR is promising.

### T1-mapping

Myocardial T1-mapping has been well validated in phantoms, animal models, and human biopsies and explanted hearts. In model diseases, the various acquisition techniques demonstrate the ability to relate to diffuse fibrosis, increased extracellular space and oedema, in a quantifiable fashion. T1-mapping indices have been shown to be strong predictors of outcome in non-ischaemic dilated cardiomyopathies, superior to volumes, function and LGE. Due to the availability of many different sequences, no generally accepted standard has been defined. To employ T1 mapping in clinical trials, the use of validated (well understood sequence) and standardized approach amongst different centres and vendors is mandatory, due to the different normal values and effect sizes between various sequences. CMR T1-mapping may be considered as a standard for adequate risk-assessment of patients with non-ischemic dilated cardiomyopathy in clinical trials. The evidence for the use of T1 mapping is promising.

### T2-mapping

Myocardial T2-mapping has been well-validated in phantoms, animal models, and human biopsies. The various techniques demonstrate an excellent ability to relate to myocardial water content/oedema, in a quantifiable fashion. Due to the availability of many different sequences no generally accepted standard has been defined. Similar to T1 mapping above, the use a validated and standardized approaches amongst different centres and vendors is mandatory for the use in clinical trials, due to the different normal values and effect sizes specifically for these sequences. The timing of imaging after an acute event must be highly standardized. The evidence for the use of T2 mapping is promising.

### T2* -mapping

Myocardial T2* mapping measurements in thalassemia have been excellently validated and standardized for 1.5 T and provide superior outcome data if used for therapy guidance. As such T2* can be regarded as the clinical reference standard in thalassemia. T2* measurements during or shortly after an acute coronary or vascular event provides important prognostic information in terms of short-term LV remodelling. The evidence for the use of T2* mapping is favourable.

### Perfusion imaging

CMR perfusion imaging has been well-validated against animal models, alternative techniques as well as related to outcomes. In various meta-analyses, CMR perfusion imaging has been confirmed as the most accurate technique for non-invasive assessment of myocardial ischemia. However, due to the availability of many different sequences and post-processing parameters, no generally accepted standard for (semi-) quantification has been defined. While there is good correlation of quantification techniques with microspheres and positron emission tomography (PET), normal values are variable and show high inter-study variability. Perfusion imaging should be considered as a first line technique for assessing the presence, extent and localization of inducible ischemia, its use for full quantification requires locally validated and standardized sequences with specific normal values. Trials assessing reduction in ischaemic burden following intervention are currently lacking. The evidence for the use of myocardial perfusion imaging for visual detection of ischaemia is favourable. Quantitative perfusion imaging is increasingly becoming available. At this stage the various approaches require more validation, especially as large outcome studies have been performed with visual analysis.

### Vascular imaging

Vascular imaging provides robust quantifiable data on vessel diameters, vessel wall thickness and vessel distensibility. Vascular stiffness measurements aside, there are limited data on truth validation or interstudy reproducibility for some vascular areas. Limited data supports CMR being non-inferior to computed tomography (CT) for aortic visualization and dimensions. Large databases of normal data are available. CMR vascular imaging is well suited to assess vascular anatomy and function. Aortic and carotid vessel wall imaging, are robust markers of atherosclerotic burden in these vessels and can be used in clinical trials. Coronary vessel wall imaging and tissue characterization is a promising research tool but require further advances in the robustness and simplicity of the methods.

## 4. Ventricular volumes and mass

### i. Global volumes, thickness and function

Quantification of cardiac volumes at end-diastole and end-systole, LV mass and global systolic function represent the measurements of cardiac imaging, which are fundamental to decision making in clinical cardiology. These are obtained using standard cine images by CMR and can be processed by virtually every available post-processing software. Cine imaging also supports assessment of regional wall motion abnormalities, either visually or by strain quantification (presently limited to research).

#### Acquisition

- Cine imaging (bSSFP sequences)
- Acquisition defined in SCMR Standardized Protocols [1]:
  ○ complete LV and RV coverage using short axis (SAX) stack of slices
  ○ long axis LV views.
- Older approaches of cine imaging based on fast gradient recalled echo (GRE) sequences: compared to bSSFP sequences, GRE sequences lead to larger LV mass and smaller LV volumes.

#### Post-processing

- Standardised approach defined in SCMR Standardized Post-processing recommendations [2]
  ○ LV mass measurement: inclusion of papillary muscles into LV cavity reduces accuracy compared to autopsy, but results in higher precision (smaller observer variability);
  ○ LV dimensions and wall thickness: most reproducible in 3-chamber view [4];
  ○ An early study showed higher reproducibility of RV volumes; measured in a transverse (TRA) stack [5], a later study reaffirmed that both SAX and TRA are similarly reproducible, as long as both ventricles acquired in entirety [6, 7].
  ○ TRA stack does not support reproducible RV mass measurements [2, 7]. No data available for SAX stack.
  ○ The accuracy and reproducibility of novel post-processing algorithms based on signal intensity thresholding is unknown

#### Validation

- LV mass: excellent validation against gold-standard in animals and excised human hearts after transplantation (Additional file 1: Table 3ai.1).
- LV function/cardiac output: limited validation against invasive conductance catheters [8, 9]

#### Precision

- A large body of evidence exists on interstudy, inter- and intraobserver reproducibility (Additional file 1: Table 3ai.2)
- A body of evidence supports superior precision of CMR-derived measurements compared to:
  - ○ radionuclide ventriculography [10]
  - ○ nuclear medicine techniques (PET, single photon emission computed tomography (SPECT)) [11]
  - ○ transthoracic 2- and 3D echocardiography [12, 13]
  - ○ cardiac CT [14]
- Benchmarking datasets available [15]

#### Normal values

- Normal values available have been derived for various field strengths, imaging sequences, post-processing approaches, age-, sex- and ethnicity groups (Additional file 1: Table 3ai.3), summarised in [16].
- There is moderate variability in normal ranges depending on the population studied and method of quantification.
- SCMR CT WG members recommend the use of normal values that correspond the mode of acquisition and postprocessing (as per SCMR recommendations for acquisition and postprocessing (1,2).

#### Qualification and utilisation

- Diagnostic interpretation and clinical decision making underlying practice guidelines is based on evidence derived with echocardiography. The cut-off values (most notably for LV ejection fraction) have been adopted by other imaging modalities, including CMR. The original evidence base by transthoracic echocardiography has been revalidated and expanded upon by CMR (Additional file 1: Table 3ai.4).
- Abnormal changes in cardiac volumes, function and LV mass indicate the presence of disease and relate to worse outcome. LV volumes and function by echocardiography have been described as the strongest predictor of survival in heart failure (HF) [17–20]. Recent data with CMR LGE (Additional file 2: Table 3b-i.4) and T1-mapping indices (Additional file 3: Table 3c-i.6) show consistently better prognostic predictive value in HF and non-ischemic cardiomyopathy (NICM).

### ii. Regional wall motion and deformation

Myocardial strain imaging enables time-resolved quantification of myocardial contraction and relaxation, which is less influenced by the ventricular pressure/volume loading conditions. Characterisation of these events in various conditions, with aim to better understanding of tissue architecture and the underlying efficiency of deformation, remains an active research domain. Once a complex and often time-consuming acquisition and post-processing, strain imaging with CMR is now made possible using standard cine imaging. Owing to the issues with the reproducibility and an overall lack of incremental diagnostic and prognostic data, the value of strain imaging in clinical use remains investigational.

#### Acquisition

- ○ Cine imaging (feature tracking)
- ○ Tagging (spatial modulation of magnetization-SPAMM, complementary –SPAMM (CSPAMM) [21], harmonic phase image analysis [22])
- ○ Displacement ENcoding with Stimulated Echoes (DENSE) [23]
- ○ Strain encoding imaging (SENC) [24]

#### Post-processing

- ○ Visual segmental analysis [2]
- ○ Regional wall motion score
- ○ Deformation/strain analysis (tagging, feature tracking [25])
- ○ Dyssynchrony [26]
- Results may be presented either segmental (provided for 17 segments as per AHA/ACC) or global (deformation components: longitudinal, radial, circumferential, torsion) values.

#### Validation

- Validation in phantoms and animals
  ○ Tagging is referred to as the reference standard for strain imaging [27] [28, 29](Additional file 1: Table 3ii.1)
  ○ DENSE [30]
- Comparative studies to tagging:
  - ○ DENSE can provide greater reliability and resolution of segmental analysis [30]
  - ○ Feature tracking [25]
- Comparative studies to echocardiography:
  - ○ Strain by DENSE, tagging, feature tracking (reviewed in [31, 32])
  - ○ Dyssynchrony by feature tracking [33]

#### Precision

- Wall motion based on visual assessment of each segment, observer dependent on training and experience [34] (Additional file 1: Table 3ii.2.)
- Reproducibility may be improved with automated processing [27]
- Some studies demonstrated superior precision of tagging [35], however, the endocardial border may be obscured by a tag line prohibiting adequate assessment of wall thickening
- The best spatial resolution for strain imaging is currently given by DENSE [36]. Fast processing methods are available for analysis of strain and displacement [37].
- Feature-tracking reproducibility remains problematic, especially for radial strain [38, 39].
  - ○ Circumferential strain preforms best, if averaged over the whole slice. Regional estimates are more variable (reviewed in).
  - ○ Considerable inter-vendor variability of outputs [39, 40]
- Benchmarking datasets available [41].

#### Normal values

- Normal values available for global strain components (Additional file 1: Table 3ii.3).
- Several studies available for segmental values [42–44], of note, regional values vary significantly in a given heart complicating the definition of normal values and cut-offs.
- One study reported normal values for strain according to segment, age, sex and ethnicity [27]

#### Qualification/utilisation

Strain imaging with CMR remains an exploratory research domain, due to complex and often time-consuming post-processing. Outcome data suggest utility in addition to standard measures of care in clinical management. (Additional file 1: Table 3ii.5)

1. Regional wall motion score is used as a single value to describe wall motion abnormalities or changes during stress testing [45];
2. Outcome data for deformation analysis of high dose dobutamine stress testing with SENC [46];
3. Global longitudinal strain is a better predictor of outcome in DCM than volumes or ejection fraction [47];
4. Intervendor variability of outputs for feature tracking implies that various algorithms may not convey equivalent information.

#### Development directions

1. Standardisation of acquisition and post-processing approaches
2. Development of robust normal values for vendor-specific acquisition/post-processing
3. Establishment of characteristic disease-specific or pathophysiology specific signatures of deformation abnormalities (diagnostic and prognostic relevance)
4. Determination of reversibility of parameters/signatures with treatment
5. Utility in guiding treatment through clinical trials.

### iii. Diastolic function

Assessment of diastolic relaxation is an indirect approach to myocardial tissue characterisation and can be done by CMR by employing analogous approaches to those used in echocardiography. Increased myocardial stiffness commonly coincides with the states of increased LV wall thickness, either due to global pathological myocardial processes, such as accumulation of myocardial fibrosis or amyloid, or due to regional myocardial injury, such as ischaemic scar. Because CMR provides means of direct tissue characterisation, by LGE and T1 mapping, assessment of diastolic function by CMR is not commonly used.

#### Acquisition

- Time resolved curve of left ventricular diastolic filling from cine SAX stack [48, 49]. As in echocardiography, these parameters are dependent on loading conditions.
- 2- and 4-chamber cine views for measurement of left atrial (LA) volume
- Phase-contrast gradient echo sequence acquisitions:
  - ○ Through-plane flow measurement across mitral valve, pulmonary venous inflow (velocity encoding 130 cm/sec)
  - ○ Basal SAX slice measurement of mitral flow and annulus velocities (velocity encoding < 30 cm/sec)
- Tagging [50]

#### Post-processing

- Transmitral E and A waves
- Pulmonary venous inflow S, D and A waves
- Mitral annulus velocity e’
- LA size
- Peak early diastolic strain rate (PEDSR)

#### Validation

- Against PV loops [51]
- Comparative studies with echo [52–54]

#### Normal values

- Normal values available for early diastolic velocities

#### Development directions

1. Standardisation of acquisition and post-processing approaches
2. Development of robust normal values for vendor-specific acquisition/post-processing
3. Establishment of characteristic disease-specific or pathophysiology specific signatures of diastolic abnormalities (diagnostic and prognostic relevance)
4. Determination of reversibility of parameters/signatures with treatment
5. Utility in guiding treatment through clinical trials.

## 5. Tissue characterisation

### i. Late gadolinium enhancement

LGE is a myocardial tissue characterization technique, which demonstrates regional myocardial tissue differences based on differential uptake/washout of gadolinium-based contrast agent (GBCA). LGE is optimally suited to visualize myocardial infarction and scar, as well as areas of regional scar/fibrosis in non-ischaemic cardiomyopathies, such as in hypertrophic and dilated cardiomyopathy, sarcoidosis or myocarditis. In acute myocardial infarction or myocarditis, LGE co-localises with areas of cell-necrosis or oedema. Conversely, LGE can also reveal unenhanced areas in the core of the contrast-enhanced regions, representing either microvascular obstruction (MVO) (a no-(re)flow phenomenon) or intramyocardial haemorrhage (IMH).

#### Acquisition

- Inversion recovery (IR) prepared T1 weighted gradient echo sequences with either individually adapted prepulse delay (‘to achieve myocardial signal nulling’) and/or inline Phase-Sensitive Inversion-Recovery (PSIR)-based reconstruction algorithm [1]
- Acquired as in full LV coverage in short axis and long axis views during mid-diastole
- ~ 10 min delay time from administration of GBCA [1]
- GBCAs lead to:
  - ○ shortening of T1 - > increased signal intensity in areas of intense GBCA accumulation compared to areas with quick wash-out, such as normal myocardium;
  - ○ differential distribution between myocardial regions with intact myocardial cells (membranes) and expanded extracellular space due to necrosis, fibrosis or scar;
  - ○ in amyloidosis, there is commonly poor contrast difference between the blood and myocardium due to expansion of the extracellular volume throughout the myocardium, resulting in lower gradient in GBCA concentration between these two tissues, save for the bright endocardial border;
- Evidence of LGE is a marker of expanded extracellular space, most commonly seen due to necrotic myocardium or scar tissue
- Methods to assess microvascular obstruction (MVO) (and IMH) include [55–57]:
  - ○ first pass perfusion imaging,
  - ○ early IR-TFE imaging (app. 1 min, no ‘nulling’, long prepulse delay > 400 msec)
  - ○ LGE (app. 10–20 min)
  - ○ native T1
  - ○ Contrast-enhanced cine-bSSFP
  - ○ First pass and early hypoenhancement less strongly related to remodelling and clinical outcomes than LGE
- Alternative ways to IMH imaging by T2* (see section Mapping) [58].

#### Post-processing

- Visual assessment reporting on the presence, type (ischemic/non-ischemic), location, and transmurality [2]
- Quantitative assessment (i.e. LGE extent) can be based on several approaches:
  - ○ Manual approach (i.e. visual delineation)
  - ○ full width half maximum (FHWM)
  - ○ The “n”-SD approach (standard deviations, SD): 2SD (for nonischaemic scar)/5SD of the noise (for infarction) above the signal intensity of normal myocardium [2].
  - ○ LGE extent is reported as % of LV mass
- MVO can be measured manually or by SD-thresholds. The strong contrast between scar and MVO results in a highly reproducible delineation [59].

#### Validation

- Excellent validation of LGE imaging for the presence, extent and transmurality of LGE against reference standard for ischemic scar and non-ischemic fibrosis (animal experiments, human endomyocardial biopsies (EMB), explanted hearts) (Additional file 2: Tables 3b-i.1 and 3b-i.2)
  - ○ In acute myocardial infarction, LGE overestimates infarct size (see T2 imaging section) reviewed in [60].
  - ○ CMR favourably compares to alternative techniques (SPECT, PET) due to its higher sensitivity and spatial resolution to resolve infarct transmurality (based on better spatial resolution) (Additional file 2: Table 3b-i.3)

#### Precision

- Large body of evidence on interstudy, inter- and intraobserver variability in acute and chronic ischemic scar as well as in NICMs (Additional file 2: Table 3b-i.2)
- No comparison of precision to SPECT/PET due to the poor interstudy reproducibility of the later methods
- No benchmarking datasets available

#### Normal values

- Normal reference defined as absence of LGE
- Interpretation by pattern (ischemic, non-ischemic, patchy, diffuse), localization (typical coronary artery territory, mid-wall, epicardial, septal, lateral), transmurality (% of wall thickness).

#### Qualification and utilisation (Additional file 2: Table 3b-i.4)

- Excellent diagnostic tool for the determination of chronic myocardial infarction and regional fibrosis in cardiomyopathies.
- Stronger predictor of outcome than LVejection fraction (EF) and LV volumes in chronic stable disease (HF, chronic CAD)
- Stronger predictor of outcome than LV-EF and LV volumes in acute myocardial infarction
- Stronger predictor of malignant ventricular arrhythmia, sudden death and lower likelihood of improvement with medical therapy in various patient groups with cardiomyopathy
- LGE transmurality able to inform on reversibility of underlying regional wall motion abnormality
- MVO and IMH - predictors of poor outcome, but uncertainty whether these are independent [61] of infarct size or interrelated [62] (IMH occurs in a subset of MVO)

#### Development directions

- Standardization of acquisition methods and nulling approaches to achieve similar relative signal-to-noise ratios of fibrotic tissue versus normal myocardium (currently dependent on contrast agent type, dose and time after injection, field strength, type of sequence and other variables including the underlying injury itself).
- Improved definition of transmurality and segmental allocation for visual interpretation
- Standardization of quantification methods for LGE. Studies used the FWHM and the SD-based methods, however, this remains suboptimally standardized in terms of
  - ○ To determine the cut-off value, the method with the best prognostic/ diagnostic value.
  - ○ The different data acquisition techniques and post-processing algorithms may require different post-processing approaches.

### ii. T2 weighted imaging

Myocardial tissue characterization using electrocardiogram (ECG)-triggered T2 weighted (T2W) sequences is used to demonstrate the regional differences in myocardial water content. T2W imaging is optimally suited to visualize regional oedema, such as in acute myocardial infarction, supporting assessment of area at risk (AAR)/myocardial salvage index (MSI). It was also applied in myocarditis imaging, as a part of Lake-Louise Criteria (LLC). Owing to lengthy and artefact-prone acquisitions, T2W imaging is less fit for use in everyday clinical practice, and increasingly replaced by the modern quantifiable acquisition alternatives, T1 and T2-mapping.

#### Acquisition

Several sequences/approaches are available for T2W cardiac imaging [1]:

- ○ T2W black-blood turbo spin echo (T2W-TSE)
- ○ T2W short tau inversion recovery (STIR),
- ○ T2-prepared SSFP
- ○ Emerging new approach for AAR using contrast-enhanced SSFP (based on T2 and T1 contrast) for AAR assessment based on the acquisition of the cine LV stack [63]

- Technical limitations of T2W CMR pulse sequences are susceptible to various influences causing some limitations as endpoints and in clinical practice:
  - ○ long acquisition time over 2 heart beats result in long breath-holds and artefacts due to cardiorespiratory motion;
  - ○ variations in phase array coil sensitivity
  - ○ high signal from slow moving blood (e.g. at the subendocardium and in the ventricular apex)
  - ○ low contrast-noise ratio in differentiating oedematous vs. normal tissue

#### Post-processing

- T2W imaging of AAR/myocardial salvage (**(**Additional file 4: Tables 3b-ii.1–2):
  - ○ Myocardial salvage is calculated by subtraction of percent infarct size (by LGE) from percent AAR (by T2W imaging) [64, 65].
  - ○ MSI is calculated by dividing the salvage area by the AAR.
  - ○ Post processing is subjective (based on ‘n’-SD threshold approaches or visual delineation)
  - ○ Optimal imaging time for AAR assessment is ideally 4–7 days after acute MI [62].
- T2W imaging in myocarditis (LLC) [66]:
  - ○ Visually determined areas of hyperintensity in T2W images
  - ○ Global oedema ratio: semi-quantitative analysis by normalizing the signal intensity of the myocardium to that of skeletal muscle: values of more than 1.9 indicate myocarditis

#### Validation

- Hyperintense signal on T2W CMR has been shown to indicate increased myocardial water content, whereas hypointense signal within the hyperintense injured zone indicates IMH **(**Additional file 4: Table 3b-ii.1)
- Phantom and Tissue studies
  - ○ Proton transverse (T2) relaxation times reflect tissue hydration.
  - ○ Alterations in T2 signal enable visualisation of regional myocardial oedema as area of hyperintense signal
- Animal models
  - ○ The ischemic AAR consists of oedema and is typically greater than infarct size - > T2W imaging represents a non-invasive approach to AAR estimation.
  - ○ T2W imaging enables retrospective determination of the ischaemic area-at-risk
  - ○ Comparison of contrast-enhanced bSSFP with myocardial perfusion SPECT
- Human studies
  - ○ Dynamic changes of AAR after acute myocardial infarction [65, 67, 68]
  - ○ Comparison of T2W AAR by CMR with myocardial perfusion SPECT
  - ○ Comparison of contrast enhanced bSSFP with myocardial perfusion SPECT [63]
  - ○ LLC vs. EMB-criteria for myocarditis (Additional file 4: Table 3b-ii.1)

#### Precision

- Available data on reproducibility of T2W imaging for AAR [69, 70]
- Comparison of T2W vs. T2 mapping for AAR reveals T2 mapping to be more reproducible [71];
- Comparison of seven post-processing approaches for quantifying oedema in T2W imaging in acute MI (2 SD, 3 SD, 5 SD, Otsu, FWHM, manual threshold, and manual contouring) revealed that manual contouring provided the lowest inter, intraobserver, and interstudy variability for both infarct size and oedema quantification [72].
- The FWHM method for infarct size quantification and the Otsu method for myocardial oedema quantification are acceptable alternatives [72].
- No data available for contrast-enhanced bSSFP
- No data available for oedema ratio in myocarditis

#### Normal values

- Normal reference = absence of hyperintense signal (poor negative predictive value)
- In myocarditis: semiquantitative ‘oedema ratio’ of < 1.9 (SI of myocardium/SI of skeletal muscle) [66]

#### Qualification and utilization

- ○ Detection of myocardial damage in patients with acute coronary syndrome (ACS) [73]
- ○ Determination of salvaged myocardium in STEMI patients, prediction of higher revascularisation rate and adverse prognosis (Additional file 4: Table 3b-ii.4).
- ○ Randomised controlled trials using T2W and contrast-enhanced bSSFP AAR as an endpoint (ischaemic preconditioning [74, 75].
- ○ T2-oedema ratio variable sensitivity across inflammatory cardiomyopathies with moderate positive and poor negative predictive value (Additional file 4: Table 3b-ii.3)
- ○ No prognostic or therapeutic studies

#### Development directions

- Native T2 mapping methods enable quantification of T2 relaxation times and are less susceptible to artefacts (see chapter on T2 mapping)
- The utility of T2W-imaging in excluding ACS in the emergency room has become less prominent since the advent of high-sensitivity troponin assays.

## 6. Quantitative tissue characterisation

### i. T1 mapping

T1 mapping is a quantitative tissue characterization technique, which allows quantifying the rate of longitudinal relaxation of myocardial tissue (and blood). The resulting measurements come as an absolute number (time, ms), which is sequence-specific, requiring standardization of acquisition and calibration of values in health and disease.

#### Acquisition

Sequences allowing acquisition of a series of images (using increasing time delays) during the evolution of the longitudinal relaxation:

- Acquisition in mid-diastole (owing to a lengthy image acquisition time, systolic acquisition window less robust)
- Magnetisation preparation by inversion (180°) or saturation (90°) prepulses
- Several imaging schemes with differences in number of images, pauses for magnetization recovery (which can be defined as either beats or seconds), flip angles, use of adiabatic prepulses, acceleration techniques (half-scan, partial Fourier)
  - ○ resulting in differences in T1 accuracy (with consequences for precision and diagnostic accuracy, see below).
- Native (without GBCA) and post-contrast T1 mapping (typically ~ 10–15 min after administration of GBCA, dose and type of GBCA not standardized)
- Single (midventricular) short axis slice (− > diffuse myocardial disease) or three short axis slices (apical, midventricular, basal) (− > regional myocardial disease)
- Heart-rate dependency:
  - ○ for myocardial T1 with most sequences not relevant within physiological heart rates < 80 bpm;
  - ○ less important for post-contrast images, more relevant for long T1 values such as native blood, or in very severe myocardial disease (amyloidosis, severe oedema), as more time needed for full relaxation;
  - ○ every other beat acquisition (2RR intervals) may be used in tachycardia
- Extracellular volume fraction (ECV) calculation based on pre- and postcontrast T1 mapping acquisitions and blood values which requires standardization and heart rate correction for each component (unclear: identical sequences/ different schemes for pre/postcontrast acquisitions)

#### Post-processing

- Pixel-wise image reconstruction and exponential curve-fitting of signal intensity values (3-parameter fitting model):
  - ○ magnitude image detection: SMAG(t) = abs(A – B exp.(−t/T1*)
  - ○ phase sensitive inversion recovery (PSIR) detection : SPSIR(t) = A – B exp.(−t/T1*).
- T1 value equates with the time at 63% recovery of longitudinal magnetisation
- Post-processing affected by the regional variation due to variable sensitivity of phase array coils and artefacts in the lateral wall (septal region of interest (ROI) more precise compared to SAX ROI, segmental values difficult to normalise)
- Myocardial ROI placement:
  - ○ diffuse (global) myocardial involvement:
    - ▪ septal ROI [76] or SAX ROI [77, 78]
    - ▪ excluding areas of LGE
    - ▪ conservatively within the myocardium [76]
  - ○ regional myocardial T1 values:
    - ▪ Segmental ROI placement [79]:
    - ▪ Significant regional differences in T1 values - > difficulty in normalising segmental T1 values
    - ▪ No significant differences between septal values for basal and midventricular slice, however considerable overestimation in apical slice (due to partial volume)
- Blood ROI placed in the centre of the blood pool using same acquisition as above, avoiding papillary muscles
- Post-contrast T1 blood: less in-flow effect compared to native T1
- T1 indices (Additional file 3: Table 3c-i.1):
  - ○ direct measurements: native T1, post-contrast T1
  - ○ calculated indices based on pre- and post-contrast T1 mapping acquisitions:
    - ▪ lambda (partition coefficient of contrast agent distribution between blood and myocardium) = (R1myocardiumnative-R1myocardiumpost-contrast)/ (R1bloodnative- R1bloodpost-contrast), where R1 = 1/T1
    - ▪ ECV is partition coefficient lambda, which relates to the extracellular space only, by accounting for intracellular space of blood by haematocrit (requires contemporaneous blood sampling): =(1-Ht)x lambda
    - ▪ synthetic ECV calculation
      • derived from a relationship between haematocrit and longitudinal relaxation rate of blood (R2 = 0.51)
      • derived for selected sequences and a single vendor only [80, 81]

#### Validation

- Phantom sequence characterisation (T1 accuracy and precision)
- Histological correlation with collagen volume fraction (Additional file 3: Table 3c-i.2)
  - ○ Correlations vary between reports
  - ○ Differences in staining techniques, inclusion/exclusion of areas of LGE
  - ○ Differences in sequence parameters, type/dose/timing of GBCA, different sequences used in pre/postcontrast acquisitions, post-processing (ROI placement)
- Several components of the measured values do not occur in phantoms, such as magnetisation transfer, water-exchange, T2 sensitivity, inflow effects, making a full validation in phantoms or ex-vivo impossible

#### Precision

- Evidence on interstudy, inter- and intraobserver variability (Additional file 3: Table 3c-i.3)
- No benchmarking datasets available as variability seems mainly dependent on data acquisition

#### Normal values

- Some sequences have established normal values (Additional file 3: Table 3c-i.4). Of note, every specific implementation may yield slightly different values and requires standardization.

#### Qualification and utilisation

Abnormal (raised) myocardial T1 values indicate the presence of diseased myocardium and relate to worse outcome:

- Disease models: myocarditis, non-ischaemic dilated cardiomyopathy, amyloidosis, ischaemic cardiomyopathy, hypertrophic cardiomyopathy (Additional file 3: Table 3c-i.5)
- Outcome data (Additional file 3: Table 3c-i.6): T1 values are stronger predictor of outcome than LV function, volumes and LGE:
  - ○ Amyloidosis
  - ○ Non-ischaemic dilated cardiomyopathy
  - ○ Mixed patient cohorts

#### Development directions

- Sequence and vendor specific standardization of acquisition, normal values and calibration of values in health and disease
- Outcome data
- Data on guiding management
- Pre- and post-contrast T1 mapping acquisitions
  - ○ unclear whether identical sequences or different schemes for pre/post-contrast acquisitions amount to a justifiable counterpart of pre/post-contrast acquisition, but rather a mix of poorly related diagnostic tests.

### ii. T2 mapping

T2 mapping is a quantitative tissue characterization method mainly reflecting the water content of the myocardium. It is based on a series of images with different time delays acquired during the diastolic standstill to map the T2 magnetization decay. Increasing evidence base for T2 mapping supports its utility in detection of myocardial inflammation and oedema, in myocarditis and in assessment of acute myocardial infarction (AMI). Myocardial T2 values were shown to decrease with anti-inflammatory treatment.

#### Acquisition

- Sequences acquiring separate images during the evolution of the transverse relaxation in diastolic standstill
- T2 prepared spin echo sequences with several different schemes with differences in number of image acquisitions, flip angles, accelerating techniques, single-shot vs. multiecho
  - ○ T2-prepared bSSFP sequence
  - ○ T2-hybrid gradient echo and spin echo (GraSE) sequence
- Native acquisition (no contrast agent)
- Single (midventricular) short axis slice (− > diffuse myocardial disease) or three short axis slices (apical, midventricular, basal) (− > regional myocardial disease)
- No regional variation due to variable sensitivity of phase array coil

#### Post-processing

- Pixel-wise image reconstruction and exponential curve fitting
- T2 value equates with the time at 63% decay of transverse magnetisation, direct myocardial measurement
- ROI placement:
  - ○ Myocardial septal ROI or SAX ROI (in case of diffuse myocardial disease)
  - ○ Segmental ROI placement (in case regional T2 values are desired)
- No significant difference in segmental values for basal and midventricular slice, overestimation in apical slice (partial volume)

#### Validation

- Phantom sequence characterisation (T2 accuracy and precision)
- Histological validation (Additional file 5: Table 3c-ii.1).
- in animal models
  - ○ AAR
  - ○ AMI reperfused and non-reperfused
    - ▪ Detection of AAR (comparison with T2W/LGE approach as reference standard)
- Model diseases: AMI, myocarditis, transplant rejection
- Validation of water component difficult in animals and biopsies
- Has been shown to be superior to T2W imaging and Lake-Louise Criteria for the diagnosis of acute myocarditis (Additional file 5: Table 3c-ii.2)

#### Precision

- Interstudy, inter- and intraobserver variability (Additional file 5: Table 3c-ii.3)
- No benchmarking datasets available as variability seems mainly due to data acquisition

#### Normal values

- Several sequences have established normal values (Additional file 5: Table 3c-ii.4)

#### Qualification and utilisation

- T2 mapping useful in detecting myocardial oedema and inflammation (Additional file 5: Table 3c-ii.5)
  - ○ Acute myocardial infarction
  - ○ Myocarditis, inflammatory cardiomyopathies, including cardiac involvement in systemic inflammatory diseases, tako-tsubo cardiomyopathy
  - ○ Early detection of ongoing inflammation with the possibility of reversal of myocardial damage using anti-inflammatory intervention may be feasible.
  - ○ Added value to T1 mapping in inflammatory conditions, by informing on active inflammation and reversal upon anti-inflammatory intervention [82–84]

### iii. T2* mapping

T2* mapping is a quantitative test for the assessment of myocardial iron load in patients with thalassemia. T2* mapping is also employed for visualisation of IMH, such as during or after an acute ischaemic event.

#### Acquisition

- As per SCMR protocols:
  - ○ single breath-hold multiecho T2* gradient echo sequences (black-blood prepulse) [1]
  - ○ a single midventricular SAX slice - > septal T2*measurement
  - ○ 3 short axis slices - > global T2* measurement
- Each sequence yields sequence specific “absolute” T2* values (in ms)
- Each sequence requires standardisation (validation, normal values, clinically relevant cut-off values) prior to clinical use
- Only 1.5 T and 3 T datasets are validated for measurement of cardiac iron content [85, 86];

#### Post-processing

- As per SCMR standardised image interpretation and post-processing [2]
- T2* values = the time delay taken for decay of the myocardial signal by 63%
- ROI placement:
  - ○ Myocardial septal ROI in midventricular slice encompassing both epicardial and endocardial borders, to prevent the epicardium-endocardium heterogeneity of iron deposition (informs on global myocardial iron content)
  - ○ Segmental ROI placement (− > regional T2* values)
  - ○ Complete SAX coverage in basal, midventricular and apical slice generates a global T2* value, however, this is less frequently used.
- Significant differences in segmental values reported. Regional variation most likely due to variable sensitivity of phase array coils to different regions.

#### Validation

- Validation against myocardial and liver iron content (Additional file 6: Table 3c-iii.1 and 2)
  - ○ Ex vivo histological validation - > good correlation of T2* measurements versus chemically assayed iron
  - ○ Biopsy not useful as reference standard
  - ○ Therapy guidance by T2* imaging is superior to other tests – as such T2* in thalassemia can be regarded as the clinical reference standard

#### Precision

- Evidence on interscanner, intercenter, interstudy, inter- and intraobserver variability (Additional file 6: Table 3c-iii.3)

#### Normal values

- Iron loading:
  - ○ Normal values for 1.5 T (Additional file 6: Table 3c-iii.4)
  - ○ Established clinically relevant cut-offs of significant myocardial iron loading (septal ROI):
    - ▪ T2* < 20 ms: clinically relevant myocardial iron loading
    - ▪ T2* < 10 ms: severe myocardial iron loading
- IMH - intramyocardial hemorrhage
  - ○ A myocardial region of interest with a T2* < 20 ms is taken to represent haemorrhage
  - ○ T2* (< 20 ms) is highly discriminative of haemorrhagic transformation within the infarct zone vs. infarct zone without haemorrhage or the remote zone

#### Qualification and utilisation

- T2* useful in detecting relevant cardiac iron overload involvement in thalassemia major – > at T2* < 20 msec
- In cardiac iron loading T2* correlates closely with
  - ○ negative cardiac remodelling (ejection fraction, end-diastolic volume (EDV)) and diastolic dysfunction (Additional file 6: Table 3c-iii.2)
  - ○ clinical events (HF, arrhythmia) and
  - ○ response to iron-depletion treatment (Additional file 6: Tables 3c-iii.5 and 6)
- Comparison with historical data indicate improve survival of patients at risk of iron overload due to cardiac T2* mapping guided iron-depletion therapy [87]
- Interdisciplinary consensus statements recommend surveillance of patients at risk of cardiac iron overload using cardiac T2* mapping [88]
- Serial changes in myocardial oedema and haemorrhage in ischaemic and remote zone after reperfusion [62]
- IMH detection by T2* core is independently associated with adverse LV remodelling, major adverse cardiac events and mortality [89]

## 7. Stress myocardial perfusion with CMR

Myocardial perfusion CMR testing under the effect of pharmacological agents (myocardial stress-perfusion) is used to demonstrate regional reduction in myocardial perfusion to assess the presence of hemodynamically significant coronary artery stenosis. Myocardial stress perfusion CMR imaging is a routinely used diagnostic test in patients presenting with symptoms and signs of stable angina. It is also used in patients with medium-to-high pretest likelihood of significant (flow-limiting) coronary artery disease (CAD), patients with known coronary artery stenosis to assess significance of specific lesion(s) and patients with previous revascularization or myocardial infarction. Myocardial stress perfusion CMR imaging can also demonstrate the presence of microvascular disease e.g. in patients with angina and normal coronary arteries. In this case, the imaging abnormality during stress testing is typically hypoperfusion of the sub-endocardium with a circumferential distribution. Myocardial stress perfusion CMR imaging may be useful to demonstrate potential CAD aetiology in patients with HF with or without reduced LV ejection fraction. Quantitative perfusion imaging is increasingly becoming available.

### Acquisition

- Acquisition as per SCMR Standardized Protocols [1]
  - ○ Dynamic acquisition during the passage of the contrast agent bolus (dose 0.05–0.1 mmol/kg body weight) through the left ventricular cavity and the myocardium;
  - ○ First pass acquisition during pharmacological stress;
  - ○ Repeat pass acquisition at rest (may be omitted for qualitative assessment, if LGE is available to determine infarction)
  - ○ 3 short axis slices (basal, midventricular and apical) every heart beat for a minimum of 40–50 heart beats→ a minimum of 40–50 dynamic measurements);
  - ○ 3D whole heart acquisition methods available, currently no demonstrated diagnostic advantage over 2D 3-SAX slice acquisition [90];
- Sequences: various sequences available based on saturation prepulse for preparation of magnetization and acquisition of data with either:
  - ○ spoiled fast gradient echo (GrE);
  - ○ bSSFP pulse sequences;
  - ○ typically combined with acceleration techniques:
    - ▪ echo planar imaging (GrE-EPI);
    - ▪ spatial undersampling (e.g. sensitivity encoding (SENSE)
    - ▪ generalized autocalibrating partially parallel acquisitions (GRAPPA),
    - ▪ spatio-temporal undersampling (e.g. k-t Broad Linear Speed up Technique, k-t BLAST or k-t SENSE);
  - ○ differing in the acquired spatial (3x3x8 mm to 1.3 × 1.3x8mm) resolution.
- Diagnostically relevant is the stress acquisition.
- The rest acquisition is used to:
  - ○ discern possible artefacts
  - ○ to support calculation of parameters based on the change of stress and rest perfusion (e.g. myocardial perfusion reserve index - MPRI or myocardial blood flow – MBF - reserve).
- Pharmacological stress is the standard approach (adenosine, regadenosone, dobutamine); exercise stress has shown feasibility in research settings.

### Post-processing/interpretation

- as per SCMR Standardized Postprocessing [2]
  - ○ Visual interpretation is the standard clinical approach:
  - ○ Hypoperfusion is defined as segmentally reduced contrast agent uptake at peak stress persisting for 5 consecutive heart beats
    - ▪ not present at rest,
    - ▪ outside the enhanced myocardium on LGE images.
  - ○ several diagnostic standards of test positivity proposed demonstrating a reduced increase of flow or reduced peak flow in areas subtended by vessels with significant coronary stenosis (see below)
- The benefits of quantitative and semi-quantitative over qualitative interpretation remain at present investigational. Quantitative and semi-quantitative evaluation require:
  - ○ stress and rest acquisition to calculate perfusion reserve or perfusion reserve indices; peak perfusion can be determined from stress images only
  - ○ dual bolus, dual contrast sequences or other algorithms to correct for the non-linearity of signal intensity and contrast agent at higher doses
  - ○ correction for baseline signal differences
  - ○ efficient motion correction
  - ○ a myocardial perfusion reserve index (MPRI) can be calculated from various parameters, usually using the relative upslope between rest and stress (corrected for changes of upslope of the contrast bolus in the left ventricle)
  - ○ full quantification can be achieved with various mathematical algorithms (e.g. Fermi deconvolution, Patlak plot).

### Validation

- Excellent validation of technique (Additional file 7: Table 3d.1 and 2):
  - ○ Flow in phantoms and microspheres in animals;
  - ○ Comparative effectiveness diagnostic studies and meta-analyses studies of CMR to PET, SPECT, invasive coronary angiography and invasive flow measurements (fractional flow reserve – FFR; Additional file 7: Table 3d.1). Favorable results in comparison to SPECT due to higher spatial resolution. [91, 92]
  - ○ Outcome studies of stress myocardial perfusion CMR imaging validating predictive association of positive and negative outcome (Additional file 7: Table 3d.3), similar results to SPECT.
  - ○ Quantitative perfusion imaging: many approaches require more validation, especially as large outcome studies have been performed with visual analysis

### Precision

- Limited evidence on interstudy, inter- and intraobserver reproducibility due to need of stress and contrast injection (Additional file 7: Table 3d.2)

### Normal values

- Limited data on normal values due to lack of standardization of image acquisition and post-processing
- Visual assessment: several diagnostic standards on the interpretation of the presence/severity/prognostic relevance of myocardial hypoperfusion based on number of affected segments:
  - ○ ESC guidelines on stable CAD (16 segment ACC/AHA segmentation) [93]:
    - ▪ ≤2/16 segments indicate a good prognosis with optimal medical therapy (OMT) (negative test)
    - ▪ ≥ 3/16 segments defined as prognostically relevant (warrants attempt to revascularize on prognostic grounds (positive test)
  - ○ Subsegmentation into 32 segments (with endo- and epicardial division):
    - ▪ ≤ 3/32 segments indicate a good prognosis with OMT
    - ▪ ≥4/32 segments defined as prognostically relevant [94]
  - ○ MR-INFORM: prognostically relevant ischaemia [95]:
    - ▪ Either transmural hypoperfusion defect or perfusion defect affecting 2 slices or > 60% in basal and midventricular slice, > 90% in apical slice.
- (Semi-) quantitative assessment of MBF, MPR or MPRI: experimental data available for different field strengths

### Qualification and utilisation

- Data from prospective observational studies using stress myocardial perfusion CMR have shown significant predictive association for the presence/severity of myocardial ischemia with outcome **(**Additional file 7: Table 3d.3):
  - ○ Effective cardiac risk reclassification in patients with known or suspected stable CAD [96].
  - ○ Excellent negative predictive value - > low event rate in patients with a negative test
  - ○ Excellent positive predictive value substantiating the role for revascularisation following positive test to improve prognosis
  - ○ Improvement of MPR after percutaneous coronary intervention (PCI)

### Development directions

- Standardization of post processing methods (semi quantitative and quantitative) to allow definition of normal values, effect sizes and improvement of reproducibility. Different post processing methods may apply for different data acquisition techniques.
- Improvement of spatial resolution / coverage based on faster acquisition techniques (e.g. compressed sensing)
- Quantitative perfusion imaging is increasingly becoming available. At this stage the various approaches require more validation, especially as large outcome studies have been performed with visual analysis.

## 8. Vascular endpoints

CMR allows a comprehensive assessment structure and function of the great vessels by anatomical assessment of vessels dimensions and cross-sectional areas, functional assessment of the vessel wall (aortic strain and distensibility, and central (aortic) pulse wave velocity (PWV)). Tissue characterisation by T1-, T2- and proton density-weighted imaging and, more recently, by T2 mapping allows characterization of tissue composition.

### Acquisition

Acquisition as per SCMR standardised protocols

- Anatomy and dimensions:
  - ○ cardiac triggered-contrast enhanced CMR angiography
  - ○ free-breathing 3D balanced acquisition.
- Wall thickness and wall volume by black blood CMR
- Distensibility and strain: balanced (cine) image acquisitions orthogonal to the vessel of interest;
- PWV: measurement of pulse wave travel time/path between ascending and descending aorta
  - ○ ‘through plane’ flow acquisitions in ascending or descending aorta and an anatomical image of thoracic aorta.
  - ○ ‘inplane’ velocity acquisition of thoracic aortic candy-cane
- Wall tissue characterisation by:
  - ○ T2 weighted sequences
  - ○ T1 inversion recovery GRE sequences (vessel wall gadolinium enhancement)

### Post-processing

- Inner-vessel diameters, cross-sectional areas
- PWV: travelled path divided by time delay.
  - ○ Foot-to-foot
  - ○ Upslope measurements
- Tissue characterisation
  - ○ Visual assessment
  - ○ Contrast-to-noise (CNR) measurements

### Validation

- Comparative studies for aortic PWV and distensibility with alternative techniques, including invasive and tonometric PWV measurements (Additional file 8: Table 3e.1)
- T2 mapping vs. histology of carotid specimens showing accurate quantification of plaque lipid content and the different plaque composition despite similar grade of stenosis (Additional file 8: Table 3e.1).

### Precision

- Limited evidence on interstudy reproducibility of anatomical and tissue measurements
- Excellent evidence for PWV: measurements highly reproducible

### Normal values

- CMR data from large healthy populations are available for different anatomical and functional measurements adjusted by age, sex and body mass index (BMI) (Additional file 8: Table 3e2).

### Qualification and utilisation

- Guiding management in aortic dilatation and aortic valve replacement (Additional file 8: Table 3e2)
- Aortic wall imaging and PWV serve robust biomarkers of cardiovascular risk

### Development directions

- Functional assessment of reactive ischemia (or oximetry after cuff induced limb ischaemia) or exercise-induced blood flow in peripheral artery disease [97, 98]

## Conclusion

Not applicable.

## Additional files

Additional file 1: Ventricular volumes and function. **Table 3i.1:** Validation ex-vivo CMR cine imaging for ventricular volumes, mass and function. **Table 3i.2:** Reproducibility of the measurement of LV and RV in healthy volunteers. **Tables 3i.3:** Normal values. **Table 3i.4:** Outcomes studies against clinical endpoints for LV (Table A) and RV (Table B) function, mass or volumes. **Table 3ii.1:** Validation studies and comparative studies of strain imaging. **Table 3ii.2.** Reproducibility values reported for main strain measurements. **Table 3ii.3:** Normal values reported by different studies for main strain measurements. **Table 3ii.4:** Outcome studies for RWMA and strain parameters. (DOCX 101 kb)

Additional file 2: Late gadolinium enhancement. **Table 3b-i.1:** Validation studies with LGE. **Table 3b-i.2:** Reproducibility of measurements for LGE. **Table 3b-i.3:** Comparative studies with other imaging techniques in ischaemic heart disease. **Table 3b-i.4:** Outcome studies with LGE. (DOCX 79 kb)

Additional file 3: T1 mapping tables. **Table 3c-i.1:** Overview of the T1 mapping indices. **Table 3c-i.2:** Histological correlations with T1 mapping indices in various cardiac conditions. **Table 3c-i.3:** Intra, interobserver and interstudy variability reported for various sequences and field strengths. **Table 3c-i.4:** Overview of studies reporting normative ranges for T1 mapping indices. **Table 3c-i.5:** Proof of concept studies using T1 mapping in health and disease. **Table 3c-i.6:** Outcome studies for all-cause mortality (A) and composite cardiac/heart failure (B) endpoints. (DOCX 113 kb)

Additional file 4: T2 weighted imaging. **Table 3b-ii.1:** Validation studies with T2 weighted imaging (T2W). **Table 3b-ii.2:** Correlations with other relevant parameters for T2W-AAR. **Table 3b-ii.3:** Proof of concept studies using T2W imaging in health and disease. **Table 3b-ii.4:** Outcome studies for all-cause mortality or major adverse cardiac events (MACE). (DOCX 53 kb)

Additional file 5: T2 mapping Tables. **Table 3c-ii.1:** Correlation of T2 mapping indices with histological substrates. **Table 3c-ii.2:** Correlation of T2 mapping indices with other imaging biomarkers. **Table 3c-ii.3:** Reproducibility for native T2 using various sequences and field strengths. Studies included if reported interstudy reproducibility. **Table 3c-ii.4:** Normal values for native T2 reported for different sequences and magnetic fields. Studies included if n>50 subjects. **Table 3c-ii.5:** Proof of concept studies with T2 indices differentiating between health and disease. Studies included if n>25 per patients’ group (unless the only study published). (DOCX 46 kb)

Additional file 6: T2* mapping tables. **Table 3c-iii.1:** Correlation of T2* mapping indices with histological substrates. **Table 3c-iii.2:** Correlation of myocardial native T2* mapping with other imaging biomarkers. **Table 3c-iii.3:** Intra, interobserver and interstudy variability reported for native T2* using various sequences and field strengths. Studies reported if included interstudy reproducibility. **Table 3c-iii.4:** Normal values for myocardial and liver native T2* reported for different sequences and magnetic fields. **Table 3c-iii.5:** Proof of concept studies with T2* indices differentiating between health and disease. **Table 3c-iii.6:** Outcome studies and treatment comparisons’ studies using T2* indices. (DOCX 66 kb)

Additional file 7: Myocardial perfusion imaging with CMR. **Table 3d.1:** Studies comparing CMR perfusion against microspheres and alternative diagnostic approaches. **Table 3d.2:** Interstudy reproducibility and normal values of myocardial perfusion reserve (index). **Table 3d.3:** Prospective outcome studies using stress CMR. (DOCX 107 kb)

Additional file 8: Vascular CMR measurements. **Table 3e.1:** Comparative (validation) studies for CMR vascular endpoints with alternative techniques. **Table 3e.2:** Reproducibility of vascular endpoints. **Table 3:** Normal values for aortic dimensions (Table A), PWV and aortic distensibility (Table B) with CMR according to age and sex. **Table 4:** Outcome studies with PWV with CMR confirming the predictive associations. (DOCX 49 kb)

## Abbreviations

AAR: area at risk
ACS: Acute coronary syndrome
AHA/ACC: American Heart Association/American College of Cardiology
BMI: Body-mass index
CAD: Coronary artery disease
Cardiac CT: Computed tomography
CNR: Contrast-noise-ratio
C-SPAMM: Complementary- spatial modulation of magnetization
CT: Clinical trials
CVD: Cardiovascular disease
DCM: Dilated cardiomyopathy
DENSE: Displacement ENcoding with Stimulated Echoes
ECV: Extracellular volume fraction
EDV: End-diastolic volume
EMB: Endomyocardial biopsy
ESC: European Society of Cardiology
FFR: Fractional flow reserve
FWHM: Full width half maximum
GBCA: Gadolinium-based contrast agent
GRAPPA: Generalized auto-calibrating partially parallel acquisitions
GRaSE: T2-hybrid gradient echo and spin echo
GRE: Fast gradient recalled echo
HF: Heart failure
Ht: Haematocrit
IMH: Intramyocardial haemorrhage
IR: Inversion-recovery
IR-TFE: Inversion-recovery turbo field echo
LA: Left atrium
LGE: Late gadolinium enhancement
LLC: Lake Louise criteria
LV: Left ventricle/left ventricular
LV-EF: Left ventricle ejection fraction
MBF: Myocardial blood flow
MI: Myocardial infarction
MPR: Myocardial perfusion reserve
MPRI: Myocardial perfusion reserve index
MRI: Magnetic resonance imaging
MSI: Myocardial salvage index
MVO: Microvascular obstruction
NICM: Non-ischemic cardiomyopathy
OMT: Optimal medical therapy
PCI: Percutaneous coronary intervention
PEDSR: Peak early diastolic strain rate
PET: Positron emission tomography
PSIR: Phase-Sensitive Inversion-Recovery
PV: Pressure-volume
PWV: Pulse wave velocity
ROI: Region of interest
RV: Right ventricle/right ventricular
SAX: Short axis stack
SCMR: Society for Cardiovascular Magnetic Resonance
SD: Standard deviation
SENC: Strain encoding imaging
SENSE: Sensitivity encoding
SPAMM: Spatial modulation of magnetization
SPECT: Single photon emission computed tomography
SSFP: Steady-state free precession
STEMI: ST-elevation myocardial infarction
STIR: T2-weighted short tau inversion recovery
T2W: T2-weighted imaging
T2W-TSE: T2-weighted black-blood turbo spin echo
TRA: Transverse stack
