# Supplementary material for: Society for Cardiovascular Magnetic Resonance (SCMR) expert consensus for CMR imaging endpoints in clinical research: part I - analytical validation and clinical qualification
Source: J Cardiovasc Magn Reson. 2018 Sep 20;20:67. doi: 10.1186/s12968-018-0484-5 (PMC6147157; doi:10.1186/s12968-018-0484-5)
Supplement: Supplementary file 1 — Ventricular volumes and function. Table 3i.1: Validation ex-vivo CMR cine imaging for ventricular volumes, mass and function. Table 3i.2: Reproducibility of the measurement of LV and RV in healthy volunteers. Tables 3i.3: Normal values. Table 3i.4: Outcomes studies against clinical endpoints for LV (Table A) and RV (Table B) function, mass or volumes. Table 3ii.1: Validation studies and comparative studies of strain imaging. Table 3ii.2. Reproducibility values reported for main strain measurements. Table 3ii.3: Normal values reported by different studies for main strain measurements. Table 3ii.4: Outcome studies for RWMA and strain parameters. (DOCX 101 kb) [file 12968_2018_484_MOESM1_ESM.docx]

## Ventricular volumes and function

**Table 3i.1: Validation ex-vivo CMR cine imaging for ventricular volumes, mass and function.** Values are expressed as mean difference± standard deviation (MD±SD) between CMR derived measurements and the reference standard. Coefficient of variation (CoV, value in brackets) is derived from SD of the measurements, divided by the mean value, expressed as %. SSFP: steady-state free precession. TGrE = segmented gradient echo. *Value for Cine in diastole (as per recommended approach).

| **Study** | **CMR Method of cine imaging** | **N** | **Weighing method / reference standard** | **LV mass (g)** | **RV mass(g)** |
| --- | --- | --- | --- | --- | --- |
| **Animal models** | | | |  | |
| Fieno[1] | SSFP, 1,5T, In vivo CMR (dogs) | 10 | water displacement | 1.8±4.1* |  |
| Lorenz[2] | SSFP, 1.5T, In vivo CMR (dogs) | 10 | water displacement | 5.0±7.7 (7.8) | 4.6±6.3(18) |
| Childs[3] | SSFP, 1.5T, Ex vivo CMR (dogs) | 12 | mould displacement | 7.47±5.57 (3.49) |  |
|  |  |  |  | **LV Volumes (mL):** -1.6 ± 1.8 (3.63) | |
| Codella[4] | SSFP, 1.5T, In vivo CMR (dogs+pigs) | 10 | water displacement | 1±3 (4.3) |  |
| Gilbert[5] | SSFP, 3.0T, ex vivo CMR (sheep) | 10 | weight | 5.76± 3.68 (3.9) | 6.73 ± 4.41 (13) |
| **Excised human hearts** | | | |  | |
| Farber[6] | SSFP, 1,5T, Ex vivo CMR (explanted hearts) | 55 | weight | -16±33.7(10.9) | 19±31.1(22.5) |
| **Function** | | | |  | |
| Lin[7] | TGrE, 3.0T in vivo pigs | 14 | conductance catheter | **Cardiac output (l/min):**  -0.05 (r^2^=0.85) | |

**Table 3i.2. Reproducibility of the measurement of LV and RV in healthy volunteers.** Studies included if reporting for interstudy reproducibility. Values represent mean difference and standard deviation between two measurements (MD±SD) and coefficient of variation (CoV, derived from SD of the measurements divided by the mean value, expressed as %). LV: left ventricle. RV: right ventricle. EDV: end-diastolic volume. ESV: end-systolic volume. EF: ejection fraction

|  | **Hudsmith****[8]** | **Grothues [9,10]** |
| --- | --- | --- |
| **N** | 12 | 20 |
| **Method** | SSFP | SSFP |
| **Field strength (T)** | 1.5 | 1.5 |
| **Interstudy reproducibility** |  | |
| LV-EDV | -2.6±7.9 (5.2) | 2.2±4.3 (2.9) |
| LV-ESV |  | 1.5±2.8 (6.5) |
| LV-EF | 0.5±4.9 (7.5) | -0.5±1.7 (2.4) |
| LV-mass | 1.8±10.1 (9.4) | -1.1±4.2 (2.8) |
| RV-EDV | 1.3±20.7 (7.4) | 1.1±6.5 (4.2) |
| RV-ESV |  | -0.3±4.7 (8.1) |
| RV-EF | 1.9±6.8 (11.4) | 0.6±2.7 (4.3) |
| RV-mass |  | -0.4±4.7 (7.8) |
| **Interobserver variability** |  | |
| LV-EDV | 0.8±3.9 (2.7) |  |
| LV-ESV |  |  |
| LV-EF | 1.6±2.2 (3.3) |  |
| LV-mass | 5.8±5.2 (5.2) |  |
| RV-EDV | -0.2±16.0 (9.6) |  |
| RV-ESV |  |  |
| RV-EF | -2.8±6.3 (10.7) |  |
| RV-mass |  |  |
| **Intraobserver variability** |  | |
| LV-EDV | 8.6±8.6 (5.6) |  |
| LV-ESV |  |  |
| LV-EF | 0.5±1.5 (2.3) |  |
| LV-mass | 5.4±6,2 (6.1) |  |
| RV-EDV | -6.0±15.0 (9.0) |  |
| RV-ESV |  |  |
| RV-EF | 0.1±3.2 (5.3) |  |
| RV-mass |  |  |

**Tables 3i.3: Normal values.** Table A provides an overview of the studies reporting normal values for measurements of LV and RV obtained in healthy subjects (except for Kawut et al [17] including MESA population, patients without known CV disease but including CV factors). Studies using standard balanced steady-state free precession sequences (SSFP) are shown, except for MESA studies, who used a fast gradient echo sequence (FGE). Postprocessing approaches differed as to the inclusion or exclusion of papillary muscles (PMi or PMe) as a part of the blood volume. Tables B and C list the studies reporting normal ranges (mean±SD) for LV and RV, in males (M) and females (F), respectively. Following units are used: EF %; EDV, ESV, SV ml; EDVi, ESVi, SVi ml/m2; mass g; massi g/m2.

| **Table A** | **N** | **Age** | **Measurements** | **Field strength (T)** | **Sequence** | **Postprocessing approach** | **Subgroups** | | |
| --- | --- | --- | --- | --- | --- | --- | --- | --- | --- |
|  |  |  |  |  |  |  | Age | Sex | Ethnicity |
| Hudsmith[8] | 108 | 21-68 (38±12) | LV, RV, LA | 1.5 | SSFP | PMi | <35, ≥35 | Yes | No |
| Petersen[11] | 804 | 45-74 (59±7) | LV, RV, LA, RA | 1.5 | SSFP | PMe | 45-54, 55-64, 65-74 | Yes | No |
| Chuang[12] | 685 | 62±9 | LV | 1.5 | SSFP | PMe | <50, 50-59, 60-69, ≥70 | Yes | No |
| Maceira[13] | 120 | 20-80 | LV | 1.5 | SSFP | PMi | 20-80, 10y groups | Yes | No |
| Maceira[14] | 120 | 20-80 | RV | 1.5 | SSFP | PMi | 20-80, 10y groups | Yes | No |
| Lorenz[2] | 75 | 8-55 (28±9) | LV, RV | 1.5 | FGE | PMi | No | Yes | No |
| Alfakih[15] | 60 | 20-65 (43±12) | LV, RV | 1.5 | SSFP, FGE | PMi | <40, ≥40 | Yes | No |
| Natori[16] | 800 | 45-84 | LV | 1.5 | FGE | PMi | No | Yes | Yes |
| Kawut[17] | 4123 | 61.5±10.1 | RV | 1.5 | FGE | PMi | No | Yes | Yes |

| **Table B** | **Hudsmith[8]** | | **Petersen[11]** | | **Chuang[12]** | | **Maceira[13]** | | **Lorenz[2]** | | **Alfakih[15]** | | **Natori[16]** | |
| --- | --- | --- | --- | --- | --- | --- | --- | --- | --- | --- | --- | --- | --- | --- |
| **Sequence** | SSFP | | SSFP | | SSFP | | SSFP | | FGE | | SSFP | | FGE | |
|  | M | F | M | F | M | F | M | F | M | F | M | F | M | F |
| **LV-EDV** | 160 ±29 | 135 ±26 | 163 ±35 | 127±24 | 149 ±29 | 112 ±21 | 156 ±21 | 128 ±21 | 136 ±30 | 96±23 | 170±33 | 135 ±19 | 142±34 | 109±23 |
| **LV-EDVi** | 82±13 | 78±12 | 84±16 | 75±13 | 74±14 | 64±10 | 80±9 | 75±9 | 69±11 | 61±10 | 82±15 | 78±19 | 74±15 | 65 ±11 |
| **LV-ESV** | 50±16 | 42±12 | 68±17 | 49±12 | 44±14 | 31±9 | 53±11 | 42 ±9.5 | 45±14 | 32±9 | 61±16 | 49±11 | 47±20 | 31±10 |
| **LV-ESVi** | 25±8 | 24±6 | 35±8 | 29±7 | 22±7 | 18±5 | 37±6 | 24±5 |  |  |  |  | 25±9 | 18±5 |
| **LV-SV** | 112 ±19 | 91±17 | 94±23 | 78±16 | 105 ±20 | 81±15 | 104 ±14 | 86±14 | 92±21 | 65±16 | 108±21 | 86 ±12 | 95 ±21 | 78 ±17 |
| **LV-SVi** | 56±8 | 54±9 | 49±10 | 46±8 | 52±9 | 46±7 | 53 ±6 | 50 ±6.2 | 47±8 | 41±8 |  |  | 49±10 | 46 ±8 |
| **LV-EF** | 69±6 | 69±6 | 58±6 | 61±5 | 71±6 | 73±6 | 67±5 | 67±5 | 67±5 | 67±5 | 64±5 | 64±5 | 67 ±7 | 72 ±17 |
| **LV-Mass** | 123 ±21 | 96±27 | 102±23 | 70±13 | 99±21 | 58±13 | 146 ±20 | 108 ±18 | 178 ±31 | 125 ±26 | 133 ±24 | 90±12 | 164±36 | 114±24 |
| **LV-Massi** | 63±9 | 55 ±12 | 52±10 | 41±7 | 49±9 | 33±6 | 74±9 | 63±8 | 91±11 | 79±8 | 65±10 | 52±7 | 85±15 | 67±11 |

| **Table C** | **Hudsmith[8]** | | **Petersen[11]** | | **Maceira[14]** | | **Lorenz[2]** | | **Alfakih[15]** | | **Kawut [17]** | |
| --- | --- | --- | --- | --- | --- | --- | --- | --- | --- | --- | --- | --- |
| **Sequence** | **SSFP** | | **SSFP** | | **SSFP** | | **FGE** | | **SSFP** | | **FGE** | |
|  | M | F | M | F | M | F | M | F | M | F | M | F |
| **RV-EDV** | 173 ±39 | 148 ±35 | 179±40 | 137±27 | 163 ±25 | 126 ±21 | 157 ±35 | 106 ±24 | 177±33 | 131±24 | 141±30 | 109±23 |
| **RV-EDVi** | 91±16 | 84±17 | 91±18 | 80±15 | 83±12 | 73±9 | 80±13 | 67±10 | 86±14 | 75 ±14 | 72 ±13 | 62 ±11 |
| **RV-ESV** | 69±22 | 56±18 | 84±25 | 60±17 | 57±15 | 43±13 | 63±20 | 40±14 | 79±16 | 52 ±10 | 45 ±14 | 30 ±10 |
| **RV-ESVi** | 36±10 | 32±10 | 43±12 | 35±10 | 29±7 | 25±7 |  |  |  |  | 23 ±7 | 17 ±5 |
| **RV-SV** | 104 ±21 | 90±19 | 97±21 | 77±15 | 106 ±17 | 83±13 | 95±22 | 66±16 | 98 ±19 | 78 ±17 | 96±21 | 79 ±17 |
| **RV-SVi** | 55±9 | 53±9 | 49±10 | 45±8 | 54±8 | 48±6 | 48±8 | 42±8 |  |  | 49 ±10 | 45 ±8 |
| **RV-EF** | 61±6 | 63±5 | 54±7 | 57±6 | 66±6 | 66±6 | 60±7 | 63±8 | 55.1 ±3.7 | 59.8 ±5 | 68 ±6 | 73±6 |
| **RV-Mass** | 38±8 | 35±7 |  |  | 66±14 | 48±11 |  |  |  |  | 23±4 | 19±4 |
| **RV-Massi** | 20±4 | 20 ±4 |  |  | 34±7 | 28±5 |  |  |  |  | 12±2 | 11±2 |

**Table 3i.4: Outcomes studies against clinical endpoints for LV (Table A) and RV (Table B) function, mass or volumes.**

Hazard ratios (HR) with 95% confidence intervals (95%CI) for adjusted/multivariate predictive associations with outcome endpoints. Absolute values are shown as mean±SD.

EDV: end-diastolic volume. LV M/V ratio: LV mass/volume ratio. CAD: coronary artery disease. HF: heart failure.

PAH: pulmonary artery hypertension. EF: ejection fraction. SV: systolic volume. PVR: pulmonary vascular resistance. ESV: end-systolic volume. DCM: dilated cardiomyopathy. HTx: cardiac transplantation.

| **Table A** | **N** | **Type** | **Population** | **Follow-up**  **(months)** | **CMR biomarker** | **Outcome-endpoint** | | |
| --- | --- | --- | --- | --- | --- | --- | --- | --- |
| **Bluemke[18]** | 5098 | Observational  Prospective | Healthy volunteers | 48 | LV-EDV,  LV-mass  LV-M/V ratio | **LV Mass/Volume ratio** | | |
|  |  |  |  |  |  | CAD | HR 2.1 (1.1-4.1) | 0.02 |
|  |  |  |  |  |  | Stroke | HR 4.2 (1.5-11.2) | 0.0005 |
|  |  |  |  |  |  | **LV-EDV (per 10%)** | | |
|  |  |  |  |  |  | CAD | HR 0.9 (0.8-1) | 0.09 |
|  |  |  |  |  |  | HF | HR 1.3 (1.2-1.5) | <0.0001 |
|  |  |  |  |  |  | **LV mass** | | |
|  |  |  |  |  |  | Stroke | HR 1.2 (1-1.4) | 0.01 |
|  |  |  |  |  |  | HF | HR 1.4 (1.2-1.5) | <0.0001 |
| **Jain[19]** | 4965 | Observational  Prospective | Healthy volunteers | 70 | LV mass  LV M/V ratio | **LV mass** | | |
|  |  |  |  |  |  | Stroke | HR 1.3 (1.1-1.7) | ≤0.01 |
|  |  |  |  |  |  | HF | HR 1.8 (1.6-2.1) | ≤0.001 |
|  |  |  |  |  |  | **LV Mass/Volume ratio** | | |
|  |  |  |  |  |  | Stroke | HR 1.3 (1.1-1.6) | ≤0.01 |

| **Table B** | **N** | **Type** | **Population** | **Follow-up (months)** | **CMR biomarker** | **Outcome-endpoint** | | |
| --- | --- | --- | --- | --- | --- | --- | --- | --- |
| **Kawut[20]** | 4144 | Observational  Prospective | Healthy volunteers | 70 | RV- mass | HF/CV death | HR 2.52 (1.55-4.1) | <0.001 |
| **Van Wolferen[21]** | 64 | Observational  Prospective | PAH | 32 | RV-EDV RV-EF | Baseline biomarker ~ all cause-mortality | | |
|  |  |  |  |  |  | RVEDV | HR 1.6 | <0.001 |
|  |  |  |  |  |  | LVEDV | HR 0.7 | 0.002 |
|  |  |  |  |  |  | SV | HR 0.76 | <0.001 |
|  |  |  |  |  |  | Biomarker change at 1Y ~ all cause-mortality | | |
|  |  |  |  |  |  | RV-EDV | HR 1.05 | 0.036 |
|  |  |  |  |  |  | LV-EDV | HR 0.91 | 0.023 |
|  |  |  |  |  |  | LV-SV | HR 0.89 | 0.012 |
| **Van de Veerdonk[22]** | 110 | Observational  Prospective | PAH | 50 | RV-EF  PVR | Baseline biomarker ~ all cause-mortality | | |
|  |  |  |  |  |  | RV-EF | HR 0.92 (0.88-0.96) | <0.001 |
|  |  |  |  |  |  | PVR | HR 1.001 (1.001-1.002) | 0.002 |
|  |  |  |  |  |  | Biomarker change at 1Y ~ all cause-mortality | | |
|  |  |  |  |  |  | RV-EF | HR 0.93(0.88-0.99) | 0.026 |
| **Swift[23]** | 80 | Observational  Prospective | PAH | 32 | RV-EDV  RV-ESV  RV-EF | Baseline biomarker ~ all cause-mortality | | |
|  |  |  |  |  |  | RV-ESV | HR 1.55 (1.15-2.1) | 0.004 |
|  |  |  |  |  |  | RV-EDV | HR 1.3 (0.97-1.8) | 0.078 |
|  |  |  |  |  |  | RV-EF | HR 0.78 (0.5-1.14) | 0.187 |
| **Gulati[24]** | 250 | Observational  Prospective | DCM | 82 | RV-EF | RV-EF≤45% ~ adverse outcomes | | |
|  |  |  |  |  |  | Death/ HTx | HR 3.9 (2.16-7.04) | <0.001 |
|  |  |  |  |  |  | CVdeath/ HTx | HR 3.35 (1.76-6.39) | <0.001 |
|  |  |  |  |  |  | HF death/ admission/ HTx | HR 2.7 (1.32-5.51) | 0.006 |

**Table 3ii.1: Validation studies and comparative studies of strain imaging.**

Values are shown as MD ± SD (CoV, when available) between the CMR method of study and its reference. Strain measures are expressed as %. Coefficient of correlation (R) is provided when analysed. * Only CoV available.

SPAMM: spatial modulation of magnetization. λ1 and λ2: principal strains (most positive and most negative strains). DENSE: displacement encoding with stimulated echoes. GCS: global circumferential strain. GRS: global radial strain. DMD: Duchenne muscular dystrophy. HARP: harmonic phase image analysis. FT: feature tracking. LBBB: left bundle branch block. HCM: hypertrophic cardiomyopathy. GLS: global longitudinal strain. PAH: pulmonary artery hypertension. SENC: strain-encoded MR imaging. CP: constrictive pericarditis. RCM: restrictive cardiomyopathy.

|  | **Reference model** | **Reference method** | **N** | **CMR sequence** | **Parameter** | **Correlation** | | |
| --- | --- | --- | --- | --- | --- | --- | --- | --- |
| **Phantoms/animals** | | | | | | | | |
| **Young[25]** | Deformable silicone gel phantom | Analytical value |  | SPAMM | Axial shear strain | Homogeneous | λ1 0.122 | |
|  |  |  |  |  |  |  | λ2 0.036 | |
|  |  |  |  |  |  | Non-homogeneous | λ1 0.05 | |
|  |  |  |  |  |  |  | λ2 0.012 | |
| **Young[26]** | Deformable silicone gel phantom | Analytical value |  | SPAMM  DENSE | Shear strain  Radial strain | SPAMM | | |
|  |  |  |  |  |  | Shear | 1.4 ± 4.0 | |
|  |  |  |  |  |  | Radial | 0.0 ± 4.9 | |
|  |  |  |  |  |  | DENSE | | |
|  |  |  |  |  |  | Shear | 1.3 ± 2.1 | |
|  |  |  |  |  |  | Radial | 1.2 ± 4.8 | |
| **Yeon[27]** | Canine coronary artery ligation model | Sonomicrometry | 19 | SPAMM | Circumferential shortening | R=0.84 p<0.0001 | | |
|  |  |  |  |  |  | Effective identification of ischaemic/remote myocardium (2±3 vs 11±10, p 0.014) | | |
| **Compared to tagging** | | | | | | | | |
| **Young [26]** | Healthy volunteers | SPAMM | 19 | DENSE | GCS  GRS | GCS | 1.2± 3.9 (CoV 20.6) | <0.05 |
|  |  |  |  |  |  | GRS | 2.3 ± 14.0 (CoV 39.5) | NS |
| **Hor[28]** | DMD/ Healthy volunteers | HARP | 230 | FT | GCS | -0.36±1.67 (CoV 12.4)  R=0.899, p<0.0001 | | |
| **Wu[29]** | Healthy volunteers/ LBBB/ HCM | HARP | 30 | FT | GCS | Significant differences between tagging and FT | | |
|  |  |  |  |  |  | FT_endocardial_ | −23.8 ± 9.9 vs -13.4 ± 3.3 | <0.001 |
|  |  |  |  |  |  | FT_midwall_ | −16.4 ± 6.1 vs -13.4 ± 3.3 | 0.001 |
| **Augustine****[30]** | Healthy volunteers | HARP | 20 | FT | GCS  GLS  GRS | Only GCS showed reasonable agreement | | |
|  |  |  |  |  |  | GCS | -0.7 ± 2 | |
|  |  |  |  |  |  | GLS | -1 ± 7.5 | |
|  |  |  |  |  |  | GRS | 11 ± 6 | |
| **Ohyama[31]** | PAH/ healthy volunteers | HARP (GCS)  SENC (GLS) | 45 | FT | GLS  GLS RV  GCS | GLS | 2.8 ± 2.3 (CoV 13.3) | <0.001 |
|  |  |  |  |  |  |  | R=0.67 | <0.001 |
|  |  |  |  |  |  | GLS RV | 0.4 ± 3.2 (CoV 16.9) | 0.463 |
|  |  |  |  |  |  |  | R=0.71 | <0.001 |
|  |  |  |  |  |  | GCS | -2.8 ± 3.5 (CoV 22) | <0.001 |
|  |  |  |  |  |  |  | R=0.58 | <0.001 |
| **Compared to echo** | | | | | | | | |
| **Amaki[32]** | CP/RCM | Echo | 92 | FT | GLS | 0.7 ± 3.6  R=0.68, p<0.001 | | |
| **Kempny****[33]*** | Tetralogy of Fallot | Echo | 53 | FT | GLS  GCS  GRS  GLS RV | GLS | CoV 15.8 | |
|  |  |  |  |  |  | GCS | CoV 17.0 | |
|  |  |  |  |  |  | GRS | CoV 69 | |
|  |  |  |  |  |  | GLS RV | CoV 16.6 | |
| **Padiyath****[34]** | Tetralogy of Fallot | Echo | 20 | FT | GLS  GCS  GRS  GLS RV | GLS | -1.38 ± 4.59 (CoV 26.8) | |
|  |  |  |  |  |  | GCS | 0.77 ± 3.39 (CoV 15.5) | |
|  |  |  |  |  |  | GRS | -10.88 ± 22.23 (CoV 70.0) | |
|  |  |  |  |  |  | GLS RV | 0.05 ± 4.34 (CoV 28.8) | |
| **Onishi[35]** | Evaluation of LV function | Echo | 72 | FT | Radial dysynchrony | -0.3 ± 41.8  R=0.93, p<0.0001  Best agreement in patients with marked dysynchrony | | |

**Table 3ii.2. Reproducibility values reported for main strain measurements.**

Variability is reported as MD±SD(CoV) (CoV: derived from SD of the measurements divided by the mean value, expressed as %). When not available, we report MD±SD (Kutty and Padiyath), or intraclass correlation (ICC) (Castillo, Rosen and Yoneyama). **^*^**All values reported for healthy volunteers except Padiyath, who included Tetralogy of Fallot patients. FT: feature tracking. HARP: harmonic phase image analysis. SPAMM: spatial modulation of magnetization. C-SPAMM: complementary spatial modulation of magnetization. GCS: global circumferential strain. GLS: global longitudinal strain. GRS: global radial strain.

|  | **Augustine [30]** | **Kempny [33]** | **Morton** **[36]** | **Kutty** **[37]** | **Padiyath [34]*** | **Castillo [38]** | **Rosen** **[39]** | **Yoneyama** **[40]** | **Swoboda [41]** |
| --- | --- | --- | --- | --- | --- | --- | --- | --- | --- |
| **Sequence** | FT | FT | FT | FT | FT | Tagging (HARP) | Tagging (HARP) | Tagging (SPAMM) | Tagging  (C-SPAMM) |
| **N** | 12 | 25 | 16 | 15 | 10 | 24 | 24 | 30 | 12 |
| **Interstudy** | | | | | | | | |  |
| GCS |  |  | 1.0±3.5 (20.3) |  |  |  |  |  | (3.7-5.5) |
| GLS |  |  | -1.1±5.4 (26.4) |  |  |  |  |  |  |
| GRS |  |  | -3.2±5.7 (27.2) |  |  |  |  |  | (13.8-23.4) |
| LV torsion |  |  |  |  |  |  |  |  | (9.8-12.2) |
| **Interobserver** | | | | | | | | |  |
| GCS | 4.9 | 8.5 |  | -0.01±1.7 (7.1) | -0.19±1.25 (5.8) | 0.84 | 0.81 |  | (3.5-6.2) |
| GLS | 10.9 | 9.6 |  | -0.32±1.1 (5.5) | -2.23±3.2 (17.9) |  |  |  |  |
| GRS | 32.3 | 21.4 |  | -1.57±5.5 (11.0) | -3.21±8.5 (32.3) | 0.71 |  |  | (11.8-21.8) |
| LV torsion |  |  |  |  |  |  |  | 0.94 | (3.5-7.2) |
| **Intraobserver** | | | | | | | | |  |
| GCS | 2.8 | 6.7 |  |  | -0.19 | 0.89 | 0.84 |  | (1.5-4.3) |
| GLS | 12.3 | 10.8 |  |  | -2.23 |  |  |  |  |
| GRS | 22.9 | 21.4 |  |  | -3.21 | 0.77 |  |  | (10.6-14.8) |
| LV torsion |  |  |  |  |  |  |  | 0.91 | (1.2-4.4) |

**Table 3ii.3: Normal values reported by different studies for main strain measurements.**

Venkatesh provided normality values for strain according to segment, age, sex and ethnicity. Augustine for sex and segments, and Moore [42] and Del-Canto for each myocardial segment. GCS, GLS and GRS are expressed as %, SRe s^-1^ and LV torsion º or º/cm.

SPAMM: spatial modulation of magnetization. HARP: harmonic phase image analysis. FT: feature tracking. GCS: global circumferential strain. GLS: global longitudinal strain. GRS: global radial strain. SR_E_: early diastolic strain rate. MESA: multi-ethnic study of atherosclerosis, includes volunteers without previous history of cardiovascular disease.

|  | **Edvardsen** **[43]** | **Donekal** **[44]** | **Yoneyama [40]** | **Venkatesh [45]** | **Del-Canto [46]** | **Nelson [47]** | **Augustine [30]** | **Kempny [33]** | **Kutty [37]** | **Padiyath [34]** | **Morton [36]** |
| --- | --- | --- | --- | --- | --- | --- | --- | --- | --- | --- | --- |
| **Sequence** | Tagging (SPAMM) | Tagging (SPAMM) | Tagging (SPAMM) | Tagging (SPAMM) | Tagging (SPAMM) | Tagging (HARP) | FT | FT | FT | FT | FT |
| **N** | 188 | 1116 | 1478 | 129 | 39 | 15 | 145 | 25 | 20 | 20 | 16 |
| **Population** | MESA | MESA | MESA | MESA low-risk | Low Risk | Healthy volunteers | Healthy volunteers | Healthy volunteers | Healthy volunteers | Healthy volunteers | Healthy volunteers |
| GCS | -17.1±5 | -15.5±3.2 |  | -18±2.2 | -20.3±3 | −21.9±0.5 | −21 ± 0.03 | -22.0±3.9 | -24.6±2.4 | -24.6±2.5 | -17.4±4.6 |
| GLS |  |  |  |  |  |  | −19 ± 0.03 | -21.3±3.3 | -20.0±5.1 | -19.9±5.1 | -20±5.2 |
| GRS |  |  |  | 25.8±8.4 | 12.1±4.4 |  | 25 ± 0.06 | 28.0 ± 11.3 | 50 ± 12.4 | 50.9±12.4 | 20.8±6.6 |
| SR_E_ | 2.2±1.1 | 1.04±0.4 |  |  |  |  |  |  |  |  |  |
| Torsion |  | 8.7±2.04º | 3.9±1.3º/cm  F: 4.2±1.3  M: 3.5±1.1 | 3.61±1.15 º/cm |  |  | 15.52 ± 7.55 º |  |  |  |  |

**Table 3ii.4 Outcome studies for RWMA and strain parameters**

Hazard ratios (HR) with 95% confidence intervals (95%CI) for adjusted/multivariate predictive associations with outcome endpoints. Chi-square test (χ2) was used to assess the predictive value of biomarkers.

HARP: harmonic phase image analysis. MESA: multi-ethnic study of atherosclerosis. SRI: strain relaxation index. HF: heart failure. AF: atrial fibrillation. SPAMM: spatial modulation of magnetization. DSMR: dobutamine stress MR. WMA: wall motion abnormality. CAD: coronary artery disease. LGE: late gadolinium enhancement. RWMI: regional wall motion index. MI: myocardial infarction. CR: contractile reserve. SENC: strain-encoded MR imaging. FT: feature tracking. DCM: dilated cardiomyopathy. GLS: global longitudinal strain.

|  | **N** | **Sequence** | **Population** | **Follow-up (months)** | **CMR biomarker** | **Outcome-endpoint** | | |
| --- | --- | --- | --- | --- | --- | --- | --- | --- |
| **Ambale-Venkatesh [48]** | 1544 | Tagging (HARP) | Healthy population (MESA) | 96 | SRI | HF | HR 2.25 (1.3-3.89) | 0.039 |
|  |  |  |  |  |  | HF/AF | HR 1.88 (1.29-2.74) | 0.099 |
| **Kuijpers [49]** | 211 | Tagging (SPAMM)  DSMR | Chest pain | 17.3 | WMA with tagging | Improved detection of WMA with tagging (27% vs 32%, p 0.002).  Survival (17 months) if CMR – 98.2%. | | |
| **Kelle [50]** | 177 | DSMR | CAD and scar in LGE | 20.3 | RWMI | Infarct size was the only predictor of events (death+MI), HR 1.303 (1.026–1.655), p 0.03 | | |
|  |  |  |  |  |  | In high-risk patients (LGE in >6 segments), CR+ was the only predictor of events | | |
|  |  |  |  |  |  | CR- | χ^2^=0.7 |  |
|  |  |  |  |  |  | CR+ | χ^2^=4.0 | <0.001 |
| **Korosoglou [51]** | 320 | SENC  DSMR | CAD | 28 | WMA  SENC | WMA and SENC improved the prediction of cardiac death+MI compared with clinical info | | |
|  |  |  |  |  |  | Clinical | χ^2^=13.0 |  |
|  |  |  |  |  |  | Rest WMA | χ^2^=26.1 | <0.001 |
|  |  |  |  |  |  | Stress WMA | χ2=39.3 | <0.001 |
|  |  |  |  |  |  | SENC | χ2=50.7 | <0.001 |
| **Buss [52]** | 210 | FT | DCM | 63.6 | GLS | GLS >-12.5% was associated with CV events | | |
|  |  |  |  |  |  | GLS | HR 1.27 (1.06-1.52) | <0.02 |

Fieno DS, Jaffe WC, Simonetti OP, Judd RM, Finn JP. TrueFISP: assessment of accuracy for measurement of left ventricular mass in an animal model. J Magn Reson Imaging. 2002;15:526-31.

Lorenz CH, Walker ES, Morgan VL, Klein SS, Graham TP. Normal human right and left ventricular mass, systolic function, and gender differences by cine magnetic resonance imaging. J Cardiovasc Magn Reson. 1999;1:7-21.

Childs H, Ma L, Ma M, Clarke J, Cocker M, Green J, et al. Comparison of long and short axis quantification of left ventricular volume parameters by cardiovascular magnetic resonance, with ex-vivo validation. J Cardiovasc Magn Reson. 2011;13:40.

Codella NC, Lee HY, Fieno DS, Chen DW, Hurtado-Rua S, Kochar M, et al. Improved left ventricular mass quantification with partial voxel interpolation: in vivo and necropsy validation of a novel cardiac MRI segmentation algorithm. Circ Cardiovasc Imaging. 2012;5:137-46.

Gilbert K, Lam HI, Pontré B, Cowan BR, Occleshaw CJ, Liu JY, et al. An interactive tool for rapid biventricular analysis of congenital heart disease. Clin Physiol Funct Imaging. 2017;37:414-420.

Farber NJ, Reddy ST, Doyle M, Rayarao G, Thompson DV, Olson P, et al. Ex vivo cardiovascular magnetic resonance measurements of right and left ventricular mass compared with direct mass measurement in excised hearts after transplantation: a first human SSFP comparison. J Cardiovasc Magn Reson. 2014;16:74.

Lin HY, Freed D, Lee TW, Arora RC, Ali A, Almoustadi W, et al. Quantitative assessment of cardiac output and left ventricular function by noninvasive phase-contrast and cine MRI: validation study with invasive pressure-volume loop analysis in a swine model. J Magn Reson Imaging. 2011;34:203-10.

Hudsmith LE, Petersen SE, Francis JM, Robson MD, Neubauer S. Normal human left and right ventricular and left atrial dimensions using steady state free precession magnetic resonance imaging. J Cardiovasc Magn Reson. 2005;7:775-82.

Grothues F, Smith GC, Moon JC, Bellenger NG, Collins P, Klein HU, et al. Comparison of interstudy reproducibility of cardiovascular magnetic resonance with two-dimensional echocardiography in normal subjects and in patients with heart failure or left ventricular hypertrophy. Am J Cardiol. 2002;90:29-34.

Grothues F, Moon JC, Bellenger NG, Smith GS, Klein HU, Pennell DJ. Interstudy reproducibility of right ventricular volumes, function, and mass with cardiovascular magnetic resonance. Am Heart J. 2004;147:218-23.

Petersen SE, Aung N, Sanghvi MM, Zemrak F, Fung K, Paiva JM, et al. Reference ranges for cardiac structure and function using cardiovascular magnetic resonance (CMR) in Caucasians from the UK Biobank population cohort. J Cardiovasc Magn Reson. 2017;19:18.

Chuang ML, Gona P, Hautvast GL, Salton CJ, Breeuwer M, O'Donnell CJ, et al. CMR reference values for left ventricular volumes, mass, and ejection fraction using computer-aided analysis: The Framingham Heart Study. J Magn Reson Imaging. 2014;39:895-900.

Maceira AM, Prasad SK, Khan M, Pennell DJ. Normalized left ventricular systolic and diastolic function by steady state free precession cardiovascular magnetic resonance. J Cardiovasc Magn Reson. 2006;8:417-26.

Maceira AM, Prasad SK, Khan M, Pennell DJ. Reference right ventricular systolic and diastolic function normalized to age, gender and body surface area from steady-state free precession cardiovascular magnetic resonance. Eur Heart J. 2006;27:2879-88.

Alfakih K, Plein S, Thiele H, Jones T, Ridgway JP, Sivananthan MU. Normal human left and right ventricular dimensions for MRI as assessed by turbo gradient echo and steady-state free precession imaging sequences. J Magn Reson Imaging. 2003;17:323-9.

Natori S, Lai S, Finn JP, Gomes AS, Hundley WG, Jerosch-Herold M, et al. Cardiovascular function in multi-ethnic study of atherosclerosis: normal values by age, sex, and ethnicity. AJR Am J Roentgenol. 2006;186(Suppl 2):S357-65.

Kawut SM, Lima JA, Barr RG, Chahal H, Jain A, Tandri H, et al. Sex and race differences in right ventricular structure and function: the multi-ethnic study of atherosclerosis-right ventricle study. Circulation. 2011;123:2542-51.

Bluemke DA, Kronmal RA, Lima JA, Liu K, Olson J, Burke GL, et al. The relationship of left ventricular mass and geometry to incident cardiovascular events: the MESA (Multi-Ethnic Study of Atherosclerosis) study. J Am Coll Cardiol. 2008;52:2148-55.

Jain A, McClelland RL, Polak JF, Shea S, Burke GL, Bild DE, et al: Cardiovascular imaging for assessing cardiovascular risk in asymptomatic men versus women: the multi-ethnic study of atherosclerosis (MESA). Circ Cardiovasc Imaging. 2011;4:8-15.

Kawut SM, Barr RG, Lima JA, Praestgaard A, Johnson WC, Chahal H, et al. Right ventricular structure is associated with the risk of heart failure and cardiovascular death: The Multi-Ethnic Study of Atherosclerosis (MESA)--right ventricle study. Circulation. 2012;126:1681-8.

Van Wolferen SA, Marcus JT, Boonstra A, Marques KM, Bronzwaer JG, Spreeuwenberg MD, et al. Prognostic value of right ventricular mass, volume, and function in idiopathic pulmonary arterial hypertension. Eur Heart J. 2007;28:1250-7.

Van de Veerdonk MC, Kind T, Marcus JT, Mauritz GJ, Heymans MW, Bogaard HJ, et al. Progressive right ventricular dysfunction in patients with pulmonary arterial hypertension responding to therapy. J Am Coll Cardiol. 2011;58:2511-9.

Swift AJ, Rajaram S, Campbell MJ, Hurdman J, Thomas S, Capener D, et al. Prognostic value of cardiovascular magnetic resonance imaging measurements corrected for age and sex in idiopathic pulmonary arterial hypertension. Circ Cardiovasc Imaging. 2014;7:100-106.

Gulati A, Ismail TF, Jabbour A, Alpendurada F, Guha K, Ismail NA, et al. The prevalence and prognostic significance of right ventricular systolic dysfunction in nonischemic dilated cardiomyopathy. Circulation. 2013;128:1623-33.

Young AA, Axel L, Dougherty L, Bogen DK, Parenteau CS. Validation of tagging with MR imaging to estimate material deformation. Radiology. 1993;188:101–8.

Young AA, Li B, Kirton RS, Cowan BR. Generalized spatiotemporal myocardial strain analysis for DENSE and SPAMM imaging. Magn Reson Med. 2012;67:1590-9.

Yeon SB, Reichek N, Tallant BA, Lima JA, Calhoun LP, Clark NR, et al. Validation of in vivo myocardial strain measurement by magnetic resonance tagging with sonomicrometry. J Am Coll Cardiol. 2001;38:555-61.

Hor KN, Gottliebson WM, Carson C, Wash E, Cnota J, Fleck R, et al. Comparison of Magnetic Resonance Feature Tracking for Strain Calculation With Harmonic Phase Imaging Analysis. JACC Cardiovasc Imaging. 2010;3:144–51.

Wu L, Germans T, Güçlü A, Heymans MW, Allaart CP, van Rossum AC. Feature tracking compared with tissue tagging measurements of segmental strain by cardiovascular magnetic resonance. J Cardiovasc Magn Reson. 2014;16:10.

Augustine D, Lewandowski AJ, Lazdam M, Rai A, Francis J, Myerson S, et al. Global and regional left ventricular myocardial deformation measures by magnetic resonance feature tracking in healthy volunteers: comparison with tagging and relevance of gender. J Cardiovasc Magn Reson. 2013;15:8.

Ohyama Y, Ambale-Venkatesh B, Chamera E, Shehata ML, Corona-Villalobos CP, Zimmerman SL, et al. Comparison of strain measurement from multimodality tissue tracking with strain-encoding MRI and harmonic phase MRI in pulmonary hypertension. Int J Cardiol. 2015;182:342–8.

Amaki M, Savino J, Ain DL, Sanz J, Pedrizzetti G, Kulkarni H, et al. Diagnostic concordance of echocardiography and cardiac magnetic resonance-based tissue tracking for differentiating constrictive pericarditis from restrictive cardiomyopathy. Circ Cardiovasc Imaging. 2014;7:819-27.

Kempny A, Fernández-Jiménez R, Orwat S, Schuler P, Bunck AC, Maintz D, et al. Quantification of biventricular myocardial function using cardiac magnetic resonance feature tracking, endocardial border delineation and echocardiographic speckle tracking in patients with repaired tetralogy of Fallot and healthy controls. J Cardiovasc Magn Reson. 2012;14:32.

Padiyath A, Gribben P, Abraham JR, Li L, Rangamani S, Schuster A, et al. Echocardiography and cardiac magnetic resonance based feature tracking in the assessment of myocardial mechanics in tetralogy of Fallot: an intermodality comparison. Echocardiography. 2013;30:203–10.

Onishi T, Saha SK, Ludwig DR, Onishi T, Marek JJ, Cavalcante JL, et al. Feature tracking measurement of dyssynchrony from cardiovascular magnetic resonance cine acquisitions: comparison with echocardiographic speckle tracking. J Cardiovasc Magn Reson. 2013;15:95.

Morton G, Schuster A, Jogiya R, Kutty S, Beerbaum P, Nagel E. Inter-study reproducibility of cardiovascular magnetic resonance myocardial feature tracking. J Cardiovasc Magn Reson. 2012;14:43.

Kutty S, Rangamani S, Venkataraman J, Li L, Rangamani S, Schuster A, et al. Reduced global longitudinal and radial strain with normal left ventricular ejection fraction late after effective repair of aortic coarctation: a CMR feature tracking study. Int J Cardiovasc Imaging. 2013;29:141–50.

Castillo E, Osman NF, Rosen BD, El-Shehaby I, Pan L, Jerosch-Herold M, et al. Quantitative assessment of regional myocardial function with MR-tagging in a multi-center study: interobserver and intraobserver agreement of fast strain analysis with Harmonic Phase (HARP) MRI. J Cardiovasc Magn Reson. 2005;7:783-91.

Rosen BD, Edvardsen T, Lai S, Castillo E, Pan L, Jerosch-Herold M, et al. Left ventricular concentric remodeling is associated with decreased global and regional systolic function: the Multi-Ethnic Study of Atherosclerosis. Circulation. 2005;112:984-91.

Yoneyama K, Gjesdal O, Choi EY, Wu CO, Hundley WG, Gomes AS, et al. Age, sex, and hypertension-related remodeling influences left ventricular torsion assessed by tagged cardiac magnetic resonance in asymptomatic individuals: the multi-ethnic study of atherosclerosis. Circulation. 2012;126:2481-90.

Swoboda PP, Larghat A, Zaman A, Fairbairn TA, Motwani M, Greenwood JP, et al. Reproducibility of myocardial strain and left ventricular twist measured using complementary spatial modulation of magnetization. J Magn Reson Imaging. 2014 ;39:887-94.

Moore CC, Lugo-Olivieri CH, McVeigh ER, Zerhouni EA. Three-dimensional systolic strain patterns in the normal human left ventricle: characterization with tagged MR imaging. Radiology. 2000;214:453–66.

Edvardsen T, Rosen BD, Pan L, Jerosch-Herold M, Lai S, Hundley WG, et al. Regional diastolic dysfunction in individuals with left ventricular hypertrophy measured by tagged magnetic resonance imaging--the Multi-Ethnic Study of Atherosclerosis (MESA). Am Heart J. 2006;151:109-114.

Donekal S, Venkatesh BA, Liu YC, Liu CY, Yoneyama K, Wu CO, et al. Interstitial Fibrosis, Left Ventricular Remodeling, and Myocardial Mechanical Behavior in a Population-Based Multiethnic Cohort: The Multi-Ethnic Study of Atherosclerosis (MESA) Study. Circ Cardiovasc Imaging. 2014;7:292-302.

Venkatesh BA, Donekal S, Yoneyama K, Wu C, Fernandes VR, Rosen BD, et al. Regional myocardial functional patterns: Quantitative tagged magnetic resonance imaging in an adult population free of cardiovascular risk factors: The multi-ethnic study of atherosclerosis (MESA). J Magn Reson Imaging. 2015;42:153-9.

Del-Canto I, Lopez-Lereu MP, Monmeneu JV, Croisille P, Clarysse P, Chorro FJ, et al. Characterization of normal regional myocardial function by MRI cardiac tagging. J Magn Reson Imaging. 2015;41:83–92.

Nelson MD, Szczepaniak LS, Wei J, Haftabaradaren A, Bharadwaj M, Sharif B, et al. Diastolic dysfunction in women with signs and symptoms of ischemia in the absence of obstructive coronary artery disease: a hypothesis-generating study. Circ Cardiovasc Imaging. 2014;7:510-6.

Ambale-Venkatesh B, Armstrong AC, Liu CY, Donekal S, Yoneyama K, Wu CO, et al. Diastolic function assessed from tagged MRI predicts heart failure and atrial fibrillation over an 8-year follow-up period: the multi-ethnic study of atherosclerosis. Eur Heart J Cardiovasc Imaging. 2014;15:442-9.

Kuijpers D, Ho KY, van Dijkman PR, Vliegenthart R, Oudkerk M. Dobutamine cardiovascular magnetic resonance for the detection of myocardial ischemia with the use of myocardial tagging. Circulation. 2003;107:1592-7.

Kelle S, Roes SD, Klein C, Kokocinski T, de Roos A, Fleck E, et al. Prognostic Value of Myocardial Infarct Size and Contractile Reserve Using Magnetic Resonance Imaging. J Am Coll Cardiol. 2009;54:1770–7.

Korosoglou G, Gitsioudis G, Voss A, Lehrke S, Riedle N, Buss SJ, et al. Strain-Encoded Cardiac Magnetic Resonance During High-Dose Dobutamine Stress Testing for the Estimation of Cardiac Outcomes. J Am Coll Cardiol. 2011;58:1140–9.

Buss SJ, Breuninger K, Lehrke S, Voss A, Galuschky C, Lossnitzer D, et al. Assessment of myocardial deformation with cardiac magnetic resonance strain imaging improves risk stratification in patients with dilated cardiomyopathy. Eur Heart J Cardiovasc Imaging. 2015;16:307–15.
