# Supplementary material for: Society for Cardiovascular Magnetic Resonance (SCMR) expert consensus for CMR imaging endpoints in clinical research: part I - analytical validation and clinical qualification
Source: J Cardiovasc Magn Reson. 2018 Sep 20;20:67. doi: 10.1186/s12968-018-0484-5 (PMC6147157; doi:10.1186/s12968-018-0484-5)
Supplement: Supplementary file 2 — Late gadolinium enhancement. Table 3b-i.1: Validation studies with LGE. Table 3b-i.2: Reproducibility of measurements for LGE. Table 3b-i.3: Comparative studies with other imaging techniques in ischaemic heart disease. Table 3b-i.4: Outcome studies with LGE. (DOCX 79 kb) [file 12968_2018_484_MOESM2_ESM.docx]

## Late gadolinium enhancement

**Table 3b-i.1: Validation studies with LGE.** Agreement expressed as Pearson r-coefficient. ICM- ischemic cardiomyopathy, NICM –non-ischemic cardiomyopathy, LGE - late gadolinium enhancement, SD – standard deviation, FWHM - full-width half-maximum

| **Author** | **N** | **Disease model** | **Histological Staining** | **Time-points** | **Correlation** | | |
| --- | --- | --- | --- | --- | --- | --- | --- |
| **Histological validation** | | | |  | | | |
| **Animal studies** | | |  |  | **LGE method** | **R** | **P value** |
| **Kim[1]** | 9 | ICM (Dogs) | Hematoxylin and eosin and/or Masson’s trichrome | 1, 3 days and 8 weeks after the intervention | LGE >2SD | Day 1 (r= 0.99)  Day 3 (r= 0.99)  Week 8 (r= 0.97) | p< 0.001 |
| **Fieno[2]** | 24 | ICM (Dogs) | Triphenyltetrazolim chloride-stained | 4 h, 1 day, 3 days, 10 days, 4 weeks and 8 weeks after the intervention. | LGE >3SD | r=0.99 | p< 0.001 |
| **Wagner[3]** | 15 | ICM (Dogs) | Triphenyltetrazolim chloride-stained | 2 days after the  coronary artery occlusion/reperfusion | LGE >2 SD | r=0.98 | p≤0.05 |
| **Human studies** | | |  |  |  | | |
| **Gulati[4]** | 16 | NICM | Picrosirius red/ Qualitative assessment | median of 5.3 years | FWHM | Excellent correlation | NA |
| **Iles[5]** | 11 | NICM/ICM | Masson trichrome,  Picrosirius red/  2-10 SD | / | LGE > 6 SD | r=0.91 | p< 0.001 |

**Table 3b-i.2: Reproducibility of measurements for LGE.** Values are expressed as MD±SD and CoV in brackets when available. AMI - acute myocardial infarction, CMI -chronic myocardial infarction, HCM - hypertrophic cardiomyopathy, NICM - non-ischaemic cardiomyopathy, SD - standard deviation, FWHM: full-width half-maximum.

| **Author** | **Type of patients** | **N of patients** | **LGE definition** | **Interobserver** | | **Intraobserver** | **Interstudy** |
| --- | --- | --- | --- | --- | --- | --- | --- |
| **Thiele[6]** | AMI, CMI | 21 | Manual quantification | -0.7% ±2.2 (1.6%) | | 0.3% ±1.7 (2.8%) | -0.5% ±2.4 (2.4%) |
| **Desch[7]** | AMI | 20 | Manual quantification | (2.4%) | | (2.4%) | 0.1 ± 2.2 (11%) |
| **Flett [8]** | AMI, CMI and HCM | 60 (20+20+20) | Manual quantification, 2,3,4,5,6 SD and FWHM | For inter- and intraobserver FWHM was the most reproducible in all 3 conditions (interstudy reproducibility was not performed) | | | |
| **McAlindon [9]** | AMI | 40 | Manual quantification, 2-,3-,5- SD, Otsu and FWHM | Manual was the most reproducible followed by FWHM for myocardial scar and Otsu for myocardial oedema | | | |
| **Khan[10]** | AMI | 20 | Manual quantification, 5-8 SD, FWHM and Otsu | FWHM had lowest observer variability at 1.5T | | | |
| **Neilan[11]** | NICM | 15 | 2SD and FWHM | **2SD:** 0.8 | **2SD:** 1.1 | |  |
|  |  |  |  | **FWHM**: 0.5 | **FWHM**: 0.5 | |  |
| **Chan[12]** | HCM | 24 | 6SD | (6.3) | | (5.9) |  |

**Table 3b-i.3. Comparative studies with other imaging techniques in ischaemic heart disease.** CAD – coronary artery disease, AMI – acute myocardial infarction, CMI – chronic myocardial infarction, SPECT - single photon emission computed tomography, PET – positron emission tomography.

| **Validation against established imaging techniques** | | | | | | | | |
| --- | --- | --- | --- | --- | --- | --- | --- | --- |
| **Author** | **Disease model** | **N** | **Assessment** | **Study design** | **Outcome** | | |  |
| **Wagner[13]** | Suspected/known CAD | 91 | Visual | Prospective | | Associations | SPECT is systematically less sensitive for subendocardial scar compared to CMR and histology.  Rate of SPECT-detected infarcts as defined by CMR increases with transmurality:   \| CMR transmurality \| SPECT sensitivity \| \| --- \| --- \| \| 1-25% \| 50% \| \| 26-50% \| 57% \| \| 51-75% \| 77% \| \| 76-100% \| 100% \| |  |
| **Ibrahim[14]** | AMI | 78 | Visual | Prospective | | Comparisons | CMR is more sensitive than SPECT in detecting small MI, non-Q MI and non-anterior MI |  |
| **Wu[15]** | CMI | 116 (CMR vs SPECT)  46 (CMR vs PET) | Visual | Retrospective | | Correlations | Overall agreement of viability criteria between SPECT and CMR: 96.8 % (κ = 0.62). Agreement in dysfunctional segments: 86 % (κ = 0.52). |  |
|  |  |  |  |  |  |  | Overall agreement of viability criteria between PET and CMR: 92.7% (κ = 0.51) |  |

**Table 3b-i.4. Outcome studies with LGE.** Follow-up is expressed in months. HR and AUC are provided followed by 95% CI limits in brackets. Studies with n> 100 patients and hard CV endpoints qualified for inclusion. Absolute values are expressed as mean followed by SD. All analyses are multivariable/adjusted unless otherwise stated (†). § - Given the few events statistical comparisons were not performed.

STEMI – ST elevation MI, FWHM - full-width half-maximum, MACE – major adverse cardiovascular events, HR – hazard ratio, AUC – area under the curve, LGE – late gadolinium enhancement, LVEF – LV ejection fraction, MVO – microvascular obstruction, SPECT - Single Photon Emission Computed Tomography, MSI - myocardial salvage index, HF - heart failure, CAD - coronary artery disease, UA - unstable angina, VT – ventricular tachycardia, ICD - implantable cardioverter defibrillator, ICM - ischemic cardiomyopathy, NICM - non-ischemic cardiomyopathy, SCD - sudden cardiac death, AF – atrial fibrillation, CT - cardiac transplantation, HCM - hypertrophic cardiomyopathy, NSVT - non-sustained ventricular tachycardia, PM - pacemaker.

| **Author** | **N** | **Population** | **LGE Assessment method** | **Follow-up** | **Endpoints** | **CMR-outcomes** | | |
| --- | --- | --- | --- | --- | --- | --- | --- | --- |
| **Acute myocardial infarction** | | |  |  |  |  | | |
| **Larose[16]** | 103 | STEMI | FWHM | 33 | MACE | LGE present | HR 1.36(1.11-1.66) | 0.03 |
|  |  |  |  |  |  | LGE extent | AUC 0.92 (0.84-0.98) | <0.001 |
|  |  |  |  |  |  |  | HR 1.72 (1.43-2.01) for MACE | 0.007 |
| **Wu[17]** | 122 | STEMI | Manual | 18 | MACE | LGE extent was the strongest predictor for MACE | | |
|  |  |  |  |  |  | LGE extent | HR 1.06 (1-1.12) | 0.04 |
|  |  |  |  |  |  | LVEF | HR 0.96 (0.88-1.05) | 0.39 |
|  |  |  |  |  |  | LGE≥18.5% 🡪 sensitivity 88%, NPV 96% for MACE. Predictor of MACE (p=0.007) and LV adverse remodeling (p=0.004). | | |
| **Hadamitzky[18]** | 281 | STEMI | FWHM  2-, 3-, 4-, 5- and 6-SD | 36 | MACE | MVO was the strongest predictor for MACE | | |
|  |  |  |  |  |  | MVO | HR 1.17 (1.1-1.25) | <0.001 |
|  |  |  |  |  |  | LGE extent (CMR- 6SD) | HR 1.85 (1.21-2.81) | 0.0043 † |
|  |  |  |  |  |  | LGE extent (SPECT) | HR 2.02 (1.33-3.06) | <0.001 † |
| **Eitel[19]** | 738 | STEMI | 5SD | 12 | MACE | CMR parameters were predictive of 1-y MACE | | |
|  |  |  |  |  |  | LVEF≤47% | AUC 0.69 (0.66-0.73) | <0.001 |
|  |  |  |  |  |  |  | HR 4.38 (2.49-7.71) | <0.001 † |
|  |  |  |  |  |  | LGE extent ≥19% | AUC 0.72 (0.69-0.76) | <0.001 |
|  |  |  |  |  |  |  | HR 5.41 (2.78-10.5) | <0.001 † |
|  |  |  |  |  |  | MSI ≤35 | AUC 0.7 (0.66-0.74) | <0.001 |
|  |  |  |  |  |  | MVO ≥1.4% | AUC 0.73 (0.69-0.76) | <0.001 |
|  |  |  |  |  |  |  | HR 5.62 (3-12-10.1) | <0.01 † |
|  |  |  |  |  |  |  | HR 3.63 (1.35-7.9) | 0.004 |
| **Eitel[20]** | 208 | STEMI | 5- SD | 6 | MACE | CMR parameters were predictive of 6-m MACE | | |
|  |  |  |  |  |  | MVO | HR 1.1 (1.03-1.17) | 0.004 † |
|  |  |  |  |  |  | LGE extent | HR 1.08 (1.05-1.12) | <0.001 † |
|  |  |  |  |  |  | MSI | HR 0.95 (0.93-0.97) | <0.001 † |
|  |  |  |  |  |  |  | HR 0.93 (0.91-0.96) | <0.001 |
|  |  |  |  |  |  | HF hospitalization | HR 1.20 (1.19-1.21) | <0.0001 |
| **Stone**[21] | 1889 | STEMI | Metanalysis | 12 | Survival | All-cause mortality | HR 1.19 (1.18-1.20) | <0.0001 |
| **De Waha**[22] | 1688 | STEMI | Metanalysis | 6 | MACE | All-cause mortality | HR 1.14 (1.09–1.19) |  |
|  |  |  |  |  |  | HF hospitalization | HR 1.08 (1.05–1.12) |  |
| **Stable coronary artery disease** | | |  |  |  | HF hospitalization | HR 1.20 (1.19-1.21) | <0.0001 |
| Steel[23] | 254 | Suspected CAD | 2-SD | 17 | MACE | LGE absent 🡪 98.1% event-free survival | | |
|  |  |  |  |  |  | LGE present | | |
|  |  |  |  |  |  | CV death/MI | HR 5.31 (2.35-12) | <0.0001 |
|  |  |  |  |  |  | CV death/ MI/UA | HR 8.09 (3.9-16.8) | <0.0001 |
| Kwong[24] | 195 | Suspected CAD with no prior MI | 2-SD | 16 | MACE | LGE present >7-fold risk of events | | |
|  |  |  |  |  |  | CV death | HR 9.43 (3.15-28.3) | <0.0001 |
|  |  |  |  |  |  | MACE and VT and HF | HR 5.98 (2.68-13.3) | <0.0001 |
| **Mixed patient groups (heart failure, indication for ICD, etc)** | | | | |  |  |  |  |
| Iles[25] | 103 | ICD for primary prevention (NICM/ICM) | 2-SD | 19 | ICD shock | LGE+ | 21 (+) vs 0% (-) | 0.01 |
|  |  |  |  |  |  | No differences per aetiology (29% NICM vs 14% ICM, P=NS).  Similar LVEF in LGE+/- and ICD shock +/-. | | |
| Gao[26] | 124 | ICD for primary prevention (NICM/ ICM) | FWHM  2-, 3-, 5-SD, | 21 | ICD shock/ SCD | LGE mass predicts arrhythmic events. (events vs no events) | | |
|  |  |  |  |  |  | Total | 59±30 vs 32±19 g | 0.001 |
|  |  |  |  |  |  | NICM | 46±38 vs 23±15g | 0.003 |
|  |  |  |  |  |  | ICM | 69±17 vs 42±19g | 0.001 |
| Klem[27] | 137 | ICD for primary prevention (NICM/ ICM) | 3-SD | 24 | Death, ICD shock | Scar size (>5% LV mass) predicted adverse outcomes and improved risk stratification beyond LVEF. | | |
|  |  |  |  |  |  | Death | HR 8.75 (1.89-41) | 0.006 |
|  |  |  |  |  |  | ICD shock | HR 4.76 (1.65-13.7) | 0.004 |
|  |  |  |  |  |  | Death/ICD shock | HR 4.59 (1.79-11.8) | 0.002 |
| Wu[28] | 234 | ICD for primary prevention (NICM/ ICM) | 2-SD (infarct core FWHM) | 43 | CV death/ ICD shock | Gray zone was associated with clinical endpoint. | | |
|  |  |  |  |  |  | 2^nd^ tertile | HR 3.9 (1.2-12.4) | 0.02 |
|  |  |  |  |  |  | 3^rd^ tertile | HR 4.6 (1.4-15.4) | 0.01 |
| Mordi[29] | 157 | ICD for primary prevention (NICM/ ICM) | 5-SD | 30 | Death/ICD shock | LGE (per 1% increase) | HR 1.04 (1-01-1.07) | 0.001 |
| Almehmadi[30] | 318 | NICM/ICM | 5-SD | 15 | SCD/ ICD shock | 78% had LGE, 24% more than 1 pattern. Midwall striae involved the worst prognosis. | | |
|  |  |  |  |  |  | LGE + | HR 3.8 (1.4-10.8) | 0.01 † |
|  |  |  |  |  |  | LGE (per 1%) | HR 1.02 (1.01-1.03) | 0.008 † |
|  |  |  |  |  |  | Midwall stria | HR 2.4 (1.2-4.6) | 0.01 |
| Neilan[31] | 664 | AF | FWHM | 42 | Death | LGE extent (per 1%) | HR 1.16 (1.1-1.22) | <0.0001 |
| **Non-ischaemic cardiomyopathies** | | | |  |  |  |  |  |
| Müller[32] | 185 | NICM | Manual | 21 | Death/CT/SCD/VT/HF | LGE + | 67.4 (+)vs 27%(-) | 0.021 |
|  |  |  |  |  |  |  | HR 1.1 (0.6-2.1) | 0.676 |
|  |  |  |  |  |  | LVEF≤40% | HR 3.9 (1.9-8.1) | <0.0001 |
| Neilan[11] | 162 | NICM | FWHM  2-SD | 29 | CV death/ ventricular arrhythmia | The presence of LGE predicted clinical endpoint 🡪 sensitivity 92%, specificity 69% | | |
|  |  |  |  |  |  | LGE + | HR 6.21 (1.73-22.2) | 0.0004 |
|  |  |  |  |  |  |  | HR 1.16 per 1% (1.07-1.21) | <0.0001 |
|  |  |  |  |  |  | LGE >6.1% | AUC 0.92 |  |
| Gulati[4] | 472 | NICM | FWHM | 64 | Death, CV death, SCD, HF, CT | LGE extent | | |
|  |  |  |  |  |  | Death | HR 2.43 (1.5-3.9) | <0.001 |
|  |  |  |  |  |  |  | HR 1.11 per 1% (1.06-1.16 ) | <0.001 |
|  |  |  |  |  |  | CV death / CT | HR 3.22 (1.9-5.3) | <0.001 |
|  |  |  |  |  |  |  | HR 1.15 per 1% (1.1-1.2) | <0.001 |
|  |  |  |  |  |  | SCD | 4.61 (2.75-7.74) | <0.001 |
|  |  |  |  |  |  |  | HR 1.1 per 1% (1.05-1.16) | <0.001 |
|  |  |  |  |  |  | HF/ CT | HR 1.62 (1-2.61) | 0.049 |
|  |  |  |  |  |  |  | HR 1.08 per 1% (1-04-1.13) | <0.001 |
| Masci[33] | 228 | NICM | Manual | 23 | CV death/ HF/SCD | LGE present | HR4.02 (2.08-7.8) | <0.001 |
|  |  |  |  |  |  | LGE extent | HR 1.24 (1.11-1.38) | <0.001 |
| Assomull[34] | 101 | NICM | 2-SD | 22 | Death/CV hospitalization, SCD/VT | Midwall fibrosis is a predictor of poor outcomes | | |
|  |  |  |  |  |  | Death/CV hospital | HR 5.9 (1.1-32.2) | 0.04 |
|  |  |  |  |  |  | SCD/VT | HR 5.2 (1-26.9) | 0.03 |
| Lehrke[35] | 184 | NICM | 2-SD | 22 | CV death/ HF/ ICD shock | LGE present | 20.1(+) vs5.3%(-) | 0.002 |
|  |  |  |  |  |  |  | HR 3.37 (1.26-9) | 0.015 |
|  |  |  |  |  |  | LGE >4.4% | HR 5.28(1.8-15.5) | 0.01 |
| Perazzolo-Marra[36] | 137 | NICM | 2-SD | 36 | SCD/VT /ICD | LGE present | HR 3.8 (1.3-10.4) | 0.01 |
| Leyva[37] | 97 | NICM | Manual | 104 | Death, CV death, hospitalization for HF or MACE, | Midwall fibrosis associated with mortality/morbidity | | |
|  |  |  |  |  |  | CV death | HR 18.1 (3.5-98.5) | <0.0001 |
|  |  |  |  |  |  | Death/MACE hospitalization | HR 7.57 (2.71-21-2) | <0.0001 |
|  |  |  |  |  |  | CV death/HF hospitalization | HR 9.9 (2.72-33.6) | 0.0004 |
| Wu[38] | 65 | NICM | 2-SD | 17 | CV death/ HF/ICD shock | LGE present | 44 (+) vs 8% (-) | <0.001 |
|  |  |  |  |  |  |  | HR 8.2 (2.2-30.9) | 0.002 |
| Bruder[39] | 243 | HCM | 2-SD | 36 | Death, CV death | Death | HR 5.47 (1.24-24.1) | 0.01 † |
|  |  |  |  |  |  | CV death | HR 4.81 (1-04-61.9) | 0.035 |
| Maron[40] | 202 | HCM | 6-SD | 22 | Death/ SCD/HF | LGE was associated with LVEF (r=-0.4, p<0.001), but not with clinical events (5.5% LGE+ vs 3.3% LGE-, p=0.5) | | |
| O´Hanlon[41] | 217 | HCM | FWHM | 7 | CV death/ VT/ ICD shock | LGE presence and extent were predictors of adverse outcomes | | |
|  |  |  |  |  |  | Clinical endpoint | 25 (+) vs 7.4% (-) | 0.046 |
|  |  |  |  |  |  |  | HR 2.7 (1.01-7.1) |  |
|  |  |  |  |  |  |  | HR 1.15 per 5% (1.01-1.3) | 0.03 |
|  |  |  |  |  |  | HF | HR 2.6 (1.08-6.5) | 0.033 |
|  |  |  |  |  |  |  | HR 1.21 per 5% (1.06-1.37) | 0.004 |
| Rubinshtein[42] | 424 | HCM | Manual | 43 | VT, SCD, ICD shock | LGE was more common among those with events | | |
|  |  |  |  |  |  | Genotype + | 75% vs. 53% | <0.001 |
|  |  |  |  |  |  | NSVT | 27 vs. 8.5% | <0.001 |
|  |  |  |  |  |  | SCD/ICD shock | 3.3 vs. 0% | 0.01 |
| Chan[12] | 1293 | HCM | 6-SD | 40 | SCD | Presence and extension of LGE predicts SCD | | |
|  |  |  |  |  |  | LGE absence | HR 0.39 (0.18-0.84) | 0.002 |
|  |  |  |  |  |  | LGE extent | HR 1.46 per 10% (1.12-1.91) | 0.002 |
|  |  |  |  |  |  |  | HR 1.77 per 15% (1.22-2.43) | 0.008 |
|  |  |  |  |  |  |  | HR 2.14 per 20% (1.3-3.26) | 0.008 |
| Greulich[43] | 155 | Sarcoidosis | Manual | 31 | Death/ SCD/ICD shock | LGE+ | HR 31.6 | 0.0014 |
| Nadel[44] | 106 | Sarcoidosis | Manual | 37 | SCD, VT | LGE+ was associated with higher arrhythmic risk | | |
|  |  |  |  |  |  | SCD/VT | 38(+) vs 1.4%(-) | <0.001 |
|  |  |  |  |  |  |  | HR 12.52 (1.35-116.2) | 0.03 |
|  |  |  |  |  |  | SCD | 15.6(+) vs1.4%(-) | 0.005 |
| Patel[45] | 81 | Sarcoidosis | Manual | 22 | Death/ICD shock/PM | LGE+ | 17.2 (+) vs 1.9% (-) | § |
| Grün[46] | 203 | Myocarditis | 2-SD | 56 | Death, CV death | LGE is the best predictor of mortality | | |
|  |  |  |  |  |  | Death | HR 8.4 | 0.004 |
|  |  |  |  |  |  | CV death | HR 12.8 | <0.01 |
| Schumm[47] | 405 | Myocarditis | 2-SD | 36 | CV death/ SCD/ ICD shock | LGE + | HR 3.98 | 0.11 |
|  |  |  |  |  |  |  | HR 10.83 (2.26-51.82) | <0.001 † |
|  |  |  |  |  |  | Normal CMR | HR 0.14 (0.01-0.34) | <0.0001 |
| Fontana[48] | 250 | Amyloidosis | Transmural LGE | 24 | Death | Transmural LGE | HR: 5.4 (2.1-13.7) | <0.0001 |
| Neilan[49] | 137 | Aborted SCD (no MI) | FWHM | 29 | Death/ICD shock | LGE + | HR 6.7 (2.38-18.85) | <0.001 |
|  |  |  |  |  |  | LGE (per 1%) | HR 1.15 (1.11-1.19) | <0.001 |

**References:**

1. Kim RJ, Fieno DS, Parrish TB, Harris K, Chen EL, Simonetti O, et al. Relationship of MRI Delayed Contrast Enhancement to Irreversible Injury, Infarct Age, and Contractile Function. Circulation. 1999;100:1992–2002.

2. Fieno DS, Kim RJ, Chen EL, Lomasney JW, Klocke FJ, Judd RM. Contrast-enhanced magnetic resonance imaging of myocardium at risk: distinction between reversible and irreversible injury throughout infarct healing. J Am Coll Cardiol. 2000;36:1985–91.

3. Wagner A, Mahrholdt H, Holly TA, Elliott MD, Regenfus M, Parker M, et al. Contrast-enhanced MRI and routine single photon emission computed tomography (SPECT) perfusion imaging for detection of subendocardial myocardial infarcts: an imaging study. Lancet. 2003;361:374–9.

4. Gulati A, Jabbour A, Ismail TF, Guha K, Khwaja J, Raza S, et al. Association of fibrosis with mortality and sudden cardiac death in patients with nonischemic dilated cardiomyopathy. JAMA. 2013;309:896–908.

5. Iles LM, Ellims AH, Llewellyn H, Hare JL, Kaye DM, McLean CA, et al. Histological validation of cardiac magnetic resonance analysis of regional and diffuse interstitial myocardial fibrosis. Eur Heart J Cardiovasc Imaging. 2015;16:14–22.

6. Thiele H, Kappl MJE, Conradi S, Niebauer J, Hambrecht R, Schuler G. Reproducibility of Chronic and Acute Infarct Size Measurement by Delayed Enhancement-Magnetic Resonance Imaging. J Am Coll Cardiol. 2006;47:1641–5.

7. Desch S, Engelhardt H, Meissner J, Eitel I, Sareban M, Fuernau G, et al. Reliability of myocardial salvage assessment by cardiac magnetic resonance imaging in acute reperfused myocardial infarction. Int J Cardiovasc Imaging. 2012;28:263–72.

8. Flett AS, Hasleton J, Cook C, Hausenloy D, Quarta G, Ariti C, et al. Evaluation of Techniques for the Quantification of Myocardial Scar of Differing Etiology Using Cardiac Magnetic Resonance. JACC: Cardiovascular Imaging. 2011;4:150–6.

9. McAlindon E, Pufulete M, Lawton C, Angelini GD, Bucciarelli-Ducci C. Quantification of infarct size and myocardium at risk: evaluation of different techniques and its implications. Eur Heart J Cardiovasc Imaging. 2015;16:738–46.

10. Khan JN, Nazir SA, Horsfield MA, Singh A, Kanagala P, Greenwood JP, et al. Comparison of semi-automated methods to quantify infarct size and area at risk by cardiovascular magnetic resonance imaging at 1.5T and 3.0T field strengths. BMC Research Notes [Internet]. BioMed Central; 2015;8:52. Available from: http://www.biomedcentral.com/1756-0500/8/52

11. Neilan TG, Coelho-Filho OR, Danik SB, Shah RV, Dodson JA, Verdini DJ, et al. CMR Quantification of Myocardial Scar Provides Additive Prognostic Information in Nonischemic Cardiomyopathy. JACC Cardiovasc Imaging. 2013;6:944–54.

12. Chan RH, Maron BJ, Olivotto I, Pencina MJ, Assenza GE, Haas T, et al. Prognostic Value of Quantitative Contrast-Enhanced Cardiovascular Magnetic Resonance for the Evaluation of Sudden Death Risk in Patients With Hypertrophic Cardiomyopathy. Circulation. 2014;130:484–95.

13. Wagner A, Mahrholdt H, Holly TA, Elliott MD, Regenfus M, Parker M, et al. Contrast-enhanced MRI and routine single photon emission computed tomography (SPECT) perfusion imaging for detection of subendocardial myocardial infarcts: an imaging study. Lancet. Elsevier; 2003;361:374–9.

14. Ibrahim T, Bülow HP, Hackl T, Hörnke M, Nekolla SG, Breuer M, et al. Diagnostic value of contrast-enhanced magnetic resonance imaging and single-photon emission computed tomography for detection of myocardial necrosis early after acute myocardial infarction. J Am Coll Cardiol. 2007;49:208–16.

15. Wu Y-W, Tadamura E, Kanao S, Yamamuro M, Marui A, Komeda M, et al. Myocardial viability by contrast-enhanced cardiovascular magnetic resonance in patients with coronary artery disease: comparison with gated single-photon emission tomography and FDG position emission tomography. Int J Cardiovasc Imaging [Internet]. Springer Netherlands; 2007;23:757–65. Available from: http://link.springer.com/10.1007/s10554-007-9215-y

16. Larose E, Rodés-Cabau J, Pibarot P, Rinfret S, Proulx G, Nguyen CM, et al. Predicting late myocardial recovery and outcomes in the early hours of ST-segment elevation myocardial infarction traditional measures compared with microvascular obstruction, salvaged myocardium, and necrosis characteristics by cardiovascular magnetic resonance. J Am Coll Cardiol. 2010;55:2459–69.

17. Wu E, Ortiz JT, Tejedor P, Lee DC, Bucciarelli-Ducci C, Kansal P, et al. Infarct size by contrast enhanced cardiac magnetic resonance is a stronger predictor of outcomes than left ventricular ejection fraction or end-systolic volume index: prospective cohort study. Heart [Internet]. 2008;94:730–6. Available from: http://heart.bmj.com/cgi/doi/10.1136/hrt.2007.122622

18. Hadamitzky M, Langhans B, Hausleiter J, Sonne C, Byrne RA, Mehilli J, et al. Prognostic value of late gadolinium enhancement in cardiovascular magnetic resonance imaging after acute ST-elevation myocardial infarction in comparison with single-photon emission tomography using Tc99m-Sestamibi. European Heart Journal - Cardiovascular Imaging. 2014;15:216–25.

19. Eitel I, de Waha S, Wöhrle J, Fuernau G, Lurz P, Pauschinger M, et al. Comprehensive Prognosis Assessment by CMR Imaging After ST-Segment Elevation Myocardial Infarction. J Am Coll Cardiol. [Internet]. 2014;64:1217–26. Available from: http://linkinghub.elsevier.com/retrieve/pii/S0735109714056241

20. Eitel I, Desch S, Fuernau G, Hildebrand L, Gutberlet M, Schuler G, et al. Prognostic Significance and Determinants of Myocardial Salvage Assessed by Cardiovascular Magnetic Resonance in Acute Reperfused Myocardial Infarction. J Am Coll Cardiol. 2010;55:2470–9.

21. Stone GW, Selker HP, Thiele H, Patel MR, Udelson JE, Ohman EM, et al. Relationship Between Infarct Size and Outcomes Following Primary PCI. J Am Coll Cardiol. 2016;67:1674–83.

22. de Waha S, Desch S, Eitel I, Fuernau G, Zachrau J, Leuschner A, et al. Impact of early vs. late microvascular obstruction assessed by magnetic resonance imaging on long-term outcome after ST-elevation myocardial infarction: a comparison with traditional prognostic markers. Eur Heart J. The Oxford University Press; 2010;31:2660–8.

23. Steel K, Broderick R, Gandla V, Larose E, Resnic F, Jerosch-Herold M, et al. Complementary Prognostic Values of Stress Myocardial Perfusion and Late Gadolinium Enhancement Imaging by Cardiac Magnetic Resonance in Patients With Known or Suspected Coronary Artery Disease. Circulation [Internet]. American Heart Association, Inc; 2009;120:1390–400. Available from: http://circ.ahajournals.org/cgi/doi/10.1161/CIRCULATIONAHA.108.812503

24. Kwong RY, Chan AK, Brown KA, Chan CW, Reynolds HG, Tsang S, et al. Impact of Unrecognized Myocardial Scar Detected by Cardiac Magnetic Resonance Imaging on Event-Free Survival in Patients Presenting With Signs or Symptoms of Coronary Artery Disease. Circulation [Internet]. American Heart Association, Inc; 2006;113:2733–43. Available from: http://circ.ahajournals.org/cgi/doi/10.1161/CIRCULATIONAHA.105.570648

25. Iles L, Pfluger H, Lefkovits L, Butler MJ, Kistler PM, Kaye DM, et al. Myocardial Fibrosis Predicts Appropriate Device Therapy in Patients With Implantable Cardioverter-Defibrillators for Primary Prevention of Sudden Cardiac Death. J Am Coll Cardiol. 2011;57:821–8.

26. Gao P, Yee R, Gula L, Krahn AD, Skanes A, Leong-Sit P, et al. Prediction of Arrhythmic Events in Ischemic and Dilated Cardiomyopathy Patients Referred for Implantable Cardiac Defibrillator: Evaluation of Multiple Scar Quantification Measures for Late Gadolinium Enhancement Magnetic Resonance Imaging. Circ Cardiovasc Imaging [Internet]. American Heart Association, Inc; 2012;5:448–56. Available from: http://circimaging.ahajournals.org/cgi/doi/10.1161/CIRCIMAGING.111.971549

27. Klem I, Weinsaft JW, Bahnson TD, Hegland D, Kim HW, Hayes B, et al. Assessment of Myocardial Scarring Improves Risk Stratification in Patients Evaluated for Cardiac Defibrillator Implantation. J Am Coll Cardiol. [Internet]. 2012;60:408–20. Available from: http://linkinghub.elsevier.com/retrieve/pii/S0735109712017032

28. Wu KC, Gerstenblith G, Guallar E, Marine JE, Dalal D, Cheng A, et al. Combined Cardiac Magnetic Resonance Imaging and C-Reactive Protein Levels Identify a Cohort at Low Risk for Defibrillator Firings and Death. Circ Cardiovasc Imaging [Internet]. American Heart Association, Inc; 2012;5:178–86. Available from: http://circimaging.ahajournals.org/cgi/doi/10.1161/CIRCIMAGING.111.968024

29. Mordi I, Jhund PS, Gardner RS, Payne J, Carrick D, Berry C, et al. LGE and NT-proBNP Identify Low Risk of Death or Arrhythmic Events in Patients With Primary Prevention ICDs. JACC Cardiovasc Imaging [Internet]. 2014;7:561–9. Available from: http://linkinghub.elsevier.com/retrieve/pii/S1936878X1400237X

30. Almehmadi F, Joncas SX, Nevis I, Zahrani M, Bokhari M, Stirrat J, et al. Prevalence of Myocardial Fibrosis Patterns in Patients With Systolic Dysfunction: Prognostic Significance for the Prediction of Sudden Cardiac Arrest or Appropriate Implantable Cardiac Defibrillator Therapy. Circ Cardiovasc Imaging [Internet]. American Heart Association, Inc; 2014;7:593–600. Available from: http://circimaging.ahajournals.org/cgi/doi/10.1161/CIRCIMAGING.113.001768

31. Neilan TG, Shah RV, Abbasi SA, Farhad H, Groarke JD, Dodson JA, et al. The Incidence, Pattern, and Prognostic Value of Left Ventricular Myocardial Scar by Late Gadolinium Enhancement in Patients With Atrial Fibrillation. J Am Coll Cardiol. [Internet]. 2013;62:2205–14. Available from: http://linkinghub.elsevier.com/retrieve/pii/S0735109713038783

32. Müller KAL, Müller I, Kramer U, Kandolf R, Gawaz M, Bauer A, et al. Prognostic Value of Contrast-enhanced Cardiac Magnetic Resonance Imaging in Patients with Newly Diagnosed Non-Ischemic Cardiomyopathy: Cohort Study. Marian AJ, editor. PLoS ONE [Internet]. 2013;8:e57077. Available from: http://dx.plos.org/10.1371/journal.pone.0057077

33. Masci PG, Doulaptsis C, Bertella E, Del Torto A, Symons R, Pontone G, et al. Incremental Prognostic Value of Myocardial Fibrosis in Patients With Non-Ischemic Cardiomyopathy Without Congestive Heart Failure. Circulation: Heart Failure [Internet]. American Heart Association, Inc; 2014;7:448–56. Available from: http://circheartfailure.ahajournals.org/cgi/doi/10.1161/CIRCHEARTFAILURE.113.000996

34. Assomull RG, Prasad SK, Lyne J, Smith G, Burman ED, Khan M, et al. Cardiovascular Magnetic Resonance, Fibrosis, and Prognosis in Dilated Cardiomyopathy. J Am Coll Cardiol. [Internet]. 2006;48:1977–85. Available from: http://linkinghub.elsevier.com/retrieve/pii/S0735109706021115

35. Lehrke S, Lossnitzer D, Schob M, Steen H, Merten C, Kemmling H, et al. Use of cardiovascular magnetic resonance for risk stratification in chronic heart failure: prognostic value of late gadolinium enhancement in patients with non-ischaemic dilated cardiomyopathy. Heart [Internet]. BMJ Publishing Group Ltd; 2011;97:727–32. Available from: http://heart.bmj.com/cgi/doi/10.1136/hrt.2010.205542

36. Perazzolo Marra M, De Lazzari M, Zorzi A, Migliore F, Zilio F, Calore C, et al. Impact of the presence and amount of myocardial fibrosis by cardiac magnetic resonance on arrhythmic outcome and sudden cardiac death in nonischemic dilated cardiomyopathy. Heart Rhythm. 2014;11:856–63.

37. Leyva F, Taylor RJ, Foley PWX, Umar F, Mulligan LJ, Patel K, et al. Left Ventricular Midwall Fibrosis as a Predictor of Mortality and Morbidity After Cardiac Resynchronization Therapy in Patients With Nonischemic Cardiomyopathy. J Am Coll Cardiol. 2012;60:1659–67.

38. Wu KC, Weiss RG, Thiemann DR, Kitagawa K, Schmidt A, Dalal D, et al. Late Gadolinium Enhancement by Cardiovascular Magnetic Resonance Heralds an Adverse Prognosis in Nonischemic Cardiomyopathy. J Am Coll Cardiol. [Internet]. 2008;51:2414–21. Available from: http://linkinghub.elsevier.com/retrieve/pii/S0735109708011133

39. Bruder O, Wagner A, Jensen CJ, Schneider S, Ong P, Kispert E-M, et al. Myocardial Scar Visualized by Cardiovascular Magnetic Resonance Imaging Predicts Major Adverse Events in Patients With Hypertrophic Cardiomyopathy. J Am Coll Cardiol. 2010;56:875–87.

40. Maron MS, Appelbaum E, Harrigan CJ, Buros J, Gibson CM, Hanna C, et al. Clinical Profile and Significance of Delayed Enhancement in Hypertrophic Cardiomyopathy. Circulation: Heart Failure [Internet]. American Heart Association, Inc; 2008;1:184–91. Available from: http://circheartfailure.ahajournals.org/cgi/doi/10.1161/CIRCHEARTFAILURE.108.768119

41. O'Hanlon R, Grasso A, Roughton M, Moon JC, Clark S, Wage R, et al. Prognostic Significance of Myocardial Fibrosis in Hypertrophic Cardiomyopathy. J Am Coll Cardiol. [Internet]. 2010;56:867–74. Available from: http://linkinghub.elsevier.com/retrieve/pii/S0735109710019169

42. Rubinshtein R, Glockner JF, Ommen SR, Araoz PA, Ackerman MJ, Sorajja P, et al. Characteristics and Clinical Significance of Late Gadolinium Enhancement by Contrast-Enhanced Magnetic Resonance Imaging in Patients With Hypertrophic Cardiomyopathy. Circulation: Heart Failure [Internet]. American Heart Association, Inc; 2010;3:51–8. Available from: http://circheartfailure.ahajournals.org/cgi/doi/10.1161/CIRCHEARTFAILURE.109.854026

43. Greulich S, Deluigi CC, Gloekler S, Wahl A, Zürn C, Kramer U, et al. CMR Imaging Predicts Death and Other Adverse Events in Suspected Cardiac Sarcoidosis. JACC: Cardiovascular Imaging. 2013;6:501–11.

44. Nadel J, Lancefield T, Voskoboinik A, Taylor AJ. Late gadolinium enhancement identified with cardiac magnetic resonance imaging in sarcoidosis patients is associated with long-term ventricular arrhythmia and sudden cardiac death. Eur Heart J Cardiovasc Imaging [Internet]. 2015. Available from: https://academic.oup.com/ehjcimaging/article-lookup/doi/10.1093/ehjci/jeu294

45. Patel MR, Cawley PJ, Heitner JF, Klem I, Parker MA, Jaroudi WA, et al. Detection of Myocardial Damage in Patients With Sarcoidosis. Circulation. 2009;120:1969–77.

46. Grün S, Schumm J, Greulich S, Wagner A, Schneider S, Bruder O, et al. Long-Term Follow-Up of Biopsy-Proven Viral Myocarditis. J Am Coll Cardiol.. 2012;59:1604–15.

47. Schumm J, Greulich S, Wagner A, Grün S, Ong P, Bentz K, et al. Cardiovascular magnetic resonance risk stratification in patients with clinically suspected myocarditis. J Cardiovasc Magn Reson [Internet]. BioMed Central; 2014;16:14. Available from: http://jcmr-online.biomedcentral.com/articles/10.1186/1532-429X-16-14

48. Fontana M, Pica S, Reant P, Abdel-Gadir A, Treibel TA, Banypersad SM, et al. Prognostic Value of Late Gadolinium Enhancement Cardiovascular Magnetic Resonance in Cardiac Amyloidosis. Circulation. American Heart Association, Inc; 2015;132:1570–9.

49. Neilan TG, Farhad H, Mayrhofer T, Shah RV, Dodson JA, Abbasi SA, et al. Late Gadolinium Enhancement Among Survivors of Sudden Cardiac Arrest. JACC Cardiovasc Imaging [Internet]. 2015;8:414–23. Available from: http://linkinghub.elsevier.com/retrieve/pii/S1936878X1500090X
