# Supplementary material for: Society for Cardiovascular Magnetic Resonance (SCMR) expert consensus for CMR imaging endpoints in clinical research: part I - analytical validation and clinical qualification
Source: J Cardiovasc Magn Reson. 2018 Sep 20;20:67. doi: 10.1186/s12968-018-0484-5 (PMC6147157; doi:10.1186/s12968-018-0484-5)
Supplement: Supplementary file 3 — T1 mapping tables. Table 3c-i.1: Overview of the T1 mapping indices. Table 3c-i.2: Histological correlations with T1 mapping indices in various cardiac conditions. Table 3c-i.3: Intra, interobserver and interstudy variability reported for various sequences and field strengths. Table 3c-i.4: Overview of studies reporting normative ranges for T1 mapping indices. Table 3c-i.5: Proof of concept studies using T1 mapping in health and disease. Table 3c-i.6: Outcome studies for all-cause mortality (A) and composite cardiac/heart failure (B) endpoints. (DOCX 113 kb) [file 12968_2018_484_MOESM3_ESM.docx]

## T1 mapping tables

**Table 3c-i.1**. Overview of the T1 mapping indices. ECV – extracellular volume fraction, Ht – hematocrit, ECS – extracellular space, GCAs – gadolinium contrast agent, ROI – region of interest, CMR – cardiovascular magnetic resonance.

| **Index** | **Native T1** | **Postcontrast T1** | **ECV** |
| --- | --- | --- | --- |
| Measurement | Direct myocardial T1 measurement | Direct myocardial T1 measurement | Calculation based on T1 measurement of   - native and postcontrast myocardium - native and postcontrast blood pool   and Hematocrit (Ht). |
| Calculation | Exponential fit of data-points obtained during T1 relaxation | as for native T1 | (1/T1_postcontrastMyo_ - 1/T1_nativeMyo_)  ECV= (1-Ht) * _______________________________________  (1/ T1_postcontrastBlood_ - 1/T1_nativeBlood_) |
| Main source of influence | Intracellular and extracellular compartment of native myocardium | Gadolinium effects in extracellular compartment | Gadolinium effects in extracellular compartment |
| Histological relationships | - Intra- and extracellular edema - Interstitial fibrosis - Scar (replacement fibrosis) - Amyloid infiltration - Iron accumulation* - Lipid infiltration*   * = causing reduction of T1 | Expanded extracellular space:   - Replacement fibrosis - Interstitial fibrosis - Extracellular edema - Amyloid infiltration | Expanded extracellular space:   - Replacement fibrosis - Interstitial fibrosis - Extracellular edema - Amyloid infiltration |
| Strengths | 1. Simplicity  - Single breath-hold acquisition - Contrast-agent free  1. Reproducible postprocessing (based on septal ROI) 2. Normal ranges and discrimination between health and disease 3. Outcome data available | 1. Simplicity  - Single breath-hold acquisition  1. Close relationship with ECS by tracking of the effects of the GCAs 2. Allows assessment of regional heterogeneity | 1. Close relationship with ECS by tracking of the effects of the GCAs 2. Allows assessment of regional heterogeneity 3. Outcome data available |
| Weaknesses and limitations | 1. Normal values sequence specific 2. Diagnostic accuracy sequence dependent (T2 sensitive) 3. Nonspecific for multiple underlying abnormalities 4. Assessment of regional heterogeneity possible in artefact free images | 1. Difficult standardization:   - No normal ranges - Intra and inter-individual variations in renal clearance - Differences between GCAs  1. Reliance on GCAs 2. Reliance on T1 accuracy | 1. Reliance on several measurements obtained ~15 min apart 2. Accumulation of errors with dispersion of values 3. Difficult standardization:    - Dispersion of values    - Intra and inter-individual variations in renal clearance    - Differences between GCAs 4. Nonspecific for multiple underlying abnormalities 5. Requires GCAs 6. Requires contemporaneous hematocrit 7. Reliance on T1 accuracy (difficult in water rich myocardium) |
| Possible applications | - Screening for subclinical disease - Risk stratification - Grading of disease severity - Monitoring treatment response - Contrast free CMR | - Marker of expanded extracellular space - Assessment of regional heterogeneity | - Marker of expanded extracellular space - Risk stratification - Grading of disease severity |

**Table 3c-i.2**. **Histological correlations with T1 mapping indices in various cardiac conditions.** Types of sequences and a staining method used, as well as a number of patients included, is also reported. Discriminative focus on replacement vs. interstitial fibrosis is a paramount factor influencing the diversity of observed relationships; whereas some authors steered away from the inclusion of replacement fibrosis/LGE affected areas in histological CVF (or accounted for them separately)(1-4)**,** or all-inclusive(5-7). IHD – ischaemic heart disease, VAST variable sampling of k-space in time, NICM – non-ischemic cardiomyopathy, HFpEF – heart failure with preserved ejection fraction, DCM – dilated cardiomyopathy, FLASH-IR - fast low-angle shot – inversion recovery. *shMOLLI is a MOLLI (5(1)1(1)1 (FA 35°) variant utilizing a conditional reconstruction algorithm.

| **Collagen volume fraction%** | **N of patients (cardiac disease)** | **Sequence** | **GCAs (dose and type)** | **T1 Index** | | **Staining** | | **Pearson r (Sig)** | |
| --- | --- | --- | --- | --- | --- | --- | --- | --- | --- |
| ***Heart failure*** | |  |  | |  | |  | |  |
| Iles(1) | 9 (IHD) | VAST | (0.2 mmol/kg gadopentetate dimeglumine) | Postcontrast T1 | | picrosirius red | | -0.7(0.03) | |
| Sibley(8) | 47 (NICMs) | Look-Locker | (0.2 mmol/kg gadodiamide) | Postcontrast T1 | | Masson trichrome | | −0.57 (<0.001) | |
| [Mascherbauer](http://www.ncbi.nlm.nih.gov/pubmed/?term=Mascherbauer%20J%5BAuthor%5D&cauthor=true&cauthor_uid=24036385) (9) | 9 (HFpEF) | FLASH-IR | (0.2 mmol/kg gadobutrol) | Postcontrast T1 | | Masson Trichrome/Congo-red | | -0.98 (<0.01) | |
| Miller(5) | 6 (IHD) | MOLLI 3(3)3(3)5(FA 35°) | (0.2 mmol/kg (gadopentetate dimeglumine) | Native T1 | | picrosirius red | | 0.199 (0.437) | |
|  |  |  |  | Postcontrast T1 | |  |  | −0.21 (0.69) | |
|  |  |  |  | ECV (bolus) | |  |  | 0.945 (0.004) | |
| Aus dem Siepen(6) | 45 (DCM) | MOLLI 3(3)3(3)5(FA 35°) | (0.2 mmol/kg gadopentetate dimeglumine) | ECV (bolus) | | Acid Fuchsin Orange-G | | 0.85 (0.01) | |
| Iles(4) | 4 (1 IHD, 3 DCM) | VAST | (0.2 mmol/kg gadopentetate dimeglumine) | LGE | | Masson Trichrome | | 0.73 (<0.001) | |
|  |  |  |  | Postcontrast T1 | |  |  | -0.64 (0.002) | |
| Kammerlander(2) | 36 (mixed group) | MOLLI 5(3)3 (FA 35°) for native acquisition  MOLLI 4(1)3(1)2(FA 35°) for postcontrast acquisition | (0.1 mmol/kg of gadobutrol) | ECV (bolus) | | Tissue FAXS | | 0.493 (<0.002) | |
| ***Aortic stenosis*** | |  |  | |  | |  | |  |
| Flett(10) | 18 | FLASH-IR | (0.2 mmol/kg gadoterate meglumine) | ECV (EQ) | | picrosirius red | | 0.94 (R^2^= 0.89, 0.001) (Tau=0.71) | |
| Bull(3) | 19 | *shMOLLI |  | Native T1 | | picrosirius red | | 0.655 (0.002) | |
| Fontana(11) | 18 | FLASH-IR | (0.2 mmol/kg gadoterate meglumine) | ECV (EQ) | | picrosirius red | | 0.78 (R^2^=0.589, p<0.01) | |
|  |  | *shMOLLI |  |  |  |  |  | 0.83 (R^2^=0.685, <0.01) | |
| White(12) | 18 | *shMOLLI | (0.2 mmol/kg gadoterate meglumine) | ECV (bolus)  ECV (EQ) | | picrosirius red | | 0.83 (R^2^=0.69, <0.01)  0.84 (R^2^=0.71, <0.01) | |
| de Meester de Ravenstein(7) | 12 | MOLLI 3(3)3(3)5 (FA35°) | (0.2 mmol/kg gadobutrol) | Native T1 | | picrosirius red | | -0.15 (0.64) | |
|  |  |  |  | Postcontrast T1 | |  |  | -0.64 (0.024) | |
|  |  |  |  | ECV | |  |  | 0.91 (0.001) | |
| Lee(13) | 10 | MOLLI 3(3)3(3)5(FA35°) |  | Native T1 | | picrosirius red | | 0.77 (<0.01) | |
| ***Hypertrophic cardiomyopathy*** | |  |  | |  | |  | |  |
| Flett(10) | 8 | FLASH-IR | (0.2 mmol/kg gadoterate meglumine) | ECV | | picrosirius red | | R2=0.62(0.08), Tau=0.52 | |
| Iles(4) | 8 | VAST | (0.2 mmol/kg gadopentetate dimeglumine) | Postcontrast T1 | | Masson-trichrome | | -0.71 (0.01) | |

**Table 3c-i.3**. **Intra, interobserver and interstudy variability reported for various sequences and field strengths.**

Septal ROIs (14-16), SAX ROIs for others. Results are reported as Bland-Altman plots: MD±SD (or ±SD) and CoV in brackets when available. AS: aortic stenosis. shMOLLI is a MOLLI (5(1)1(1)1 (FA 35°) variant utilizing a conditional reconstruction algorithm.

|  | **Dabir**(14) | | | **Ferreira** (15) | **Dass** (16) | **Rogers**(17) | | | | | | **Von Knobelsdorff**(18) | **Liu**(19) | **Messroghli**(20) | **Singh**(21) | | **Pica**(22) | | **Weingärtner**(23) | |
| --- | --- | --- | --- | --- | --- | --- | --- | --- | --- | --- | --- | --- | --- | --- | --- | --- | --- | --- | --- | --- |
| **Magnetic field (T)** | 1.5/3.0 | | | 1.5 | 3.0 | 1.5 | | | 3.0 | | | 3.0 | 3.0 | 1.5 | 3.0 | | 1.5 | | 3.0 | |
| **N** | 10 | | | 42 | 8 | 56 | | | 44 | | | 20 | 24 | 15 | 10 | | 21 | | 20 | |
| **Population** | Healthy volunteers | | | Healthy volunteers and patients | Healthy volunteers and patients | Patients | | | Patients | | | Healthy volunteers | Healthy volunteers | Healthy volunteers | AS | | Fabry disease | | Healthy volunteers | |
| **T1 index** | Native T1 | | Post T1 | Native T1 | Native T1 | Native T1 | Post T1 | Lambda | Native T1 | Post T1 | Lambda | Native T1 | ECV | Native T1 | Native T1 | Post T1 | Native T1 | | Native T1 | Post T1 |
| **Sequence** | MOLLI 3(2)3(2)5 (FA 50º) | | | *shMOLLI | *shMOLLI | MOLLI 3(2)3(2)5 (FA 50º) | | | | | | MOLLI 3(3)3(3)5 (FA 35º) | MOLLI 3(3)5 (FA 35º) | MOLLI 3’3’5 (FA 50º) | MOLLI 3(3)3(3)5 (FA 50º) | | MOLLI 5(3)3 (FA35º) | *shMOLLI | MOLLI 3(3)5 (FA 35º) | MOLLI 4(1)3(1)2 (FA 35º) |
|  |  |  |  |  |  |  |  |  |  |  |  |  |  |  |  |  |  |  | SAPPHIRE | |
|  |  |  |  |  |  |  |  |  |  |  |  |  |  |  |  |  |  |  | SASHA | |
| **Inter-observer V** | 1.3±6.8 (6.1%) | -5.9±9.8 (16.2%) | | ±5.6 | ±24 | 1.5±19 (4.3%) | 6.3±52 (4.3%) | 13 ± 19 (7.2%) | 3 ± 13  (1.2%) | −6 ± 15  (3.2%) | 17 ± 20  (5.1%) | 0.5±20.2 | -6±17 (6.4%) | -1.1±8.9 (0.9%) | -2.3±3.7 (0.34%) | 0±5 (2.31) | (1.1%) | -0.8± 2.9% (1.4%) | 11.3 | 2.8 |
|  |  |  |  |  |  |  |  |  |  |  |  |  |  |  |  |  |  |  | 13.0 | 5.3 |
|  |  |  |  |  |  |  |  |  |  |  |  |  |  |  |  |  |  |  | 8.8 | 5.3 |
| **Intra-observer V** | 2.1±4.3 (5.2%) | -4.8±7.2 (12.6%) | | ±5.6 |  | 3±11 (1.1%) | 5±12 (3.1%) | 14 ± 9  (4.1%) | 0.3 ± 15  (1.4%) | 6.2 ± 71  (2.8%) | 19 ± 8  (9.1%) | 4.6±18.3 | 7±06 (2.2%) | 2.6±6.7 (0.7%) | 0.18±5.6 (0.5%) | -1± 4 (1.8%) | (1.2%) | -0.3± 2.2% (1.5%) | 7.1 | 3.6 |
|  |  |  |  |  |  |  |  |  |  |  |  |  |  |  |  |  |  |  | 5.1 | 4.1 |
|  |  |  |  |  |  |  |  |  |  |  |  |  |  |  |  |  |  |  | 3.3 | 3.2 |
| **Inter-study V** |  |  | |  |  | 2.4 ± 9.2  (1.2%) | −8 ± 54  (9.0%) | 0.017 ± 0.021  (4.2%) | −1.5 ± 12  (3.6%) | 19 ± 65  (12%) | 0.016 ± 0.018  (3.5%) |  |  |  | -8.2± 19.3 (1.77%) | 2±0.15 (6.5%) | (1.5%) | -0.3±2.2% (0.8%) |  | |

**Table 3c-i.4. Overview of studies reporting normative ranges for T1 mapping indices**. Studies included if n>50 subjects. Number of participants per group, mean values (mean±SD) are reported for the type of sequence, T1 index and field strength, T1 mapping indices. Postcontrast T1measurements were typically obtained > 15 min after contrast administration. *shMOLLI is a MOLLI (5(1)1(1)1 (FA 35°) variant utilizing a conditional reconstruction algorithm.

| **Study (n = participants)** |  |  |  | **1.5 T** |  | **3.0 T** |  |
| --- | --- | --- | --- | --- | --- | --- | --- |
|  | **Sequence** | **GCAs (dose and type)** | **T1 index** | **Myocardium** | **Blood** | **Myocardium** | **Blood** |
| Piechnik(24) (n= 342) | *shMOLLI |  | Native T1 (ms) | 962±25 | 1535±76 |  |  |
| Dabir(14) (n=102) | MOLLI 3(2)3(2)5 (FA 50º) | (0.1-0.2 mmol/kg gadobutrol) | Native T1 (ms) | 950 ± 21 | 1551 ± 115 | 1052 ± 23 | 1736 ± 139 |
|  |  |  | ECV (%) | 25±4 |  | 26±4 |  |
| Liu(25) (n=1231) | MOLLI 3(3)3(3)5(FA 35°) | (0.15 mmol/kg gadopentetate dimeglumine) | Native T1 (ms) | 977 ± 42 |  |  |  |
|  |  |  | ECV% | 26.9±2.8 |  |  |  |
| Von Knobelsdorff(18) (n=60) | MOLLI 3(3)3(3)5(FA 35°) |  | Native T1 (ms) |  |  | 1159±≈73 |  |

**Table 3c-i.5. Proof of concept studies using T1 mapping in health and disease.** Studies included if n>25 subjects/patients’ group. Number of participants per group, mean values (mean±SD) are reported for disease entity, the type of sequence, T1 index and field strength, including effect size as a measure of dispersion observed in healthy subjects, as well as the Cohen’s d index. The order relates to the order referencing. *shMOLLI is a MOLLI (5(1)1(1)1 (FA 35°) variant utilizing a conditional reconstruction algorithm; **merged results from 1.5 and 3.0 T field strength.

|  |  | **Health (n)** | | **Disease (n)** | |  |
| --- | --- | --- | --- | --- | --- | --- |
|  | **Sequence (dose and type of gadolinium contrast agent)** | **Myocardial T1 index** | | | | **Effect size** **(Cohen’s d)** |
|  | | 1.5 T | 3.0 T | 1.5 T | 3.0 T |  |
| **Amyloidosis (ATTR)** | | **Native T1 (ms)** | | | |  |
| Fontana(26) | *shMOLLI | 967 ±34  (n=52) |  | 1097±43  (n=85) |  | 3.5 |
|  |  | **Postcontrast T1 (ms)** | | | |  |
| **Amyloidosis (AL)** | | **Native T1 (ms)** | | | |  |
| Banypersad(27) | *shMOLLI | 954±34  (n=54) |  | 1080±87  (n=100) |  | 1.9 |
| Karamitsos(28) | *shMOLLI | 958±20  (n=36) |  | 1140± 61  (n=53) |  | 3.5 |
| Fontana(26) | *shMOLLI | 967 ±34  (n=52) |  | 1130±68  (n=79) |  | 3.0 |
|  |  | **ECV (%)** | | | |  |
| Banypersad(27) | *shMOLLI  (0.1 mmol/kg gadoterate meglumine) | 25±2  (n=54) |  | 44±12  (n=100) |  | 2.3 |
| Fontana(26) | shMOLLI  (0.1 mmol/kg gadoterate meglumine) | 27±3  (n=50) |  | 52±7  (n=20) |  | 4.3 |
| **Aortic stenosis** | | **Native T1 (ms)** | | | |  |
| Bull(3) | *shMOLLI | 944±16  (n=33) |  | 971±39 (n=109) |  | 0.9 |
| Mahmod(29) | *shMOLLI |  | 1168±27  (n=16) |  | 1196 ± 47  (n=26) | 0.7 |
| Lee(13) | MOLLI 3(3)3(3)5 (FA 35°) |  | 1169±21 (n=15) |  | 1214±45 (n=62) | 1.3 |
| Singh(21) | MOLLI 3(3)3(3)5 (FA 50°) |  | 1092±35 (n=22) |  | 1103±33 (n=50) | 0.32 |
|  |  | **ECV (%)** | | | |  |
| Singh(21) | MOLLI 3(3)3(3)5 (FA 50°)  (0.15 mmol/kg of gadobutrol) |  | 24±2 (n=22) |  | 25±3 (n=50) | 0.4 |
| Fontana(26) | *shMOLLI  (0.1 mmol/kg gadoterate meglumine) | 27±3  (n=50) |  | 31±5  (n=50) |  | 1.0 |
| **DCM (non-ischemic)** | | **Native T1 (ms)** | | | |  |
| Puntmann(30) | MOLLI 3(2)3(2)5 (FA 50º) |  | 1070±55 (n=30) |  | 1239±57 (n=27) | 3.0 |
| Puntmann(31) | MOLLI 3(2)3(2)5 (FA 50º) |  | 1055±22 (n=47) |  | 1115±37 (n=82) | 1.9 |
| Aus dem Siepen(6) | MOLLI 3(3)5 (FA 35°) | 1020±40  (n=56) |  | 1056±62  (n=29) |  | 0.9 |
|  |  | **Post-contrast T1(ms)** | | | |  |
| Puntmann(30) | MOLLI 3(2)3(2)5 (FA 50º)  (0.2 mmol/kg of gadobutrol |  | 440±58 (n=30) |  | 355±44 (n=27) | 1.6 |
| Aus dem Siepen(6) | MOLLI 3(3)5 (FA 35°)  (0.2 mmol/kg gadopentetate dimeglumine) | 442±43  (n=56) |  | 420±45  (n=29) |  | 0.5 |
|  |  | **ECV (%)** | | | |  |
| Puntmann(30) | MOLLI 3(2)3(2)5 (FA 50º)  (0.2 mmol/kg of gadobutrol) |  | 27±10 (n=30) |  | 41±10 (n=27) | 1.4 |
| Aus dem Siepen(6) | MOLLI 3(3)5 (FA 35°)  (0.2 mmol/kg gadopentetate dimeglumine) | 23±3  (n=56) |  | 27±4  (n=29) |  | 1.1 |
| Puntmann(31) | MOLLI 3(2)3(2)5 (FA 50º)  (0.2 mmol/kg of gadobutrol) |  | 27±9 (n=47) |  | 40±9  (n=82) | 1.4 |
| Ugander(32)  LGE-  LGE+ | MOLLI 3(3)5 (FA 35°)  (0.15 mmol/kg gadopentetate dimeglumine) | (n=11)  25±3 |  | (n=30)  26±3  37±6 |  | 0.3  2.6 |
| **Hypertension** | | **Native T1 (ms)** | | | |  |
| Hinojar(33) | MOLLI 3(2)3(2)5 (FA 50º) |  | 1044±18 (n=23) |  | 1058±29 (n=69) | 0.6 |
| Treibel(34) | *shMOLLI | 965±38  (n=50) |  | 955±30 (n=46) |  | 0.3 |
|  |  | **Post-contrast T1 (ms)** | | | |  |
| Hinojar(33) | MOLLI 3(2)3(2)5 (FA 50º)  (0.2 mmol/kg of gadobutrol) |  | 446±70 (n=23) |  | 429±60 (n=69) | 0.3 |
| Treibel(34) | *shMOLLI  (0.1 mmol/kg of gadoterate meglumine) | 618±33  (n=50) |  | 578±37  (n=46) |  | 1.1 |
|  |  | **ECV (%)** | | | |  |
| Hinojar(33) | MOLLI 3(2)3(2)5 (FA 50º)  (0.2 mmol/kg of gadobutrol) |  | 24±6  (n=23) |  | 24±4  (n=69) | 0 |
| Treibel(34) | *shMOLLI  (0.1 mmol/kg of gadoterate meglumine) | 26±2  (n=50) |  | 27± 3  (n=46) |  | 0.5 |
| **Hypertrophic cardiomyopathy** | | **Native T1 (ms)** | | | |  |
| Puntmann(30) | MOLLI 3(2)3(2)5 (FA 50º) |  | 1070±55 (n=30) |  | 1254±43 (n=25) | 3.7 |
| Dass(16) | *shMOLLI |  | 1178±13 (n=12) |  | 1209±28 (n=28) | 1.4 |
| Hinojar(33) | MOLLI 3(2)3(2)5 (FA 50º) |  | 1044±18 (n=23) |  | 1169±41 (n=95) | 3.9 |
|  |  | **Post-contrast T1** | | | |  |
| Puntmann(30) | MOLLI 3(2)3(2)5 (FA 50º)  (0.2 mmol/kg of gadobutrol) |  | 440±58 (n=30) |  | 363±63 (n=25) | 1.3 |
| Hinojar(33) | MOLLI 3(2)3(2)5 (FA 50º)  (0.2 mmol/kg of gadobutrol) |  | 446±70 (n=23) |  | 379±47 (n=95) | 1.3 |
| Ellims(35) | VAST (FA 25°)  (0.2 mmol/kg gadopentetate dimeglumine) | 545±49  (n=25) |  | 483+83 (n=139) |  | 0.9 |
| Ellims(36) | VAST (FA 25°)  (0.2 mmol/kg gadopentetate dimeglumine) | 561 ± 47  (n=25) |  | 498 ± 80 (n=76) |  | 0.96 |
|  |  | **ECV (%)** | | | |  |
| Puntmann(30) | MOLLI 3(2)3(2)5 (FA 50º)  (0.2 mmol/kg of gadobutrol) |  | 27±9 (n=30) |  | 41±12  (n=25) | 1.3 |
| Hinojar(33) | MOLLI 3(2)3(2)5 (FA 50º)  (0.2 mmol/kg of gadobutrol) |  | 24±6 (n=23) |  | 31±6 (n=95) | 1.2 |
| Ho CY(37) | Look-Locker  (0.15 mmol/kg gadopentetate dimeglumine) | 27±1  (n=11) |  | 36±1  (n=37) |  | 9 |
| **HCM G+ relatives** | | **Native T1 (ms)** | | | |  |
| Hinojar(33) | MOLLI 3(2)3(2)5 (FA 50º) |  | 1044±18 (n=23) |  | 1105±17 (n=23) | 3.4 |
|  |  | **Postcontrast T1 (ms)** | | | |  |
| Hinojar(33) | MOLLI 3(2)3(2)5 (FA 50º)  (0.2 mmol/kg of gadobutrol) |  | 446±70 (n=23) |  | 434±67 (n=23) | 1.7 |
|  | | **ECV%** | | | |  |
| Ho CY(37) | Look-Locker  (0.15 mmol/kg gadopentetate dimeglumine) | 27±1  (n=11) |  | 33±1  (n=29) |  | 6 |
| Hinojar(33) | MOLLI 3(2)3(2)5 (FA 50º)  (0.2 mmol/kg of gadobutrol) |  | 24±6 (n=23) |  | 25±4 (n=23) | 2.0 |
| **Anderson-Fabry disease** | | **Native T1 (ms)** | | | |  |
| Sado(38) | *shMOLLI | 968±32  (n=67) |  | 882±47  (n=44) |  | 2.3 |
| Pica(22) | *shMOLLI | 968±32  (n=63) |  | 853±50  (n=63) |  | 2.7 |
| **Iron overload** | | **Native T1 (ms)** | | | |  |
| Sado(39) | *shMOLLI  (0.1 mmol/kg gadoterate meglumine) | 968±32  (n=67) |  | 827±135 (n=88) |  | 1.4 |
|  | | | | | | |
| **Acute viral myocarditis** | | **Native T1 (ms)** | | | |  |
| Hinojar(40) | MOLLI 3(2)3(2)5 (FA 50º) | 940±20  (n=18) | 1045±23 (n=22) | 1064±37 (n=23) | 1189±52 (n=38) | 3.7/3.4 |
| Ferreira(41) | *shMOLLI | 946±23  (n=45) |  | 1010±65 (n=50) |  | 1.4 |
|  |  | **Post contrast T1 (ms)** | | | |  |
| Hinojar(40) | MOLLI 3(2)3(2)5 (FA 50º)  (0.2 mmol/kg of gadobutrol)  Acute (N=61)  Chronic (N=67) | (n=18)  422±68 | 442±68 | (n=23)  373±42  383±43 | (n=38)  397±62  426±73 | 0.5/0.3  0.4/0.2 |
| **Chronic viral myocarditis** | | **Native T1 (ms)** | | | |  |
| Radunski(42) | MOLLI 3(3)5 (FA 35°) | 1051±37  (n=21) |  | 1098±57 (n=114) |  | 0.9 |
| Hinojar(40) | MOLLI 3(2)3(2)5 (FA 50º) | 940±20  (n=18) | 1045±23 (n=22) | 995±19  (n=33) | 1099±22 (n=34) | 2.2/2.2 |
| Bohnen(43) | MOLLI 3(3)5 (FA 35°) | / |  | 1128±~47  (n=31) |  |  |
|  |  | **Postcontrast T1 (ms)** | | | |  |
| Radunski(42) | MOLLI 3(3)5 (FA 35°)  (0.075 mmol/kg gadobenate dimeglumine) | 579±45  (n=21) |  | 555±~61  (n=114) |  | 0.4 |
| Hinojar(40) | MOLLI 3(2)3(2)5 (FA 50º)  (0.2 mmol/kg of gadobutrol) | 422±68  (n=18) | 442±68 (n=22) | 383±43  (n=33) | 426±73 (n=34) | 0.8/0.3 |
| Bohnen(43) | MOLLI 3(3)5 (FA 35°)  (0.075 mmol/kg gadobenate dimeglumine) |  |  | 572±~57  (n=31) |  |  |
|  |  | **ECV (%)** | | | |  |
| Radunski(42) | MOLLI 3(3)5 (FA 35°)  (0.075 mmol/kg gadobenate dimeglumine) | 25±3  (n=21) |  | 31±~4  (n=114) |  | 1.7 |
| Bohnen(43) | MOLLI 3(3)5 (FA 35°)  (0.075 mmol/kg gadobenate dimeglumine) |  |  | 31±~6  (n=31) |  |  |
| **Systemic inflammatory diseases** | | **Native T1 (ms)** | | | |  |
| Puntmann(44) | MOLLI 3(2)3(2)5 (FA 50º) |  | 1056±27 (n=21) |  | 1152±46 (n=33) | 2.5 |
| Hinojar (45) | MOLLI 3(2)3(2)5 (FA 50º) |  | 1057 ± 23  (=n46) |  | 1176 ± 55  (n=76) | 2.8 |
| Ntusi(46) | *shMOLLI | 961±18  (n=39) |  | 973±27 (n=39) |  | 0.5 |
| Holloway(47) | *shMOLLI | 962±18  (n=39) |  | 965±~35 (n=129) |  | 0.8 |
|  |  | **Postcontrast T1 (ms)** | | | |  |
| Puntmann(44) | MOLLI 3(2)3(2)5 (FA 50º)  (0.2 mmol/kg of gadobutrol) |  | 454±53  (n=21) |  | 411±61 (n=33) | 0.7 |
| Ntusi(46) | *shMOLLI  (0.15 mmol/kg gadopentetate dimeglumine) | 468±32  (n=39) |  | 450±40  (n=39) |  | 0.5 |
|  |  | **ECV (%)** | | | |  |
| Puntmann(44) | MOLLI 3(2)3(2)5 (FA 50º)  (0.2 mmol/kg of gadobutrol) |  | 26±5  (n=21) |  | 30±6  (n=33) | 0.7 |
| Ntusi(46) | *shMOLLI  (0.15 mmol/kg gadopentetate dimeglumine) | 28±2  (n=39) |  | 30±3  (n=39) |  | 0.6 |
| **Chemotherapy** | | **ECV (%)** | | | |  |
| Neilan(48) | Look-Locker  (0.15 mmol/kg gadodiamide) | 28±2  (n=15) |  | 30±3  (n=42) |  | 0.8 |
|  | |  |  |  |  |  |
| **Congenital heart disease** | | **ECV (%)** | | | |  |
| Broberg(49)** | Look-Locker  (0.15 mmol/kg gadodiamide) | 25±2  (n=14) |  | 32±5  (n=40) |  | 1.8 |
| Dusenbery(50) | Look-Locker  (0.15-0.2mmol/kg gadopentetate dimeglumine) | 25±4  (n=27) |  | 27±8  (n=35) |  | 0.3 |
| **Acute myocardial infarction** | | **Native T1 (ms)** | | | |  |
| Messroghli(51) | MOLLI 3’3’5 (FA 50°)  Remote myocardium  Infarcted myocardium | (n=15)  982±46(19) |  | (n=24)  1011±66  1197±76 |  | 0.5  3.4 |
| Dall’Armellina(52) | *shMOLLI  Remote myocardium  Infarcted myocardium |  | (n=10)  1166±60(58) |  | (n=41)  1196±56  1257±97 | 0.5  1.1 |
| **Chronic myocardial infarction** | | **Native T1 (ms)** | | | |  |
| Messroghli(51) | MOLLI 3’3’5 (FA 50°)  Remote myocardium  Infarcted myocardium | 982±46(19) |  | (n=24)  987±34  1060±61 |  | 0.1  1.4 |
| Iles(1) | VAST (FA 25°)  Remote myocardium | 975±62  (n=20) |  | 874±74  (n=25) |  | 1.5 |
| Puntmann(31) | MOLLI 3(2)3(2)5 (FA 50º)  Remote myocardium |  | 1055±22 (n=47) |  | 1145±37 (n=91) | 3.0 |
|  |  | **Postcontrast T1 (ms)** | | | |  |
| Iles(1) | VAST (FA 25°)  (0.2 mmol/kg gadopentetate dimeglumine)  Remote myocardium | 543±32  (n=20) |  | 383±17 (n=25) |  | 6.2 |
|  |  | **ECV (%)** | | | |  |
| Ugander(32)  LGE-  LGE+ | MOLLI 3(3)5 (FA 35°)  (0.15 mmol/kg gadopentetate dimeglumine) | (n=11)  25±3 |  | (n=36)  27±3  51±8 |  | 0.6  4.3 |

**Table 3c-i.6. Outcome studies for all-cause mortality (A) and composite cardiac/heart failure (B) endpoints.** §All comers – symptomatic patients referred to a clinical CMR as a part of routine work-up (i.e. ischemic and non-ischemic cardiomyopathies), in analyses these studies typically excluded hypertrophic cardiomyopathy cardiac amyloidosis, Anderson-Fabry disease, adult congenital heart disease, see Methods of respective studies for detail. §§Composite heart failure endpoints may vary between the studies – see annotations for details. HFpEF – heart failure with preserved EF; DCM – dilated cardiomyopathy; follow up is expressed as average/ interquartile range/standard deviation (SD); _b_ – binary variable; tertile –lower-mid tertile vs. upper tertile; NR- Not reported. Order of studies is by the year of publication. P<0.05 is considered significant: *-<0.05; **<0.01. ¥ shMOLLI is a MOLLI (5(1)1(1)1 (FA 35°) variant utilizing a conditional reconstruction algorithm.

| **A. All-cause mortality** | |  |  |  |  | **Univariate** | **Multivariate** |
| --- | --- | --- | --- | --- | --- | --- | --- |
|  | **Study type** | **Patient population (n),**  **follow-up (months)** | **Sequence** | **Field Strength**  **(Tesla)** | **Myocardial T1 index** | **HR (95%CI), p-value (***-<0.05; **<0.01) | **HR (95%CI), p-value (***-<0.05; **<0.01) |
| Wong(53) | Observational, single centre | All-comers§  n=793,  9.6 (6-14.4) | Native: MOLLI 5(3)1 (FA35°),  Postcontrast: MOLLI 4(1)2(1)1 (FA 35°)  (0.2-mmol/kg gadoteridol) | 1.5 | ECV (%) | 1.27 (1.18-1.36)** | 1.18(1.09-1.29)** |
| Banypersad (27) | Observational, single centre | Amyloidosis  n=100,  23 | ¥ shMOLLI  (0.1 mmol/kg gadoterate meglumine) | 1.5 | Native T1_b_(>1044 ms)  ECV_b_(>0.45) | 5.39 (1.24-23.4)*  3.84(1.53-9.61)** |  |
| Schelbert(54) | Observational, single centre | All-comers§  n=1172,  20.4 (12-29) | Native: MOLLI 5(3)2 (FA 35°),  Postcontrast: MOLLI 4(1)2(1)1 (FA 35°)  (0.2-mmol/kg gadoteridol) | 1.5 | ECV (%)  ECV_b_ (>28%) | 1.23(1.15-1.3)**  3.60(2.07-6.26)** | 1.14(1.06-1.27)** |
| Puntmann(55) | Observational multicentre | DCM,  n=637,  22 (19-25) | MOLLI 3(2)3(2)5 (FA 50º) (0.2 mmol/kg of gadobutrol) | 1.5/3.0 | Native T1 (10 ms)  ECV (%) | 1.1(1.06-1.15)**  1.09(1.05-1.14)** | 1.1 (1.07–1.17)** |
|  | | | | | Native T1_b_(>2SD) | 5.2(2.4–14.6)** | 5.4(2.5–15.2)** |
|  |  |  |  |  | Native T1_b_(tertile) | 9.1(3.8-19.2)** | 10.5(3.8–19.2)** |

| **B. Composite Cardiac and Heart Failure Endpoints§§** | | | |  |  |  |  |
| --- | --- | --- | --- | --- | --- | --- | --- |
| Mascherbauer(9) | Observational, single centre | HFpEF  n=100,  23 (±5) | FLASH-IR  (0.1 mmol/kg of gadobutrol) | 1.5 | Postcontrast T1 (ms) | 0.99 (0.98-0.99)* |  |
| Schelbert(54) | Observational, single centre | All-comers§  n=1172,  20.4 (12-29) | Native: MOLLI 5(3)1 (FA 35°),  Postcontrast: MOLLI 4(1)2(1)1 (FA 35°)  (0.2-mmol/kg intravenous gadoteridol) | 1.5 | ECV (%)  ECV_b_ (>28%) | 1.3(1.19-1.4)**  5.25(2.57-10.7)** | 1.14(1.06-1.27) |
| Kammerlander (2) | Observational, single centre | All comers§  n=473  13(±9) | MOLLI 5(3)3 (FA 35°) for native acquisition  MOLLI 4(1)3(1)2(FA 35°) for postcontrast acquisition (0.1 mmol/kg of gadobutrol) |  | ECV (%) | 1.11(1.05–1.17)** | 1.09(1.03–1.16)** |
| Puntmann(55) | Observational multicentre | DCM  n=637  22 (19-25) | MOLLI 3(2)3(2)5 (FA 50º)  (0.2 mmol/kg of gadobutrol) | 1.5/3.0 | Native T1 (10 ms)  ECV (per %) | (1.01–1.10)**  1.05 (1.02–1.08)** | 1.07 (1.05–1.1)** |
|  | | | | | Native T1_b_(>2SD) | 4.7 (2.5 – 8.7)** | 4.8 (2.6–9.1)** |
|  |  |  |  |  | Native T1_b_(tertile) | 4.8 (2.9-8.0)** | 4.7 (2.8–8.0)** |

§§Composite cardiac and heart failure outcomes definitions:

(9) hospitalization for HF or death from cardiovascular causes;

(54) HF hospitalization;

(2)cardiac event (hospitalizations for cardiovascular reasons, cardiac deaths), multivariate analyses were performed for imaging parameters separately; in the combined multivariate analysis, clinical and imaging parameters, age, atrial fibrillation, previous CABG, and RV size were identified as the independent predictors.

(55)(death due to HF and HF hospitalization)

**References**

1. Iles L, Pfluger H, Phrommintikul A, Cherayath J, Aksit P, Gupta SN, Evaluation of Diffuse Myocardial Fibrosis in Heart Failure With Cardiac Magnetic Resonance Contrast-Enhanced T1 Mapping. Journal of the American College of Cardiology. 2008 Nov;52(19):1574–80.

2. Kammerlander AA, Marzluf BA, Zotter-Tufaro C, Aschauer S, Duca F, Bachmann A, T1 Mapping by CMR Imaging. JACC: Cardiovascular Imaging. 2016 Jan;9(1):14–23.

3. Bull S, White SK, Piechnik SK, Flett AS, Ferreira VM, Loudon M, Human non-contrast T1 values and correlation with histology in diffuse fibrosis. Heart. 2013 Jun 5;99(13):932–7.

4. Iles LM, Ellims AH, Llewellyn H, Hare JL, Kaye DM, McLean CA, Histological validation of cardiac magnetic resonance analysis of regional and diffuse interstitial myocardial fibrosis. European Heart Journal - Cardiovascular Imaging. 2015 Jan 12;16(1):14–22.

5. Miller CA, Naish JH, Bishop P, Coutts G, Clark D, Zhao S, Comprehensive Validation of Cardiovascular Magnetic Resonance Techniques for the Assessment of Myocardial Extracellular Volume. Circulation: Cardiovascular Imaging. 2013 May 21;6(3):373–83.

6. aus dem Siepen F, Buss SJ, Messroghli D, Andre F, Lossnitzer D, Seitz S, T1 mapping in dilated cardiomyopathy with cardiac magnetic resonance: quantification of diffuse myocardial fibrosis and comparison with endomyocardial biopsy. European Heart Journal - Cardiovascular Imaging. 2015 Jan 27;16(2):210–6.

7. de Meester de Ravenstein C, Bouzin C, Lazam S, Boulif J, Amzulescu M, Melchior J, Histological Validation of measurement of diffuse interstitial myocardial fibrosis by myocardial extravascular volume fraction from Modified Look-Locker imaging (MOLLI) T1 mapping at 3 T. Journal of Cardiovascular Magnetic Resonance. 2015 Jun 11;17(1):1268.

8. Sibley CT, Noureldin RA, Gai N, Nacif MS, Liu S, Turkbey EB, T1 Mapping in Cardiomyopathy at Cardiac MR: Comparison with Endomyocardial Biopsy. Radiology. 2012 Dec;265(3):724–32.

9. Mascherbauer J, Marzluf BA, Tufaro C, Pfaffenberger S, Graf A, Wexberg P, Cardiac Magnetic Resonance Postcontrast T1 Time Is Associated With Outcome in Patients With Heart Failure and Preserved Ejection Fraction. Circulation: Cardiovascular Imaging. 2013 Nov 19;6(6):1056–65.

10. Flett AS, Hayward MP, Ashworth MT, Hansen MS, Taylor AM, Elliott PM, Equilibrium Contrast Cardiovascular Magnetic Resonance for the Measurement of Diffuse Myocardial Fibrosis: Preliminary Validation in Humans. Circulation. 2010 Jul 12;122(2):138–44.

11. Fontana M, White SK, Banypersad SM, Sado DM, Maestrini V, Flett AS, Comparison of T1 mapping techniques for ECV quantification. Histological validation and reproducibility of ShMOLLI versus multibreath-hold T1 quantification equilibrium contrast CMR. Journal of Cardiovascular Magnetic Resonance. 2012;14(1):88.

12. White SK, Sado DM, Fontana M, Banypersad SM, Maestrini V, Flett AS, T1 Mapping for Myocardial Extracellular Volume Measurement by CMR. JACC: Cardiovascular Imaging. 2013 Sep;6(9):955–62.

13. Lee S-P, Lee W, Lee JM, Park E-A, Kim H-K, Kim Y-J, Assessment of Diffuse Myocardial Fibrosis by Using MR Imaging in Asymptomatic Patients with Aortic Stenosis. Radiology. 2015 Feb;274(2):359–69.

14. Dabir D, Child N, Kalra A, Rogers T, Gebker R, Jabbour A, Reference values for healthy human myocardium using a T1 mapping methodology: results from the International T1 Multicenter cardiovascular magnetic resonance study. Journal of Cardiovascular Magnetic Resonance. 2014 Oct 21;16(1):34.

15. Ferreira VM, Piechnik SK, Dall'Armellina E, Karamitsos TD, Francis JM, Choudhury RP, et al. Non-contrast T1-mapping detects acute myocardial edema with high diagnostic accuracy: a comparison to T2-weighted cardiovascular magnetic resonance. J Cardiovasc Magn Reson. 2012 Jun 21;14:42.

16. Dass S, Suttie JJ, Piechnik SK, Ferreira VM, Holloway CJ, Banerjee R, Myocardial Tissue Characterization Using Magnetic Resonance Noncontrast T1 Mapping in Hypertrophic and Dilated Cardiomyopathy. Circulation: Cardiovascular Imaging. 2012 Nov 20;5(6):726–33.

17. Rogers T, Dabir D, Mahmoud I, Voigt T, Schaeffter T, Nagel E, Standardization of T1 measurements with MOLLI in differentiation between health and disease – the ConSept study. Journal of Cardiovascular Magnetic Resonance. 2013;15(1):78.

18. Knobelsdorff-Brenkenhoff von F, Prothmann M, Dieringer MA, Wassmuth R, Greiser A, Schwenke C, Myocardial T1 and T2 mapping at 3 T: reference values, influencing factors and implications. Journal of Cardiovascular Magnetic Resonance. 2013;15(1):53.

19. Liu S, Han J, Nacif MS, Jones J, Kawel N, Kellman P, Diffuse myocardial fibrosis evaluation using cardiac magnetic resonance T1 mapping: sample size considerations for clinical trials. Journal of Cardiovascular Magnetic Resonance. 2012;14(1):90.

20. Messroghli DR, Plein S, Higgins DM, Walters K, Jones TR, Ridgway JP, Human Myocardium: Single-Breath-hold MR T1 Mapping with High Spatial Resolution—Reproducibility Study. Radiology. 2006 Mar;238(3):1004–12.

21. Singh A, Horsfield MA, Bekele S, Khan JN, Greiser A, McCann GP. Myocardial T1 and extracellular volume fraction measurement in asymptomatic patients with aortic stenosis: reproducibility and comparison with age-matched controls. European Heart Journal - Cardiovascular Imaging. 2015 Jun 11;16(7):763–70.

22. Pica S, Sado DM, Maestrini V, Fontana M, White SK, Treibel T, Reproducibility of native myocardial T1 mapping in the assessment of Fabry disease and its role in early detection of cardiac involvement by cardiovascular magnetic resonance. Journal of Cardiovascular Magnetic Resonance. 2014 Dec 5;16(1):326.

23. Weingärtner S, Meßner NM, Budjan J, Lossnitzer D, Mattler U, Papavassiliu T, Myocardial T1-mapping at 3T using saturation-recovery: reference values, precision and comparison with MOLLI. Journal of Cardiovascular Magnetic Resonance. BioMed Central; 2016 Nov 18;18(1):84.

24. Piechnik SK, Ferreira VM, Lewandowski AJ, Ntusi NA, Banerjee R, Holloway C, Normal variation of magnetic resonance T1 relaxation times in the human population at 1.5 T using ShMOLLI. Journal of Cardiovascular Magnetic Resonance. 2013;15(1):13.

25. Liu C-Y, Liu Y-C, Wu C, Armstrong A, Volpe GJ, van der Geest RJ, Evaluation of Age-Related Interstitial Myocardial Fibrosis With Cardiac Magnetic Resonance Contrast-Enhanced T1 Mapping. Journal of the American College of Cardiology. 2013 Oct;62(14):1280–7.

26. Fontana M, Banypersad SM, Treibel TA, Maestrini V, Sado DM, White SK, Native T1 Mapping in Transthyretin Amyloidosis. JACC: Cardiovascular Imaging. 2014 Feb;7(2):157–65.

27. Banypersad SM, Fontana M, Maestrini V, Sado DM, Captur G, Petrie A, T1 mapping and survival in systemic light-chain amyloidosis. European Heart Journal. 2015 Jan 21;36(4):244–51.

28. Karamitsos TD, Piechnik SK, Banypersad SM, Fontana M, Ntusi NB, Ferreira VM, Noncontrast T1 Mapping for the Diagnosis of Cardiac Amyloidosis. JACC: Cardiovascular Imaging. 2013 Apr;6(4):488–97.

29. Mahmod M1, Piechnik SK, Levelt E, Ferreira VM, Francis JM, Lewis A, et al. Adenosine stress native T1 mapping in severe aortic stenosis: evidence for a role of the intravascular compartment on myocardial T1 values. J Cardiovasc Magn Reson. 2014 Nov 20;16:92.

30. Puntmann VO, Voigt T, Chen Z, Mayr M, Karim R, Rhode K, Native T1 Mapping in Differentiation of Normal Myocardium From Diffuse Disease in Hypertrophic and Dilated Cardiomyopathy. JACC: Cardiovascular Imaging. 2013 Apr;6(4):475–84.

31. Puntmann VO, Arroyo Ucar E, Hinojar Baydes R, Ngah NB, Kuo YS, Dabir D, Aortic Stiffness and Interstitial Myocardial Fibrosis by Native T1 Are Independently Associated With Left Ventricular Remodeling in Patients With Dilated Cardiomyopathy. Hypertension. 2014 Sep 10;64(4):762–8.

32. Ugander M, Oki AJ, Hsu LY, Kellman P, Greiser A, Aletras AH, Extracellular volume imaging by magnetic resonance imaging provides insights into overt and sub-clinical myocardial pathology. European Heart Journal. 2012 May 14;33(10):1268–78.

33. Hinojar R, Varma N, Child N, Goodman B, Jabbour A, Yu C-Y, T1 Mapping in Discrimination of Hypertrophic Phenotypes: Hypertensive Heart Disease and Hypertrophic CardiomyopathyCLINICAL PERSPECTIVE. Circulation: Cardiovascular Imaging. 2015 Dec 14;8(12):e003285.

34. Treibel TA, Zemrak F, Sado DM, Banypersad SM, White SK, Maestrini V, Extracellular volume quantification in isolated hypertension - changes at the detectable limits? Journal of Cardiovascular Magnetic Resonance. 2015 Aug 12;17(1):1031.

35. Ellims AH, Iles LM, Ling L-H, Hare JL, Kaye DM, Taylor AJ. Diffuse myocardial fibrosis in hypertrophic cardiomyopathy can be identified by cardiovascular magnetic resonance, and is associated with left ventricular diastolic dysfunction. Journal of Cardiovascular Magnetic Resonance. 2012;14(1):76.

36. Ellims AH, Iles LM, Ling LH, Chong B, Macciocca I, Slavin GS, A comprehensive evaluation of myocardial fibrosis in hypertrophic cardiomyopathy with cardiac magnetic resonance imaging: linking genotype with fibrotic phenotype. European Heart Journal - Cardiovascular Imaging. 2014 Sep 19;15(10):1108–16.

37. Ho CY, Abbasi SA, Neilan TG, Shah RV, Chen Y, Heydari B, T1 Measurements Identify Extracellular Volume Expansion in Hypertrophic Cardiomyopathy Sarcomere Mutation Carriers With and Without Left Ventricular Hypertrophy. Circulation: Cardiovascular Imaging. 2013 May 21;6(3):415–22.

38. Sado DM, White SK, Piechnik SK, Banypersad SM, Treibel T, Captur G, Identification and Assessment of Anderson-Fabry Disease by Cardiovascular Magnetic Resonance Noncontrast Myocardial T1 Mapping. Circulation: Cardiovascular Imaging. 2013 May 21;6(3):392–8.

39. Sado DM, Maestrini V, Piechnik SK, Banypersad SM, White SK, Flett AS, Noncontrast myocardial T1mapping using cardiovascular magnetic resonance for iron overload. J Magn Reson Imaging. 2014 Aug 8;41(6):1505–11.

40. Hinojar R, Foote L, Arroyo Ucar E, Jackson T, Jabbour A, Yu C-Y, Native T1 in Discrimination of Acute and Convalescent Stages in Patients With Clinical Diagnosis of Myocarditis. JACC: Cardiovascular Imaging. 2015 Jan;8(1):37–46.

41. Ferreira VM, Piechnik SK, Dall'Armellina E, Karamitsos TD, Francis JM, Ntusi N, T1 Mapping for the Diagnosis of Acute Myocarditis Using CMR. JACC: Cardiovascular Imaging. 2013 Oct;6(10):1048–58.

42. Radunski UK, Lund GK, Stehning C, Schnackenburg B, Bohnen S, Adam G, CMR in Patients With Severe Myocarditis. JACC: Cardiovascular Imaging. 2014 Jul;7(7):667–75.

43. Bohnen S, Radunski UK, Lund GK, Kandolf R, Stehning C, Schnackenburg B, Performance of T1 and T2 Mapping Cardiovascular Magnetic Resonance to Detect Active Myocarditis in Patients With Recent-Onset Heart Failure. Circulation: Cardiovascular Imaging. 2015 May 19;8(6):e003073–3.

44. Puntmann VO, D'Cruz D, Smith Z, Pastor A, Choong P, Voigt T, Native Myocardial T1 Mapping by Cardiovascular Magnetic Resonance Imaging in Subclinical Cardiomyopathy in Patients With Systemic Lupus Erythematosus. Circulation: Cardiovascular Imaging. 2013 Mar 19;6(2):295–301.

45. Hinojar R, Foote L, Sangle S, Marber M, Mayr M, Carr-White G, Native T1 and T2 mapping by CMR in lupus myocarditis: Disease recognition and response to treatment. Int J Cardiol. 2016 Nov;222:717–26.

46. Ntusi NA, Piechnik SK, Francis JM, Ferreira VM, Matthews PM, Robson MD, Diffuse myocardial fibrosis is associated with impaired myocardial strain and disease activity in rheumatoid arthritis: a cardiovascular magnetic resonance study. Journal of Cardiovascular Magnetic Resonance. 2014;16(Suppl 1):P292.

47. Holloway CJ, Ntusi N, Suttie J, Mahmod M, Wainwright E, Clutton G, Comprehensive Cardiac Magnetic Resonance Imaging and Spectroscopy Reveal a High Burden of Myocardial Disease in HIV Patients. Circulation. 2013 Aug 19;128(8):814–22.

48. Neilan TG, Pena-Herrera D, Coelho-Filho OR, Jerosch-Herold M, Moslehi J, Kwong R. Left ventricular mass by cardiac magnetic resonance imaging and adverse cardiovascular outcomes in patients treated with anthracycline-based chemotherapy. Journal of Cardiovascular Magnetic Resonance. 2012;14(Suppl 1):O30.

49. Broberg CS, Chugh SS, Conklin C, Sahn DJ, Jerosch-Herold M. Quantification of Diffuse Myocardial Fibrosis and Its Association With Myocardial Dysfunction in Congenital Heart Disease. Circulation: Cardiovascular Imaging. 2010 Nov 16;3(6):727–34.

50. Dusenbery SM, Jerosch-Herold M, Rickers C, Colan SD, Geva T, Newburger JW, Myocardial Extracellular Remodeling Is Associated With Ventricular Diastolic Dysfunction in Children and Young Adults With Congenital Aortic Stenosis. Journal of the American College of Cardiology. 2014 May;63(17):1778–85.

51. Messroghli DR, Walters K, Plein S, Sparrow P, Friedrich MG, Ridgway JP, MyocardialT1 mapping: Application to patients with acute and chronic myocardial infarction. Magn Reson Med. 2007;58(1):34–40.

52. Dall'Armellina E, Ferreira VM, Kharbanda RK, Prendergast B, Piechnik SK, Robson MD, Diagnostic Value of Pre-Contrast T1 Mapping in Acute and Chronic Myocardial Infarction. JACC: Cardiovascular Imaging. 2013 Jun;6(6):739–42.

53. Wong TC, Piehler K, Meier CG, Testa SM, Klock AM, Aneizi AA, Association Between Extracellular Matrix Expansion Quantified by Cardiovascular Magnetic Resonance and Short-Term Mortality. Circulation. 2012 Sep 4;126(10):1206–16.

54. Schelbert EB, Piehler KM, Zareba KM, Moon JC, Ugander M, Messroghli DR, Myocardial Fibrosis Quantified by Extracellular Volume Is Associated With Subsequent Hospitalization for Heart Failure, Death, or Both Across the Spectrum of Ejection Fraction and Heart Failure Stage. J Am Heart Assoc. 2015 Dec 18;4(12):e002613.

55. Puntmann VO, Carr-White G, Jabbour A, Yu C-Y, Gebker R, Kelle S, T1-Mapping and Outcome in Nonischemic Cardiomyopathy. JACC: Cardiovascular Imaging. 2016 Jan;9(1):40–50.
