# Supplementary material for: Society for Cardiovascular Magnetic Resonance (SCMR) expert consensus for CMR imaging endpoints in clinical research: part I - analytical validation and clinical qualification
Source: J Cardiovasc Magn Reson. 2018 Sep 20;20:67. doi: 10.1186/s12968-018-0484-5 (PMC6147157; doi:10.1186/s12968-018-0484-5)
Supplement: Supplementary file 4 — T2 weighted imaging. Table 3b-ii.1: Validation studies with T2 weighted imaging (T2W). Table 3b-ii.2: Correlations with other relevant parameters for T2W-AAR. Table 3b-ii.3: Proof of concept studies using T2W imaging in health and disease. Table 3b-ii.4: Outcome studies for all-cause mortality or major adverse cardiac events (MACE). (DOCX 53 kb) [file 12968_2018_484_MOESM4_ESM.docx]

## T2 weighted imaging

**Table 3b-ii.1: Validation studies with T2 weighted imaging (T2W**). IR – ischaemia-reperfusion model. T2-TSE - double-inversion black blood fast-spin-echo, SI – signal intensity, ECV – extracellular volume, AAR – area-at-risk, TTC -Triphenyltetrazolim chloride-stained, T2W(STIR) - triple-inversion black blood (short tau) fast-spin-echo, CNR – contrast to noise ratio, T2-SFFP – steady-state free precession.. *Histological diagnosis of myocarditis include histological, immunohistological, and molecular pathological analyses diagnosing myocardial inflammation and viral infections [1]. Edema ratio described in [2].

|  | **N** | **Disease model** | **Validation approach** | **Sequence** | **Time-points** | **Correlation/Agreement or Accuracy(95%CI)** | | |
| --- | --- | --- | --- | --- | --- | --- | --- | --- |
|  | | | |  |  | | | |
| **Animal studies** | | |  |  |  | **T2W** | **R** | **P value** |
| **Higgins** [3] | 8 | IR (Dogs) | Water content | T2-TSE | 24h | T2W SI | 0.90 | <0.001 |
| **Garcia-Dorado** [4] | 21 | IR (ex vivo pig heart) | Water content | T2-TSE | (time-point not available) | T2 time | 0.76 | p< 0.001 for all |
|  |  |  |  |  |  | T2W SI | 0.83 |  |
|  |  |  | Histological ECV |  |  | T2 time | 0.58 |  |
|  |  |  |  |  |  | T2W SI | 0.59 |  |
|  |  |  | Histology (Fluorescein) |  |  | AAR- T2W (%LV) | 0.96 |  |
| **Aletras** [5] | 17 | IR (Dogs) | Microspheres (TTC staining of infarction) | T2-TSE | 2 days after the  coronary artery occlusion 90min /reperfusion | AAR- T2W (%LV) | 0.84 | <0.001 |
| **Tilak** [6] | 14 | IR (Dogs) | First pass contrast enhanced perfusion | T2-TSE | Day 0 and 2 after the  coronary artery occlusion 90min /no reperfusion | AAR- T2W (%LV) | 0.91 | P<0.001 |
| **Abdel-Aty**[7] | 15 | IR (dogs) | Water content | T2-STIR |  | T2-CNR | 0.77 | 0.04 |
| **Payne** [8] | 15 | IR (pig) | Histological haemorrhage | T2-SSFP | 0, 3, 10, 60 days | AAR- T2W (%LV) | Sens 98(94-100)  Spec 90(83-98) | |
| **Fernandez-Jimenez** [9] | 25 | IR (pig) | Water content |  | T2-TSE | T2 time | 0.87 | |
|  |  |  |  |  | T2 mapping | T2 time | 0.85 | |
| **Human studies** | | |  |  |  |  | | |
|  | | |  |  |  |  | | |
| **Lurz** [10] | 132 | Myocarditis | Histological diagnosis of myocarditis* | T2-STIR |  | T2W(STIR) – Edema ratio | 59(51-67) | |
|  |  | Acute (n=70) |  |  |  |  | 63(53-76) | |
|  |  | Chronic (n=62) |  |  |  |  | 55(41-66) | |
| **Krieghoff** [11] | 93 | Heart transplant (n=73) | Histological diagnosis of Grade ≥ 1B rejection* | T2-STIR |  | T2W(STIR) – Edema ratio | Sens: 63, Spec: 75 | |
| **Gutberlet** [12] | 49 | Chronic myocarditis | Histological diagnosis of myocarditis* | T2-STIR |  | T2W(STIR) – Edema ratio | 68 | |
| **Francone** [13] | 57 | Acute myocarditis | Histological diagnosis of myocarditis* | T2-STIR |  | T2W(STIR) – Edema ratio | Sens: 27-81 | |

**Table 3b-ii.2. Correlations with other relevant parameters for T2W-AAR.** AMI – acute myocardial infarction, STEMI – ST-elevation myocardial infarction, SPECT – single photon emission computed tomography, ESL-LGE – endocardial surface length.

| **AMI-AAR** |  | |  |  | Correlations/Agreement | |
| --- | --- | --- | --- | --- | --- | --- |
| **Berry** [14] | 50 | AMI | 1.5 | T2 SSFP | Approach-AAR | 0.78 |
|  |  |  |  |  | DUKE Jeopardy | 0.39 |
| **Carlsson** [15] | 16 | STEMI | 1.5 | T2-STIR | SPECT | 0.70 |
| **Wright** [16] | 108 |  |  |  | ESL-LGE | 0.77 |
| **Fuernau** [17] | 197 | STEMI | 1.5 | T2-STIR | Approach-AAR | 0.87 |
|  |  |  |  |  | ESL-LGE | 0.56 |

**Table 3b-ii.3. Proof of concept studies using T2W imaging in health and disease.** Studies included if n>25 subjects per patients’ group. Number of participants per group, mean values (mean±SD, or standard error (SE)) are reported for disease entity, the type of sequence and field strength, including effect size as a measure of dispersion observed in healthy subjects, as well as the Cohen’s d index. The order relates to the order referencing.

|  | **N** | **Disease model** | **Field Strength** | **Sequence** | **Health vs disease** | | |
| --- | --- | --- | --- | --- | --- | --- | --- |
|  | | |  |  | **Controls** | **Patients** | **Cohen D** |
| **Myocarditis** | |  |  |  |  |  |  |
| **Friedrich** [18] | 44 | Acute (suspected) | 1.5 | T2W STIR (body coil) | 1.36±0.2(SE) | 1.6±0.2 (SE) | 0.6 |
| **Abdel-Aty** [19] | 25 | Acute (suspected) | 1.5 | T2W STIR (body coil) | 1.7±0.4 | 2.3±0.4 | 1.5 |
| **Puntmann** [20] | 34 | Acute (suspected) | 1.5 | T2W STIR | 2.5±1.1 | 4.9±2.4 | 1.3 |
| **Mavrogeni** [21] | 71 | Suspected | 1.5 | T2W STIR | 1.57±0.13 | 2.6±0.9 | 1.6 |
| **Mavrogeni** [22] | 32 | H1N1 | 1.5 | T2W STIR | 1.56 ± 0.12 | 1.9 ± 0.16 | 0.81 |
| **Ferreira** [23] | 50 | Acute (suspected) | 1.5 | T2W STIR | 1.56±0.15 | 1.73±0.27 | 0.55 |
| **Radunski** [24] | 104 | Chronic (suspected) | 1.5 | T2W STIR | 2.3(2.1-3.0) | 2.5(2.2-2.9) | 0.44 |
| **Hinojar** [25] | 128 | Acute (suspected, n=61) | 1.5/3.0 | T2W STIR | 1.3 (1.1–1.6) | 2.3 (1.5–3.5) | 1.6 |
|  |  | Chronic (suspected, n=67) |  |  |  | 1.4 (1.1–2.3) | 0.22 |
| **Von Knobelsdorff-Brenkenhoff**[26] | 18 | Acute (suspected) | 1.5 | T2W STIR | 1.6 (1.5–1.7) | 2.2(2.0–2.3) | 3.6 |
| **Systemic inflammatory conditions** | | |  |  |  |  |  |
| **Mavrogeni** [27] | 50 | Lupus | 1.5 | T2W STIR | 1.9±0.1 | 24±0.4 |  |
| **Puntmann** [28] | 33 | Lupus | 3.0 | T2W STIR | 1.7±0.5 | 1.9±0.7 | 0.33 |
| **Ntusi** [29] | 55 | Rheumatoid arthritis | 1.5 | T2W STIR | 1.5±0.1 | 1.7±0.3 | 0.89 |
| **Ntusi** [30] | 103 | HIV | 1.5 | T2W STIR | 1.49±0.13 | 1.55±0.23 | 0.32 |
| **Luetkens** [31] | 28 | HIV | 1.5 | T2W STIR | 1.4±0.3 | 1.6±0.3 | 0.67 |

**Table 3b-ii.4** **Outcome studies for all-cause mortality or major adverse cardiac events (MACE)**. PPCI – primary percutaneous coronary intervention, MSI – myocardial salvage index, HR – hazard ratio, NSTEMI – non ST-elevation myocardial infarction.

|  | |  |  |  |  | Univariate | Multivariate |
| --- | --- | --- | --- | --- | --- | --- | --- |
|  | Study type | Patient population (n),  follow-up (months) | Sequence | Field Strength  (Tesla) | Myocardial T1 index | HR (95%CI), p-value) | HR (95%CI), p-value |
| **Eitel** [32] | Observational, single centre | STEMI (PPCI),  48 (27-73), n=208 | T2W-STIR | 1.5 | MSI | 0.95(0.93-0.97) | 0.93(0.91-0.96) |
| **Raman** [33] | Observational, single centre | NSTEMI, 6-months, n=88 | T2W-STIR | 1.5 | Oedema | 4.47(1-20.3) |  |
| **De Waha** [34] | Observational, single centre | STEMI (PPCI),  48 (27-73), n=438 | T2W-STIR | 1.5 | MSI | 0.93(0.92-0.95) | 0.92(0.90-0.95) |

**References**

1. Caforio ALP, Pankuweit S, Arbustini E, Basso C, Gimeno-Blanes J, Felix SB, et al. Current state of knowledge on aetiology, diagnosis, management, and therapy of myocarditis: a position statement of the European Society of Cardiology Working Group on Myocardial and Pericardial Diseases. European Heart Journal. 2013;34:2636–48.

2. Friedrich MG, Sechtem U, Schulz-Menger J, Holmvang G, Alakija P, Cooper LT, et al. Cardiovascular Magnetic Resonance in Myocarditis: A JACC White Paper. Journal of the American College of Cardiology. 2009;53:1475–87.

3. Higgins CB, Herfkens R, Lipton MJ, Sievers R, Sheldon P, Kaufman L, et al. Nuclear magnetic resonance imaging of acute myocardial infarction in dogs: alterations in magnetic relaxation times. Am. J. Cardiol. [Internet]. 1983;52:184–8. Available from: http://eutils.ncbi.nlm.nih.gov/entrez/eutils/elink.fcgi?dbfrom=pubmed&id=6858909&retmode=ref&cmd=prlinks

4. Garcia-Dorado D, Oliveras J, Gili J, Sanz E, Perez-Villa F, Barrabes J, et al. Analysis of myocardial oedema by magnetic resonance imaging early after coronary artery occlusion with or without reperfusion. Cardiovascular Research. 1993;27:1462–9.

5. Aletras AH. Retrospective Determination of the Area at Risk for Reperfused Acute Myocardial Infarction With T2-Weighted Cardiac Magnetic Resonance Imaging: Histopathological and Displacement Encoding With Stimulated Echoes (DENSE) Functional Validations. Circulation [Internet]. American Heart Association, Inc; 2006;113:1865–70. Available from: http://circ.ahajournals.org/cgi/doi/10.1161/CIRCULATIONAHA.105.576025

6. Hor KN, Gottliebson WM, Carson C, Wash E, Cnota J, Fleck R, et al. Comparison of Magnetic Resonance Feature Tracking for Strain Calculation With Harmonic Phase Imaging Analysis. JACC: Cardiovascular Imaging. 2010;3:144–51.

7. Abdel-Aty H. Myocardial Edema Imaging of the Area at Risk in Acute Myocardial Infarction. JACC Cardiovasc Imaging. 2009;2:832–4.

8. Payne AR, Berry C, Kellman P, Anderson R, Hsu LY, Chen MY, et al. Bright-Blood T2-Weighted MRI Has High Diagnostic Accuracy for Myocardial Hemorrhage in Myocardial Infarction: A Preclinical Validation Study in Swine. Circ Cardiovasc Imaging [Internet]. American Heart Association, Inc; 2011;4:738–45. Available from: http://circimaging.ahajournals.org/cgi/doi/10.1161/CIRCIMAGING.111.965095

9. García-Álvarez A, García-Lunar I, Pereda D, Fernández-Jiménez R, Sánchez-González J, Mirelis JG, et al. Association of Myocardial T1-Mapping CMR With Hemodynamics and RV Performance in Pulmonary Hypertension. JACC: Cardiovascular Imaging. 2015;8:76–82.

10. Lurz P, Eitel I, Adam J, Steiner J, Grothoff M, Desch S, et al. Diagnostic Performance of CMR Imaging Compared With EMB in Patients With Suspected Myocarditis. JACC: Cardiovascular Imaging. 2012;5:513–24.

11. Krieghoff C, Barten MJ, Hildebrand L, Grothoff M, Lehmkuhl L, Lücke C, et al. Assessment of sub-clinical acute cellular rejection after heart transplantation: comparison of cardiac magnetic resonance imaging and endomyocardial biopsy. Eur Radiol. Springer Berlin Heidelberg; 2014;24:2360–71.

12. Gutberlet M, Spors B, Thoma T, Bertram H, Denecke T, Felix R, et al. Suspected Chronic Myocarditis at Cardiac MR: Diagnostic Accuracy and Association with Immunohistologically Detected Inflammation and Viral Persistence. Radiology. 2008;246:401–9.

13. Francone M, Chimenti C, Galea N, Scopelliti F, Verardo R, Galea R, et al. CMR sensitivity varies with clinical presentation and extent of cell necrosis in biopsy-proven acute myocarditis. JACC: Cardiovascular Imaging. 2014;7:254–63.

14. Berry C, Kellman P, Mancini C, Chen MY, Bandettini WP, Lowrey T, et al. Magnetic Resonance Imaging Delineates the Ischemic Area at Risk and Myocardial Salvage in Patients With Acute Myocardial Infarction. Circ Cardiovasc Imaging [Internet]. American Heart Association, Inc; 2010;3:527–35. Available from: http://circimaging.ahajournals.org/cgi/doi/10.1161/CIRCIMAGING.109.900761

15. Carlsson M, Töger J, Kanski M, Bloch KM, Ståhlberg F, Heiberg E, et al. Quantification and visualization of cardiovascular 4D velocity mapping accelerated with parallel imaging or k-t BLAST: head to head comparison and validation at 1.5 T and 3 T. J Cardiovasc Magn Reson. BioMed Central Ltd; 2011;13:55.

16. Wright J, Adriaenssens T, Dymarkowski S, Desmet W, Bogaert J. Quantification of myocardial area at risk with T2-weighted CMR: comparison with contrast-enhanced CMR and coronary angiography. JACC: Cardiovascular Imaging. 2009;2:825–31.

17. Fuernau G, Eitel I, Franke V, Hildebrandt L, Meissner J, de Waha S, et al. Myocardium at risk in ST-segment elevation myocardial infarction comparison of T2-weighted edema imaging with the MR-assessed endocardial surface area and validation against angiographic scoring. JACC: Cardiovascular Imaging. 2011;4:967–76.

18. Friedrich MG, Strohm O, Schulz-Menger J, Marciniak H, Luft FC, Dietz R. Contrast media-enhanced magnetic resonance imaging visualizes myocardial changes in the course of viral myocarditis. Circulation. 1998;97:1802–9.

19. Abdel-Aty H, Boyé P, Zagrosek A, Wassmuth R, Kumar A, Messroghli D, et al. Diagnostic Performance of Cardiovascular Magnetic Resonance in Patients With Suspected Acute Myocarditis. Journal of the American College of Cardiology. 2005;45:1815–22.

20. Puntmann VO, Taylor PC, Barr A, Schnackenburg B, Jahnke C, Paetsch I. Towards understanding the phenotypes of myocardial involvement in the presence of self-limiting and sustained systemic inflammation: a magnetic resonance imaging study. Rheumatology. 2010;49:528–35.

21. Mavrogeni S, Spargias C, Bratis C, Kolovou G, Markussis V, Papadopoulou E, et al. Myocarditis as a precipitating factor for heart failure: evaluation and 1-year follow-up using cardiovascular magnetic resonance and endomyocardial biopsy. European Journal of Heart Failure. 2011;13:830–7.

22. Mavrogeni S, Bratis C, Kitsiou A, Kolovou G, Manoussakis MN, Papadopoulou E, et al. CMR assessment of myocarditis in patients with cardiac symptoms during H1N1 viral infection. JACC: Cardiovascular Imaging. 2011;4:307–9.

23. Ferreira VM, Piechnik SK, Dall'Armellina E, Karamitsos TD, Francis JM, Ntusi N, et al. T1 Mapping for the Diagnosis of Acute Myocarditis Using CMR. JACC: Cardiovascular Imaging. 2013;6:1048–58.

24. Radunski UK, Lund GK, Stehning C, Schnackenburg B, Bohnen S, Adam G, et al. CMR in Patients With Severe Myocarditis. JACC: Cardiovascular Imaging. 2014;7:667–75.

25. Hinojar R, Foote L, Arroyo Ucar E, Jackson T, Jabbour A, Yu C-Y, et al. Native T1 in Discrimination of Acute and Convalescent Stages in Patients With Clinical Diagnosis of Myocarditis. JACC: Cardiovascular Imaging. 2015;8:37–46.

26. Knobelsdorff-Brenkenhoff von F, Schüler J, Dogangüzel S, Dieringer MA, Rudolph A, Greiser A, et al. Detection and Monitoring of Acute Myocarditis Applying Quantitative Cardiovascular Magnetic Resonance. Circulation: Cardiovascular Imaging. American Heart Association, Inc; 2017;10:e005242.

27. Mavrogeni S, Bratis K, Markussis V, Spargias C, Papadopoulou E, Papamentzelopoulos S, et al. The diagnostic role of cardiac magnetic resonance imaging in detecting myocardial inflammation in systemic lupus erythematosus. Differentiation from viral myocarditis. Lupus. 2012;22:34–43.

28. Puntmann VO, D'Cruz D, Smith Z, Pastor A, Choong P, Voigt T, et al. Native Myocardial T1 Mapping by Cardiovascular Magnetic Resonance Imaging in Subclinical Cardiomyopathy in Patients With Systemic Lupus Erythematosus. Circulation: Cardiovascular Imaging. 2013;6:295–301.

29. Ntusi NAB, Piechnik SK, Francis JM, Ferreira VM, Matthews PM, Robson MD, et al. Diffuse Myocardial Fibrosis and Inflammation in Rheumatoid Arthritis. JACC: Cardiovascular Imaging. 2015;8:526–36.

30. Ntusi N, O'Dwyer E, Dorrell L, Wainwright E, Piechnik S, Clutton G, et al. HIV-1-Related Cardiovascular Disease Is Associated With Chronic Inflammation, Frequent Pericardial Effusions, and Probable Myocardial Edema. Circulation: Cardiovascular Imaging. American Heart Association, Inc; 2016;9:e004430.

31. Luetkens JA, Doerner J, Schwarze-Zander C, Wasmuth J-C, Boesecke C, Sprinkart AM, et al. Cardiac Magnetic Resonance Reveals Signs of Subclinical Myocardial Inflammation in Asymptomatic HIV-Infected Patients. Circulation: Cardiovascular Imaging. American Heart Association, Inc; 2016;9:e004091.

32. Eitel I, Desch S, Fuernau G, Hildebrand L, Gutberlet M, Schuler G, et al. Prognostic significance and determinants of myocardial salvage assessed by cardiovascular magnetic resonance in acute reperfused myocardial infarction. Journal of the American College of Cardiology. 2010;55:2470–9.

33. Raman SV, Aneja A, Jarjour WN. CMR in inflammatory vasculitis. Journal of Cardiovascular Magnetic Resonance. 2012;14:82.

34. de Waha S, Eitel I, Desch S, Fuernau G, Lurz P, Stiermaier T, et al. Prognosis after ST-elevation myocardial infarction: a study on cardiac magnetic resonance imaging versus clinical routine. Trials [Internet]. BioMed Central; 2014;15:249. Available from: http://trialsjournal.biomedcentral.com/articles/10.1186/1745-6215-15-249
