# Supplementary material for: Society for Cardiovascular Magnetic Resonance (SCMR) expert consensus for CMR imaging endpoints in clinical research: part I - analytical validation and clinical qualification
Source: J Cardiovasc Magn Reson. 2018 Sep 20;20:67. doi: 10.1186/s12968-018-0484-5 (PMC6147157; doi:10.1186/s12968-018-0484-5)
Supplement: Supplementary file 5 — T2 mapping Tables. Table 3c-ii.1: Correlation of T2 mapping indices with histological substrates. Table 3c-ii.2: Correlation of T2 mapping indices with other imaging biomarkers. Table 3c-ii.3: Reproducibility for native T2 using various sequences and field strengths. Studies included if reported interstudy reproducibility. Table 3c-ii.4: Normal values for native T2 reported for different sequences and magnetic fields. Studies included if n>50 subjects. Table 3c-ii.5: Proof of concept studies with T2 indices differentiating between health and disease. Studies included if n>25 per patients’ group (unless the only study published). (DOCX 46 kb) [file 12968_2018_484_MOESM5_ESM.docx]

# T2 mapping Tables

**Table 3c-ii.1: Correlation of T2 mapping indices with histological substrates.** Agreement expressed as linear R^2^ regression index or area under the curve (AUC). MSE – multi spin-echo, T2 GraSE – T2 gradient-spin-echo, EMB – endomyocardial biopsy, LLC – Lake Louise criteria, T2WI – T2 weighted imaging, GRE – global relative enhancement, LGE – late gadolinium enhancement.

|  | **N** | **Population** | **Sequence** | **Histological correlation** | **Agreement (R^2^) or AUC** | |
| --- | --- | --- | --- | --- | --- | --- |
| Fernandez-Jimenez(1) | 20 | Pigs (I/R injury) | MSE | Water content | Native T2 | R^2^=0.75 |
|  |  |  | T2 GraSE | Water content | Native T2 | R^2^=0.73 |
| Carpenter(2) | 12 | ExVivo Hearts | MSE | Iron content | Native R2 (=1/T2) | R^2^=0.786 |
| Bohnen(3) | 31 | HF due to suspected chronic myocarditis | T2 GraSE | EMB criteria for myocarditis | LLC  T1 mapping (MOLLI 3(3)5(FA 35°)  T2 mapping (T2 GraSE) | - T2WI 0.56 - GRE 0.55 - LGE 0.60 - Native T1 0.65 - Postcontrast T1 0.86 - ECV 0.58 - T2 map 0.78 |
| Lurz(4) | 129 | Suspected myocarditis   - acute symptoms <14 - days - chronic symptoms >14 days | T2GraSE | EMB criteria for myocarditis | LLC  T1 mapping (MOLLI 3(3)5(FA 35°)  T2 mapping (T2 GraSE) | Acute symptoms (n=61)   - LLC 0.56 - Native T1 0.82 - ECV 0.75 - T2 map 0.81   Chronic symptoms (n=68)   - LLC 0.53 - Native T1 0.53 - ECV 0.61 - T2 map 0.77 |

**Table 3c-ii.2: Correlation of T2 mapping indices with other imaging biomarkers.** SSFP – steady-state free precession, MI – myocardial infarction (STEMI/NSTEMI), AAR – area-at-risk, SPECT – single proton emission computed tomography, STEMI - ST elevation myocardial infarction, NSTEMI - non-ST elevation myocardial infarction, TTCM: tako-tsubo cardiomyopathy, T2WI-STIR - triple-inversion black blood (short tau) fast-spin-echo.

|  | **N** | **T2 mapping sequence** | **Population** | **Imaging biomaker** | **Outcome/Agreement** |
| --- | --- | --- | --- | --- | --- |
| Bulluck(5) | 18 | T2 SSFP | Acute MI | AAR by T2WI/LGE | AUC=0.86 |
| Park(6) | 20 | T2 SSFP | Acute MI | T2WI | - T2 mapping 95% - T2WI 55% |
| Langhans(7) | 14 | T2 SSFP | Acute MI | AAR by SPECT | R=0.94 (threshold of 60msec) |
| Nassenstein(8) | 29 | T2 SSFP | STEMI/ NSTEMI | T2WI | - T2 map🡪 sensitivity 82%, specificity 94% - T2WI🡪 sensitivity 50.4%, specificity 98% |
| Thavendiranathan (9) | 30 | T2 SSFP | Myocarditis or TTCM | T2WI-STIR | T2 map 🡪 sensitivity 94%, specificity 97% (threshold of 59 msec), |
| Van Heeswijk(10) | 11 | MSE | STEMI | T2WI | r=0.91 |
| Verhaert(11) | 26 | T2 SSFP | Acute MI | T2WI | T2 mapping detected edema more frequently than T2WI in infarcted myocardium (96% vs 67%) |

**Table 3c-ii.3. Reproducibility for native T2 using various sequences and field strengths. Studies included if reported interstudy reproducibility.** Results are reported as Bland-Altman plots: MD±SD and CoV in brackets when available

| **T2 mapping (msec)** | Wassmuth (12) |
| --- | --- |
| **Magnetic field** | 1.5 |
| **N** | 73 |
| **Population** | Controls |
| **Sequence** | T2 SSFP |
| **No of echo images** | 3 |
| **Interobserver V** | 1.6±1.5 |
| **Intraobserver V** | 1.1±1.0 |
| **Interstudy V** | (7.6%) |

**Table 3c-ii.4. Normal values for native T2 reported for different sequences and magnetic fields. Studies included if n>50 subjects.**

Mean native T2 values±SD or 95% CI in single mid-ventricular slice, expressed in ms.

|  | **N** | **Age (years, range)** | **Sequence** | **Native T2** | | |
| --- | --- | --- | --- | --- | --- | --- |
|  |  |  |  | **1.5 T** | **3.0 T** |  |
| Wassmuth (12) | 73 | 35±13 | mSE | 52±5 |  |  |
|  | 73 |  | T2 SSFP | 55±5 |  |  |
| Von Knobelsdorff (13) | 58 | 20-80 | T2 SSFP |  | 45.1 (39.3 – 49.5) |  |
| Boenner (14) | 74 |  | T2 GraSE | Male: 57.5±3.5  Female: 60 ± 3.8 |  |  |

**Table 3c-ii.5. Proof of concept studies with T2 indices differentiating between health and disease. Studies included if n>25 per patients’ group (unless the only study published).** The table reports mean values±SD for each disease entity, sequence type, T2 index, and field strength; includes effect size as a measure of dispersion observed in healthy subjects, as well as the Cohen’s d index. Native T2 values are expressed in ms.

| **Disease model** | **Sequence** | **Health (n)** | | **Disease (n)** | | **Effect Size**  **(Cohen’s d)** |
| --- | --- | --- | --- | --- | --- | --- |
|  |  | 1.5 T | 3.0 T | 1.5 T | 3.0 T |  |
| **Viral myocarditis** |  |  |  |  |  |  |
| Thavendiranathan(9) | T2prep SSFP | 54.5±2.2 (n=30) |  | Involved 65.2±3.2  Remote 53.5±2.1 (n=20) |  | 4.0  0.33 |
| Radunski(15) | T2prep GraSE | 56 (54-60) (n=20) |  | 59(55-65) (n=20) |  | 0.6 |
| Baessler(16) | T2prep GraSE | 58.7 ± 4.2 (n=30) |  | 62.1 ± 7.2 (n=31) |  | 0.6 |
| Bohnen(3) | T2prep GraSE | 55(54-57) (n=11) |  | EMB+: 65 (61-70) (n=16)  EMB^_^:59 (55-64) (n=15) |  | 3.6  1.9 |
| Von Knobelsdorff-Brenkenhoff(17) | T2 SSFP | 50.2 (49.2–52.0)  (n=18) |  | 55.1(53.3–57.2)  (n=18) |  | 1.1 |
| **Systemic lupus Erythematosus** | |  |  |  |  |  |
| Hinojar (active)(18) | T2prep GraSE |  | 45 ± 4 (n=45) |  | 65 ± 8 (n=65) | 3.2 |
| Zhang (subclinical) (19) | T2 SSFP | 52.8±4.4 (n=12) |  | 58.2±5.6 (n=24) |  | 1.7 |
| **TakoTsubo Cardiomyopathy** | |  |  |  |  |  |
| Thavendiranathan(9) | T2 SSFP | 54.5±2.2 (n=30) |  | (n=10)  Involved 65.6±4.0  Remote 53.6±2.7 |  | 3.5  0.2 |
| **Cardiac Sarcoidosis** |  |  |  |  |  |  |
| Greulich 2016 (20) | T2 SSFP | 49 (n=26) |  | 52 (n=61) |  |  |
| Puntmann 2017(21) | T2 GraSE |  | 45±4 (n=21) |  | 54±6 (n=53) | 1.8 |
| **Acute myocardial infarction** | |  |  |  |  |  |
| Wasmuth 2013(12) | T2 SSFP | 55±5 (n=28) |  | (n=28)  Involved 73±9  Remote 51±3 |  | 2.4  0.97 |
| Verhaert(11) | T2 SSFP | 55.5 ± 2.3 (n=21) |  | (n=27)  Involved 69 ±6  Remote 56±3.4 |  | 4.65  0.17 |

References

1. Fernández-Jiménez R, Sánchez-González J, Agüero J, Del Trigo M, Galán-Arriola C, Fuster V, et al. Fast T2 gradient-spin-echo (T2-GraSE) mapping for myocardial edema quantification: first in vivo validation in a porcine model of ischemia/reperfusion. Journal of Cardiovascular Magnetic Resonance. 2015 Nov 4;17(1):652.

2. Carpenter J-P, He T, Kirk P, Roughton M, Anderson LJ, de Noronha SV, et al. Calibration of myocardial T2 and T1 against iron concentration. Journal of Cardiovascular Magnetic Resonance. 2014 Aug 12;16(1):348.

3. Bohnen S, Radunski UK, Lund GK, Kandolf R, Stehning C, Schnackenburg B, et al. Performance of T1 and T2 Mapping Cardiovascular Magnetic Resonance to Detect Active Myocarditis in Patients With Recent-Onset Heart Failure. Circulation: Cardiovascular Imaging. 2015 May 19;8(6):e003073–3.

4. Lurz P, Luecke C, Eitel I, Föhrenbach F, Frank C, Grothoff M, et al. Comprehensive Cardiac Magnetic Resonance Imaging in Patients With Suspected Myocarditis. Journal of the American College of Cardiology. 2016 Apr;67(15):1800–11.

5. Bulluck H, White SK, Rosmini S, Bhuva A, Treibel TA, Fontana M, et al. T1 mapping and T2 mapping at 3T for quantifying the area-at-risk in reperfused STEMI patients. Journal of Cardiovascular Magnetic Resonance. 2015 Aug 12;17(1):2605.

6. Park CH, Choi E-Y, Yoon YW, Kwon HM, Hong BK, Lee BK, et al. Quantitative T2 mapping after reperfusion therapy in patients with acute myocardial infarction: A comparison with late gadolinium enhancement and cine MR imaging. Magnetic Resonance Imaging. 2015 Dec;33(10):1246–52.

7. Langhans B, Nadjiri J, Jähnichen C, Kastrati A, Martinoff S, Hadamitzky M. Reproducibility of area at risk assessment in acute myocardial infarction by T1- and T2-mapping sequences in cardiac magnetic resonance imaging in comparison to Tc99m-sestamibi SPECT. Int J Cardiovasc Imaging. 2014 Jul 2;30(7):1357–63.

8. Nassenstein K, Nensa F, Schlosser T, Bruder O, Umutlu L, Lauenstein T, et al. Cardiac MRI: T2-Mapping Versus T2-Weighted Dark-Blood TSE Imaging for Myocardial Edema Visualization in Acute Myocardial Infarction. Fortschr Röntgenstr. 2014 Jan 23;186(02):166–72.

9. Thavendiranathan P, Walls M, Giri S, Verhaert D, Rajagopalan S, Moore S, et al. Improved Detection of Myocardial Involvement in Acute Inflammatory Cardiomyopathies Using T2 Mapping. Circulation: Cardiovascular Imaging. 2012 Jan 17;5(1):102–10.

10. van Heeswijk RB, Piccini D, Feliciano H, Hullin R, Schwitter J, Stuber M. Self-navigated isotropic three-dimensional cardiac T2 mapping. Magn Reson Med. 2015 Apr;73(4):1549–54.

11. Verhaert D, Thavendiranathan P, Giri S, Mihai G, Rajagopalan S, Simonetti OP, et al. Direct T2 quantification of myocardial edema in acute ischemic injury. JACC: Cardiovascular Imaging. 2011 Mar;4(3):269–78.

12. Wassmuth R, Prothmann M, Utz W, Dieringer M, Knobelsdorff-Brenkenhoff von F, Greiser A, et al. Variability and homogeneity of cardiovascular magnetic resonance myocardial T2-mapping in volunteers compared to patients with edema. Journal of Cardiovascular Magnetic Resonance. 2013;15(1):27.

13. Knobelsdorff-Brenkenhoff von F, Prothmann M, Dieringer MA, Wassmuth R, Greiser A, Schwenke C, et al. Myocardial T1 and T2 mapping at 3 T: reference values, influencing factors and implications. Journal of Cardiovascular Magnetic Resonance. 2013;15(1):53.

14. Bönner F, Janzarik N, Jacoby C, Spieker M, Schnackenburg B, Range F, et al. Myocardial T2 mapping reveals age- and sex-related differences in volunteers. Journal of Cardiovascular Magnetic Resonance. 2015;17(1):9.

15. Radunski UK, Lund GK, Stehning C, Schnackenburg B, Bohnen S, Adam G, et al. CMR in Patients With Severe Myocarditis. JACC: Cardiovascular Imaging. 2014 Jul;7(7):667–75.

16. Baeßler B, Schaarschmidt F, Dick A, Stehning C, Schnackenburg B, Michels G, et al. Mapping tissue inhomogeneity in acute myocarditis: a novel analytical approach to quantitative myocardial edema imaging by T2-mapping. Journal of Cardiovascular Magnetic Resonance. 2015 Dec 23;17(1):165.

17. Knobelsdorff-Brenkenhoff von F, Schüler J, Dogangüzel S, Dieringer MA, Rudolph A, Greiser A, et al. Detection and Monitoring of Acute Myocarditis Applying Quantitative Cardiovascular Magnetic Resonance. Circulation: Cardiovascular Imaging. American Heart Association, Inc; 2017 Feb;10(2):e005242.

18. Hinojar R, Foote L, Sangle S, Marber M, Mayr M, Carr-White G, et al. Native T1 and T2 mapping by CMR in lupus myocarditis: Disease recognition and response to treatment. Int J Cardiol. 2016 Nov;222:717–26.

19. Zhang Y, Corona-Villalobos CP, Kiani AN, Eng J, Kamel IR, Zimmerman SL, et al. Myocardial T2 mapping by cardiovascular magnetic resonance reveals subclinical myocardial inflammation in patients with systemic lupus erythematosus. Int J Cardiovasc Imaging. 2014 Oct 29;31(2):389–97.

20. Greulich S, Kitterer D, Latus J, Aguor E, Steubing H, Kaesemann P, et al. Comprehensive Cardiovascular Magnetic Resonance Assessment in Patients With Sarcoidosis and Preserved Left Ventricular Ejection Fraction. Circulation: Cardiovascular Imaging. American Heart Association, Inc; 2016 Nov;9(11):e005022.

21. Puntmann VO, Isted A, Hinojar R, Foote L, Carr-White G, Nagel E. T1 and T2 Mapping in Recognition of Early Cardiac Involvement in Systemic Sarcoidosis. Radiology. 2017 Apr 27;:162732.
