# Supplementary material for: Society for Cardiovascular Magnetic Resonance (SCMR) expert consensus for CMR imaging endpoints in clinical research: part I - analytical validation and clinical qualification
Source: J Cardiovasc Magn Reson. 2018 Sep 20;20:67. doi: 10.1186/s12968-018-0484-5 (PMC6147157; doi:10.1186/s12968-018-0484-5)
Supplement: Supplementary file 6 — T2* mapping tables. Table 3c-iii.1: Correlation of T2* mapping indices with histological substrates. Table 3c-iii.2: Correlation of myocardial native T2* mapping with other imaging biomarkers. Table 3c-iii.3: Intra, interobserver and interstudy variability reported for native T2* using various sequences and field strengths. Studies reported if included interstudy reproducibility. Table 3c-iii.4: Normal values for myocardial and liver native T2* reported for different sequences and magnetic fields. Table 3c-iii.5: Proof of concept studies with T2* indices differentiating between health and disease. Table 3c-iii.6: Outcome studies and treatment comparisons’ studies using T2* indices. (DOCX 66 kb) [file 12968_2018_484_MOESM6_ESM.docx]

# T2* mapping tables

**Table 3c-iii.1: Correlation of T2* mapping indices with histological substrates.** Agreement expressed as Pearson r-coefficient, linear R^2^ regression index or area under the curve (AUC). mb – multiple breath-hold, GRE – gradient echo, BB – black blood, I/R – ischaemia – reperfusion model

|  | **N** | **Population** | **Sequence** | **Histological correlation** | **Agreement** | |
| --- | --- | --- | --- | --- | --- | --- |
| **Cardiac iron loading** | | |  |  |  | |
| Carpenter[1] | 12 | ExVivo Hearts | T2*GRE(BB) | Iron content | Native R2* (=1/T2*) | R^2^=0.91 |
| Anderson[2] | 30 | Liver biopsy | mbT2*GRE(BB) | Iron Content | Log_e_ liver native T2* | R=0.93 |
| **Acute MI – intramyocardial haemorrhage** | | |  |  |  |  |
| Ghurge[3] | 8 | Pigs (I/R injury) | T2*GRE(BB) | Histology | Native T2* (ms) | Qualitative |
| Kali[4] | 20 | Canines (acute I/R and chronic MI, day 56) | T2*GRE(BB) | Histology | Native T2* (ms) | Acute vs. ex vivo R^2^=0.9; p<0.001  Chronic vs. ex vivo, R^2^=0.9; p<0.001  Chronic vs. histology, R^2^=0.7, p<0.001 |
| Kali[5] | 20 | Canines (I/R injury) | T2*GRE(BB) | Histology | Native T2* (ms) | R^2^=0.7; p<0.001 |
| House[6] | 2 | Human (transfusion iron overload) | T2*GRE(BB) (R2* map) | Synchrotron | Tissue iron map content | Correlation plots |

**Table 3c-iii.2. Correlation of myocardial native T2* mapping with other imaging biomarkers.** §T2*<20msec; δT2*<10 msec.

mb – multiple breath-hold, GRE – gradient echo, BB – black blood, SWI – susceptibility weighted imaging; HPF – high pass filter. CNR – contrast-to-noise ratio

| **Myocardial native T2*** | **N** | **T2 mapping sequence** | **Population** | **Imaging biomarker** | **Outcome/Agreement** |
| --- | --- | --- | --- | --- | --- |
| **Cardiac iron loading** | | |  |  |  |
| Anderson[2] | 109 | mbT2*GRE(BB) | Thalassemia major | Liver T2* | R=0.15, p=0.11 |
|  |  |  |  | §LV EF (%) | R=0.61, p<0.001 |
|  |  |  |  | §LV ESVi(mL/m2) | R=0.50, p<0.001 |
|  |  |  |  | §LVmassi (g/m2) | R=0.40, p<0.001 |
| Westwood[7] | 67 | T2*GRE(BB) | Thalassemia major | §E/A ratio | R=-0.62, p<0.01 |
|  |  |  |  | §A-wave | R=0.49, p<0.001 |
| Tanner[8] | 65 | T2* GRE(BB) | Thalassemia major | δLV-EF (%) | R=0.67, p<0.001 |
| Marsella[9] | 776 | T2* GRE(BB) | Thalassemia major | LV-EF(%) | R2*: R= -0.327, p<0.0001 |
| Carpenter [1] | 31 | T2*GRE(BB) | Hemochromatosis | §LV-EF(%) | R=0.57, 0.049 |
| **Acute MI – intramyocardial haemorrhage** | | |  |  |  |
| O’Regan[10] | 15 | T2*GRE(BB) | STEMI | T2WI-STIR  LGE | Qualitative analysis |
| O’Regan[11] | 50 | T2*GRE(BB) | STEMI | T2WI-STIR | Qualitative analysis |
| Zia[12] |  | T2*GRE(BB) | STEMI | T2WI-STIR | Qualitative analysis |
| Kandler[13] | 151 | T2*GRE(BB) | STEMI | T2WI-STIR | T2* mapping had superior diagnostic accuracy vs. T2W-STIR (16% false negative, 24% false positive). |
| Kidambi[14] | 49 | T2*GRE(BB)  SWI | STEMI | T2W-STIR | SW MRI had sensitivity of 93% and specificity of 86% |
| Carrick[15] | 245 | T2*GRE(BB) | STEMI | T2 map | T2* mapping had superior diagnostic accuracy vs. T2 map |
| Durighel[16] | 30 | T2*GRE(BB)  SWI | STEMI | T2WI-STIR  HPF | CNR with SWI was higher than other methods |
| Bulluck[17] | 48 | T2*GRE(BB) | STEMI | T1 map  T2 map | T2* hypointense core is taken as the reference dataset |

**Table 3c-iii.3.** **Intra, interobserver and interstudy variability reported for native T2* using various sequences and field strengths. Studies reported if included interstudy reproducibility.** CoV%(coefficient of variation); mb – multiple breath-hold, GRE – gradient echo; BB – black blood.

| **T2* mapping (msec)** | Anderson[2] | Westwood[18,19] | Tanner[20] |
| --- | --- | --- | --- |
| **Magnetic field** | 1.5 | 1.5 | 1.5 |
| **N** | 10 | 10 | 39 |
| **Population** | Thalassemia major | Thalassemia major | Thalassemia major |
| **Centres** | 1 | 1 | 6 |
| **Sequence** | mbT2* GRE (BB) | T2* GRE(BB) | T2* GRE(BB) |
| **No of echo images** | 9 | 9 | 9 |
| **Interobserver V** | Heart 6.4%  Liver 4.5% |  |  |
| **Intraobserver V** |  |  |  |
| **Interstudy V** | Heart 5.0%  Liver 3.3% | Heart 5.3% T2*<20: 2.3%  T2*>20: 9.3% | Heart 5.8%  Liver 4.4% |
| **Inter-centre V** |  | Heart 9.4%  Liver 7.9% | Heart 5.0%  Liver 7.1% |

**Table 3c-iii.4: Normal values for myocardial and liver native T2* reported for different sequences and magnetic fields.**

Mean native T2 values±SD or 95%CI in single mid-ventricular slice, expressed in ms. Septal ROIs, § global (average measurement of 3 short axis slices). mb – multiple breath-hold, GRE – gradient echo; BB – black blood; WB – white blood.

|  | **N** | **Age (years, range)** | **Sequence** | **Native T2*(msec)** | | | | | |
| --- | --- | --- | --- | --- | --- | --- | --- | --- | --- |
|  |  |  |  | **1.5 T** | | | **3.0 T** | | |
|  |  |  |  | **Myocardium** | **Liver** | **Myocardium** | | **Liver** |  |
| Anderson[2] | 15 | 32(26-39) | mbT2*GRE(BB) | 52±16 | 33±7 |  | |  |  |
| Westwood[18] | 10 | 49±26 | mbT2*GRE(BB) | 30.1±7.1 | 26.6±4.7 |  | |  |  |
|  |  |  | T2* GRE(BB) | 33.3±7.8 | 26.7±4.2 |  | |  |  |
| Rammazotti[21] | 5 | 35±10 | T2* GRE(BB) | 39±7.3  §36±5 | 23±3.6 |  | |  |  |
| Alam[22] | 20 | 35(26-33) | T2* GRE(WB) | 32.3(28.9-36.7) | 25.8(23.1-28.0) | 20.5(18.3-24.3) | | 17.3(14.8-21.4) |  |
| Carrick[15] | 50 | 54±13 years  26 (52%) male | T2*GRE(BB) | 31.0 ± 2.1 |  |  | |  |  |

**Table 3c-iii.5. Proof of concept studies with T2* indices differentiating between health and disease.**

The table reports mean values±SD for each disease entity, sequence type, T2* index, and field strength; includes effect size as a measure of dispersion observed in healthy subjects. Native T2* values are expressed in msec. § global (average measurement of 3 short axis slices). deferoxamine (DFO), deferiprone (DFP), combined regime (DPO+DFP). HR(95%CI): hazard ratio, 95% confidence interval. mb – multiple breath-hold, GRE – gradient echo, BB – black blood.

| **Disease model** | **Sequence** | | | **Health**  **Average T2* in ms (n)** | **Disease Average T2* in ms (n)** | |
| --- | --- | --- | --- | --- | --- | --- |
| ***Thalassemia major*** | | | | 1.5 T | 1.5 T | |
| Anderson[23] | mbT2*GRE(BB) | | | / | 11.4 (treatment with DFO; n=30)  34.0 (treatment with DFP; n=15) | |
| Anderson[2] | mbT2*GRE(BB) | | | / | Cardiac T2* predictive of the need for cardiac medication with (HR (95%CI): 0.81 (0.71-0.93), p=0.003; n=109) | |
| Tanner[8] | T2* GRE(BB) | | | / | 11.4 (treatment with DFO; n=30)  32.0 (treatment with DFO+DFP; n=15) | |
| Rammazotti[21] | T2* GRE(BB) | | | 39±7.3 (n=5) | 24 (n=5) | |
| Casale[24] | T2* GRE(BB) | | | / | 34 (n=107)  §38.8 (n=107) | |
| Alam [25] | T2*GRE(BB) | | | 30.8(29.0-34.4) (n=20) | 28.1 (n=53) | |
| ***Hemochromatosis*** | | | |  |  | |
| Carpenter[26] | T2*GRE(BB) | | | / | 34.8 (genetically confirmed hemochromatosis, n=31) | |
| ***Acute myocardial infarction*** | | | |  |  | |
| O’Regan[11] | | T2*GRE(BB) |  | | | Affected - haemorrhage 15.4 ± 5.7 ms  Affected – no haemorrhage 47.2±13.8 ms |
| Zia [12] | | T2*GRE(BB) |  | | | Day 2  Affected – 32.4 ms  Remote – 37.4 ms  3 weeks  Affected – 37.7 ms  Remote – 38.4 ms  3 weeks  Affected – 37.3 ms  Remote – 38.2 ms |
| Kali[5] | | T2*GRE(BB) |  | | | Affected - haemorrhage 15.9± 4.5 ms  Affected – no haemorrhage 37.8±2.5 ms  Remote - 35.2 ± 2.1 ms |
| Durighel[16] | | T2*GRE(BB) |  | | | Affected - haemorrhage 33.5 ms [24.9 - 43]  Affected – no haemorrhage 49.9 ms[44.6 - 67.6]  Remote 44.9 ms [38.8 – 51.4] |
| Carrick[27] | | T2*GRE(BB) | 31.0±2.1 | | | Table 3 & time course  See below |
| Bulluck[17] | | T2*GRE(BB) |  | | | Affected - haemorrhage 13.3 ms [24.9-43]  Remote 33 ± 4 ms |

**Table 3c-iii.6. Outcome studies and treatment comparisons’ studies using T2* indices.**

deferoxamine (DFO), deferiprone (DFP), combined regime (DPO+DFP), GRE – gradient echo, BB – black blood, FMD – flow-mediated dilatation, RR – relative risk, mb – multiple breath-hold.

|  | **Type** | **Population** | **N** | **Follow-up (months)** | **Sequence** | **Field Strength** | **Endpoint** | **Statistics** |
| --- | --- | --- | --- | --- | --- | --- | --- | --- |
| Tanner [20] | RCT multicentre | Thalassaemia major   - DFO and placebo - DFO+DFP | 65 | 12 | T2* GRE(BB) | 1.5T | Δcardiac T2* | Absolute percent difference:  ~10% (95%CI 2-19%), p=0.02 |
|  |  |  |  |  |  |  | LV-EF | Absolute percent difference  1.17% (95% CI 0.0-2.35%), p=0.05 |
|  |  |  |  |  |  |  | Brachial FMD | Absolute percent difference: 5.9%(95%CI 0.99-10.8), p=0.02 |
| Tanner [28] | Observational two centre open-label | Thalassaemia major:   - DFP+DFO | 15 | 12 | T2* GRE(BB) | 1.5T | Δcardiac T2* | baseline 5.7±0.98ms  12 months: 7.9±2.47ms  (p = 0.010) |
|  |  |  |  |  |  |  | LV-EF | baseline 51.2±10.9%  12 months: 65.7±6.7%  (p = 0.010) |
| Kirk [29] | Observational multicentre outcome | Thalassaemia major | 652 | 12 | T2* GRE(BB) | 1.5T | Heart failure | T2*=10msec predictive of HF :   - sensitivity 97.5% (95% CI, 91.3-99.7) - specificity of 85.3% (95% CI, 83.3-87.2).   RR T2*<10 ms:   - 8 to 10 ms: 2.97 - 6 to 8 ms: 3.48 - <6 ms: 4.51 (p< 0.001) |
|  |  |  |  |  |  |  | Arrhythmia | - T2*=20msec predictive of arrhythmia - sensitivity 82.7% (95% CI 73.7-89.6) - specificity of 53.5% (95% CI 50.8-56.2).   RR T2*<20 ms:   - 15 to 20 ms : 2.21 - 10 to 15 ms 3.23 - 8 to 10 ms: 6.82 - 6 to 8 ms: 7.5 - <6 ms: 8.78 (p< 0.001) |
| Pepe [30] | Observational multicentre study | Thalassaemia major: stable treatment with:   - DFP - DFO - DFP+ DFO | 164 | 18 | T2* GRE(BB) | 1.5T | Δ mean cardiac T2* between groups | The improvement in the global heart T2* was significantly higher in the DFP+DFO than the DFO group, without a difference in biventricular function |
| Pennell [31] | RCT multicentre | Thalassaemia major   - DFO - DFP | 61 | 12 | mbT2* GRE(BB) | 1.5T | Δcardiac T2* | DFO: 13%  DFP: 27%  (p=0.023) |
|  |  |  |  |  |  |  | ΔLV-EF | DFO: 0.3%  DFP: 3.1%  (p=0.03) |
| Pennell [32] | RCT multicentre | Thalassaemia major   - DFO - Deferasirox | 197 | 12 | mbT2* GRE(BB) | 1.5T | Δcardiac T2* | DFO: 7%  Deferasirox: 12%  Non-inferiority criteria met |
|  |  |  |  |  |  |  | ΔLV-EF | DFO: 0%  Deferasirox: -0.6%  P=0.54 |

**References**

1. Carpenter J-P, He T, Kirk P, Roughton M, Anderson LJ, de Noronha SV, et al. On T2* magnetic resonance and cardiac iron. Circulation. American Heart Association, Inc; 2011;123:1519–28.

2. Anderson LJ, Holden S, Davis B, Prescott E, Charrier CC, Bunce NH, et al. Cardiovascular T2-star (T2*) magnetic resonance for the early diagnosis of myocardial iron overload. European Heart Journal. 2001;22:2171–9.

3. Ghugre NR, Ramanan V, Pop M, Yang Y, Barry J, Qiang B, et al. Quantitative tracking of edema, hemorrhage, and microvascular obstruction in subacute myocardial infarction in a porcine model by MRI. Magn. Reson. Med. 2011;66:1129–41.

4. Kali A, Kumar A, Cokic I, Tang RLQ, Tsaftaris SA, Friedrich MG, et al. Chronic manifestation of postreperfusion intramyocardial hemorrhage as regional iron deposition: a cardiovascular magnetic resonance study with ex vivo validation. Circulation: Cardiovascular Imaging. American Heart Association, Inc; 2013;6:218–28.

5. Kali A, Tang RLQ, Kumar A, Min JK, Dharmakumar R. Detection of Acute Reperfusion Myocardial Hemorrhage with Cardiac MR Imaging: T2 versus T2*. Radiology. Radiological Society of North America; 2013;269:387–95.

6. House MJ, Fleming AJ, de Jonge MD, Paterson D, Howard DL, Carpenter J-P, et al. Mapping iron in human heart tissue with synchrotron x-ray fluorescence microscopy and cardiovascular magnetic resonance. Journal of Cardiovascular Magnetic Resonance. BioMed Central; 2014;16:80.

7. Westwood MA, Wonke B, Maceira AM, Prescott E, Walker JM, Porter JB, et al. Left ventricular diastolic function compared with T2* cardiovascular magnetic resonance for early detection of myocardial iron overload in thalassemia major. J. Magn. Reson. Imaging. Wiley Subscription Services, Inc., A Wiley Company; 2005;22:229–33.

8. Tanner MA, Galanello R, Dessi C, Smith GC, Westwood MA, Agus A, et al. A randomized, placebo-controlled, double-blind trial of the effect of combined therapy with deferoxamine and deferiprone on myocardial iron in thalassemia major using cardiovascular magnetic resonance. Circulation. American Heart Association, Inc; 2007;115:1876–84.

9. Marsella M, Borgna-Pignatti C, Meloni A, Caldarelli V, Dell'Amico MC, Spasiano A, et al. Cardiac iron and cardiac disease in males and females with transfusion-dependent thalassemia major: a T2* magnetic resonance imaging study. Haematologica. Haematologica; 2011;96:515–20.

10. O'Regan DP, Ahmed R, Karunanithy N, Neuwirth C, Tan Y, Durighel G, et al. Reperfusion hemorrhage following acute myocardial infarction: assessment with T2* mapping and effect on measuring the area at risk. Radiology. 2009;250:916–22.

11. O'Regan DP, Ariff B, Neuwirth C, Tan Y, Durighel G, Cook SA. Assessment of severe reperfusion injury with T2* cardiac MRI in patients with acute myocardial infarction. Heart. BMJ Publishing Group Ltd; 2010;96:1885–91.

12. Zia MI, Ghugre NR, Connelly KA, Strauss BH, Sparkes JD, Dick AJ, et al. Characterizing Myocardial Edema and Hemorrhage Using Quantitative T2 and T2* Mapping at Multiple Time Intervals Post ST-Segment Elevation Myocardial Infarction. Circulation: Cardiovascular Imaging. 2012;5:566–72.

13. Kandler D, Lücke C, Grothoff M, Andres C, Lehmkuhl L, Nitzsche S, et al. The relation between hypointense core, microvascular obstruction and intramyocardial haemorrhage in acute reperfused myocardial infarction assessed by cardiac magnetic resonance imaging. Eur Radiol. Springer Berlin Heidelberg; 2014;24:3277–88.

14. Kidambi A, Biglands JD, Higgins DM, Ripley DP, Zaman A, Broadbent DA, et al. Susceptibility-weighted cardiovascular magnetic resonance in comparison to T2 and T2 star imaging for detection of intramyocardial hemorrhage following acute myocardial infarction at 3 Tesla. Journal of Cardiovascular Magnetic Resonance. BioMed Central; 2014;16:86.

15. Carrick D, Haig C, Ahmed N, McEntegart M, Petrie MC, Eteiba H, et al. Myocardial Hemorrhage After Acute Reperfused ST-Segment-Elevation Myocardial Infarction: Relation to Microvascular Obstruction and Prognostic Significance. Circulation: Cardiovascular Imaging. American Heart Association, Inc; 2016;9:e004148.

16. Durighel G, Tokarczuk PF, Karsa A, Gordon F, Cook SA, O'Regan DP. Acute myocardial infarction: susceptibility-weighted cardiac MRI for the detection of reperfusion haemorrhage at 1.5 T. Clin Radiol. Elsevier; 2016;71:e150–6.

17. Bulluck H, White SK, Rosmini S, Bhuva A, Treibel TA, Fontana M, et al. T1 mapping and T2 mapping at 3T for quantifying the area-at-risk in reperfused STEMI patients. Journal of Cardiovascular Magnetic Resonance. 2015;17:2605.

18. Westwood M, Anderson LJ, Firmin DN, Gatehouse PD, Charrier CC, Wonke B, et al. A single breath-hold multiecho T2* cardiovascular magnetic resonance technique for diagnosis of myocardial iron overload. J. Magn. Reson. Imaging. Wiley Subscription Services, Inc., A Wiley Company; 2003;18:33–9.

19. Westwood MA, Anderson LJ, Firmin DN, Gatehouse PD, Lorenz CH, Wonke B, et al. Interscanner reproducibility of cardiovascular magnetic resonance T2* measurements of tissue iron in thalassemia. J. Magn. Reson. Imaging. Wiley Subscription Services, Inc., A Wiley Company; 2003;18:616–20.

20. Tanner MA, He T, Westwood MA, Firmin DN, Pennell DJ, Thalassemia International Federation Heart T2* Investigators. Multi-center validation of the transferability of the magnetic resonance T2* technique for the quantification of tissue iron. Haematologica. 2006;91:1388–91.

21. Ramazzotti A, Pepe A, Positano V, Rossi G, De Marchi D, Brizi MG, et al. Multicenter validation of the magnetic resonance T2* technique for segmental and global quantification of myocardial iron. J. Magn. Reson. Imaging. Wiley Subscription Services, Inc., A Wiley Company; 2009;30:62–8.

22. Alam MH, Auger D, McGill L-A, Smith GC, He T, Izgi C, et al. Comparison of 3 T and 1.5 T for T2* magnetic resonance of tissue iron. Journal of Cardiovascular Magnetic Resonance. BioMed Central; 2016;18:40.

23. Anderson LJ, Wonke B, Prescott E, Holden S, Walker JM, Pennell DJ. Comparison of effects of oral deferiprone and subcutaneous desferrioxamine on myocardial iron concentrations and ventricular function in beta-thalassaemia. Lancet. 2002;360:516–20.

24. Casale M, Meloni A, Filosa A, Cuccia L, Caruso V, Palazzi G, et al. Multiparametric Cardiac Magnetic Resonance Survey in Children With Thalassemia Major: A Multicenter Study. Circulation: Cardiovascular Imaging. American Heart Association, Inc; 2015;8:e003230.

25. Alam MH, Auger D, Smith GC, He T, Vassiliou V, Baksi AJ, et al. T1 at 1.5T and 3T compared with conventional T2* at 1.5T for cardiac siderosis. Journal of Cardiovascular Magnetic Resonance. BioMed Central; 2015;17:102.

26. Carpenter J-P, Grasso AE, Porter JB, Shah F, Dooley J, Pennell DJ. On myocardial siderosis and left ventricular dysfunction in hemochromatosis. Journal of Cardiovascular Magnetic Resonance. BioMed Central; 2013;15(1):24.

27. Carrick D, Haig C, Ahmed N, Rauhalammi S, Clerfond G, Carberry J, et al. Temporal Evolution of Myocardial Hemorrhage and Edema in Patients After Acute ST-Segment Elevation Myocardial Infarction: Pathophysiological Insights and Clinical Implications. J Am Heart Assoc. American Heart Association, Inc; 2016;5:e002834.

28. Tanner MA, Galanello R, Dessi C, Smith GC, Westwood MA, Agus A, et al. Combined chelation therapy in thalassemia major for the treatment of severe myocardial siderosis with left ventricular dysfunction. Journal of Cardiovascular Magnetic Resonance. BioMed Central; 2008;10:12.

29. Kirk P, Roughton M, Porter JB, Walker JM, Tanner MA, Patel J, et al. Cardiac T2* magnetic resonance for prediction of cardiac complications in thalassemia major. Circulation. American Heart Association, Inc; 2009;120:1961–8.

30. Pepe A, Meloni A, Rossi G, Cuccia L, D'Ascola GD, Santodirocco M, et al. Cardiac and hepatic iron and ejection fraction in thalassemia major: multicentre prospective comparison of combined deferiprone and deferoxamine therapy against deferiprone or deferoxamine monotherapy. Journal of Cardiovascular Magnetic Resonance. BioMed Central; 2013;15:1.

31. Pennell DJ1, Berdoukas V, Karagiorga M, Ladis V, Piga A, Aessopos A, et al. Randomized controlled trial of deferiprone or deferoxamine in beta-thalassemia major patients with asymptomatic myocardial siderosis. Blood. 2006;107:3738-44.

32. Pennell DJ1, Porter JB, Piga A, Lai Y, El-Beshlawy A, Belhoul KM, et al. A 1-year randomized controlled trial of deferasirox vs deferoxamine for myocardial iron removal in β-thalassemia major (CORDELIA). Blood. 2014;123:1447-54.
