# Supplementary material for: Society for Cardiovascular Magnetic Resonance (SCMR) expert consensus for CMR imaging endpoints in clinical research: part I - analytical validation and clinical qualification
Source: J Cardiovasc Magn Reson. 2018 Sep 20;20:67. doi: 10.1186/s12968-018-0484-5 (PMC6147157; doi:10.1186/s12968-018-0484-5)
Supplement: Supplementary file 7 — Myocardial perfusion imaging with CMR. Table 3d.1: Studies comparing CMR perfusion against microspheres and alternative diagnostic approaches. Table 3d.2: Interstudy reproducibility and normal values of myocardial perfusion reserve (index). Table 3d.3: Prospective outcome studies using stress CMR. (DOCX 107 kb) [file 12968_2018_484_MOESM7_ESM.docx]

## Myocardial perfusion imaging with CMR

**Table 3d.1: Studies comparing CMR perfusion against microspheres and alternative diagnostic approaches**. MBF – myocardial blood flow (ml/min/g) calculated from Fermi deconvolution. SPECT - single-photon emission computed tomography. PET - positron emission tomography. CAD – coronary artery disease. MRPI – myocardial perfusion reserve index calculated from relative upslopes. FFR – fractional flow reserve. LAD – left anterior descending artery. LCX – left circumflex artery. RCA – right coronary artery. PCI – percutaneous coronary intervention. CABG – coronary artery bypass graft. AUC – area under the curve. CT: computed tomography. TPGR - transmural perfusion gradient reserve. §-ischemic burden: the summed stress score and summed rest score taken from the LGE images were obtained by adding segmental scores, and the summed difference score (ie, summed stress score–summed rest score) was expressed as a percentage of 68, the theoretical maximum score. Studies included if n>25 patients. N/A for healthy volunteer studies.

| **Correlation with microspheres** | | | | | | | | | |
| --- | --- | --- | --- | --- | --- | --- | --- | --- | --- |
| **Author** | N | Animal model | Stress | Field strength | Index |  |  | Correlations |  |
| **Klocke [1]** | 12 | Dogs | Adenosine | 1.5 | MBF (ml/min/g) | | | R^2^=0.87 | <0.001 |
| **Lee [2]** | 28 | Dogs | Adenosine | 1.5 | MBF (ml/min/g) | | | R^2^=0.77 | <0.001 |
| **Christian [3]** | 6 | Dogs | Adenosine | 1.5 | MBF (ml/min/g) - single bolus | | | R=0.91 | <0.001 |
|  |  |  |  |  | MBF (ml/min/g) - dual bolus | | | R=0.94 | <0.001 |
| **Christian [4]** | 9 | Pigs | Adenosine | 1.5 | MBF (ml/min/g) | | | R=0.95 | <0.001 |
|  | 8 | Pigs | Adenosine | 3.0 |  |  |  | R=0.98 | <0.001 |
| **Hsu [5]** | 7 | Dogs | Adenosine | 1.5 | MBF (ml/min/g) | | | R=0.87-0.90 | <0.001 |
| **Schuster [6]** | 9 | Explanted pig hearts | Adenosine | 1.5 | MBF (ml/min/g) - dual bolus | | | R=0.93 | <0.001 |
| **Schuster [7]** | 8 | Explanted pig hearts | Adenosine | 1.5 | MBF (ml/min/g) - dual bolus | | | R=0.94 | <0.001 |
|  |  |  |  | 3.0 |  |  |  | R=0.96 | <0.001 |

| **Agreement with PET and SPECT** | | | | | | | |
| --- | --- | --- | --- | --- | --- | --- | --- |
| **Author** | **N** | **Patient Population** | **Stress** | **Field strength** | **Index** | **Agreement** |  |
| **Schwitter [8]** | 66 | Healthy volunteers and CAD patients | Dipyridamole | 1.5 | MPRI Vs 13NH3 - PET | 0.76 | P<0.001 |
| **Ibrahim [9]** | 44 | Healthy volunteers and CAD patients | Adenosine | 1.5 | MPRI vs 13NH3 - PET | R=0.70 | P<0.001 |
| **Pärkkä [10]** | 18 | Healthy volunteers | Dipyridamole | 1.5 | MBF (ml/min/g) vs O2 PET | R=0.7 | <0.001 |
|  |  |  |  |  | MPRI vs O2 PET | R=0.46 | 0.04 |
| **Fritz-Hansen [11]** | 10 | Healthy volunteers | Dipyridamole | 1.5 | MBF (ml/min/g) vs 13NH3 - PET | R=0.79 | 0.02 |
| **Pack [12]** | 5 | Healthy volunteers | Adenosine | 3.0 | MBF (ml/min/g) vs 13NH3 - PET | R=0.85 | <0.001 |
| **Morton [13]** | 41 | Suspected CAD | Adenosine | 1.5 | MBF (ml/min/g) - dual bolus compared to 13NH3 – PET  MPRI | R=0.32  R=0.79 | <0.001  p<0.001 |
| **Jogiya R [14]** | 45 | Suspected CAD | Adenosine | 3.0 | Ischaemic burden§ | R=0.70 |  |
| **Qayyum[15]** | 14 | Suspected CAD | Adenosine | 1.5 | Tikhonov deconvolution  Comparison to 82-Rb PET  Absolute global myocardial perfusion reserve  Regional myocardial perfusion reserve | R=0.87  R=0.89-0.90 | P<0.001  P<0.001 |

| **Agreement with coronary angiography** | | | | | |  |  |  |  |  |  |
| --- | --- | --- | --- | --- | --- | --- | --- | --- | --- | --- | --- |
|  | | **N** | **Type** | **Stress** | **Reference (diameter/FFR)** | **Patient based results** | | | **Vessel based results** | | |
|  |  |  |  |  |  | Sensitivity | Specificity | Accuracy | Sensitivity | Specificity | Accuracy |
| **Al-Saadi [16]** | | 34 | Observational | Dipyridamole | ≥75% | 90 | 83 | 87 | 90 | 83 |  |
| **Al-Saadi**  **[17]** | | 27 | Observational | Dobutamine | ≥75% | 81 | 73 | 77 |  |  |  |
| **Nagel [18]** | | 84 | Observational | Adenosine | ≥75% | 88 | 90 | 89 |  |  |  |
| **Bunce [19]** | | 35 | Observational | Adenosine | ≥50% |  |  |  | 74 | 71 | 72 |
| **Cheng [20]** | | 61 | Observational | Adenosine | ≥50 | 1.5T: 90  3T: 98 | 1.5T: 67  3T: 76 | 1.5T: 82  3T: 90 |  |  |  |
| **Cury [21]** | | 47 | Observational | Adenosine | ≥50 |  |  | 88 | 87 | 89 | 88 |
| **Wolff [22]** | | 99 | Observational dose ranging multicentre | Adenosine | ≥70% | 93 (93-93) | 75 (68-82) | 85 (82-88) |  |  |  |
| **Giang [23]** | | 94 | Observational dose ranging, multicentre | Adenosine | ≥50 | 93 (77-99) | 75 (48-92) |  |  |  |  |
| **Gebker [24]** | | 101 | Observational | Adenosine |  | 90 | 71 | 84 |  |  |  |
| **Merkle [25]** | | 228 | Observational | Adenosine | >70%  >50% | 93  96 | 86  72 | 91  88 | LAD: 92  LCX: 82  RCA: 65 |  |  |
| **Kitagawa [26]** | | 50 | Observational, multicentre | Adenosine | >50% | 89 | 79 | 86 | 81 | 83 | 82 |
| **Klein,**  **[27]** | | 49 | Observational | Adenosine | ≥50% | 87 | 89 | 88 | LAD: 87  LCX: 69  RCA: 75 | 94  89  85 |  |
| **Klein [28]** | | 78 | Observational, after CABG | Adenosine | >50 | 77 | 90 | 82 | 63 | 91 | 81 |
| **Klem [29]** | | 92 | Observational | Adenosine | ≥70 | 89 | 87 | 88 |  |  |  |
| **Klem [30]** | | 136 | Observational, women | Adenosine | ≥70 | 84 | 88 | 87 |  |  |  |
| **Krittayaphong [31]** | | 66 | Observational | Adenosine |  | Visual: 87  MPRI: 90 | Visual: 75  MPRI: 79 |  |  |  |  |
| **Klumpp [32]** | | 57 | Observational | Adenosine | >70 | 95-98 | 80-87 | 91-95 | 95 | 89 |  |
| **Paetsch [33]** | | 79 | Observational | Adenosine | >50 | 91 | 62 | 81 |  |  |  |
| **Plein [34]** | | 92 | Observational | Adenosine |  | 88 | 82 | 79 |  |  |  |
| **Takase [35]** | | 102 | Observational | dipyridamole | >50 | 93 | 85 |  |  |  |  |
| **Thomas [36]** | | 60 | Observational | Adenosine |  | 93 | 84 |  |  |  |  |
| **Bernhardt [37]** | | 477 | Observational | Adenosine | ≥70 | Suspected CAD: 94  Previous PCI: 91  Previous CABG: 79 | Suspected CAD: 94  Previous PCI: 90  Previous CABG: 77 |  |  |  |  |
| **Manka [38]** | | 146 | Observational, 3D imaging | Adenosine | ≥50 | 92 | 74 | 83 |  |  |  |
| **Meyer [39]** | | 60 | Observational, | Adenosine | ≥70% | 89 | 79 | 85 | 76 | 86 |  |
| **Greenwood [40]** | | 752 | Observational | Adenosine | ≥70% | 86 (82-90) | 83 (79-87) | AUC 0.89 (0.86-91) |  |  |  |
|  |  |  |  |  |  | Superior sensitivity and similar specificity compared to SPECT | | | | | |
| **Greenwood [41]** | | 235 | Observational (subanalysis of women) | Adenosine | ≥70% | 89 | 83 | AUC 0.90 |  |  |  |
|  |  |  |  |  |  | Superior sensitivity and similar specificity compared to SPECT in women | | | | | |
| **Schwitter[42]** | | 515 | Observational, multicentre | Adenosine | ≥50% | 75 (69-80) | 59 (52-65) |  |  |  |  |
|  |  |  |  |  |  | Superior sensitivity and inferior specificity compared to SPECT | | | | | |
| **Jaarsma[43]** | | 2937 | Meta-analysis | Adenosine Dipyridamole Dobutamine | ≥50% | 89 (88-91) | 76 (73-78) | 86 | 84 (81-86) | 83 (81-86) | 84 |
|  |  |  |  |  |  | CMR had superior specificity than SPECT with similar diagnostic accuracy as PET | | | | | |
| **Doyle[44]** | | 184 | Observational | Dipyridamole | ≥70% | 58 | 78 | 76 |  |  |  |
| **Gebker[45]** | | 414 | Observational | Dobutamine | ≥50%/70% | 87/91 | 76/70 | 85/84 |  |  |  |
| **Merkle[25]** | | 256 | Observational | Adenosine | ≥50% | 91 | 82 | 89 |  |  |  |
| **Pilz [46]** | | 171 | Observational | Adenosine | ≥70% | 96 | 83 | 92 |  |  |  |
| **Jogiya [14]** | | 45 | Observational | Adenosine | ≥50% | 94(71-100) | 81(54-95) | 88 |  |  |  |
|  |  |  |  |  |  | CMR had superior specificity than SPECT with similar diagnostic accuracy as PET | | | | | |
| **Agreement with FFR** | | | | | | | | | | | |
| **Takx[47]** | 798 | | Meta-analysis | Adenosine | FFR<0.75 | 89 (86-92) | 87 (83-90) | 88 | 87 (84-90) | 91 (89-92) | 89 |
|  |  |  |  |  |  | CMR, CT and PET had similar diagnostic accuracy, and were superior to SPECT and echo | | | | | |
| **Watkins[48]** | 101 | | Observational | Adenosine | FFR<0.75 | 95 (87-99) | 91 (72-99) | 94 | 91 (84-95) | 94 (89-97) | 93 |
| **Manka[49]** | 120 | | Observational | Adenosine | FFR<0.75 | 90 (80-96) | 82 (69-92) | 87 |  |  |  |
| **Manka [50]** | 155 | | Observational, multicentre | Adenosine | FFR <0.8 | 85 | 91 |  |  |  |  |
| **Bettencourt[51]** | 103 | | Observational | Adenosine | FFR<0.80 | 89 (76-96) | 88 (77-95) | 88 | 80 (69-88) | 93 (89-96) | 90 |
| **Ebersberger[52]** | 116 | | Observational | Adenosine | FFR≤0.80 | 85 (70-94) | 87 (77-94) | 86 | 89 (80-95) | 95 (91-98) | 93 |
| **Groothuis[53]** | 192 | | Observational | Adenosine | FFR≤0.75 | 85 (65-96) | 82 (71-91) | 83 |  |  |  |
| **Layland [54]** | 106 | | Observational | Adenosine | FFR≤0.80 | 86 | 95 |  | 87.2 | 91.9 |  |
| **Li [55]** | 650 | | Meta-Analysis | Adenosine | FFR ≤0.75 or 0.80 | 90 (86-93) | 87 (82-90) |  | 89 (83-92) | 86 (77-92) |  |
| **Costa [56]** | 30 | | Observational | Adenosine | FFR≤0.75 |  |  |  | Segmental: 93 | Segmental:  57 |  |
| **Lockie [5]** | 42 | | Observational | Adenosine | FFR≤0.75 |  |  |  | 82 | 94 |  |
| **Huber [57]** | 31 | | Observational | Adenosine | >75 or >50 and FFR <0.75 |  |  | AUC: 0.92 |  |  |  |
| **Jogiya [58]** | 53 | | Observational, 3D whole heart | Adenosine | FFR <0.75 | 91 (76-98) | 90 (67-99) | 91 | 79 (64-89) | 92 (85-96) | 88 |
| **Pan [59]** | 71 | | Observational, TPGR | Adenosine | FFR ≤ 0.75 | 91 | 90 |  |  |  |  |
| **Bernhardt [60]** | 34 | | Observational | Adenosine | FFR≤0.80 | 90 (70-99) | 100 (75-100) |  | 87 (72-96) | 98 (92-100) |  |
| **Kirschbaum, [61]** | 50 | | Observational | Adenosine | FFR <0.8 |  |  |  | 97 (84-100) | 60 (44-75) |  |
| **Rieber[62]** | 43 | | Observational | Adenosine | FFR≤0.75 |  |  |  | 88 (68-97) | 90 (82-95) |  |
| **Kühl [63]** | 28 | | Observational | Adenosine | FFR≤0.75 |  |  |  | 92 (62-100) | 91 (79-98) |  |

**Table 3d.2: Interstudy reproducibility and normal values of myocardial perfusion reserve (index)**. Studies included, if reporting interstudy reproducibility. Variability is presented as coefficient of variation (CoV, derived from SD of the difference between the measurements divided by mean value, expressed as %)

|  | **Larghat**[64] | **Morton**[65] | **Elkington**[8] | **Chih**[66] | **Likhite**[10] |
| --- | --- | --- | --- | --- | --- |
| **Index** | Fermi deconvolution and upslopes | Fermi deconvolution | Fermi deconvolution and upslopes | Upslopes | Fermi deconvolution |
| **N** | 11 | 16 | 16 | 20 | 10 |
| **Population** | Healthy volunteers | Healthy volunteers | Healthy volunteers (n=7), CAD (n=9) | CAD | Healthy volunteers |
| **Interstudy variability (CoV)** | MPR: 13-27% | MPR: 23.9% | MPR: 21%  MPRI: 41% | MPRI: 18.9% | MPR: 18.6% using a selfgated free breathing technique |
| **Interobserver-variability (CoV)** | MPR: 4-10% |  |  | MPRI: 9.0% |  |
| **Intraobserver-variability (CoV)** | MPR: 3-7% |  |  | MPRI: 5.3% |  |

**Normal values**

|  | **Index** | **N** | **Population** | **Age** | **Rest (ml/min/g)** | **Stress (ml/min/g)** | **MPR (I)** |
| --- | --- | --- | --- | --- | --- | --- | --- |
| **Morton [65]** | Fermi deconvolution | 11 | Healthy volunteers | 27+5 | 0.6±0.1 | 2.5±0.5 | 4.3±0.9 |
| **Bakir [67]** | Upslopes | 20 | Healthy women | 54±9 |  |  | 2.19±0.38 |
| **Larghat [64]** | Upslopes | 11 | Healthy volunteers | 33 ± 7 |  |  | Endocard: 1.54±0.3  Epicard: 1.81±0.35 |
|  | Fermi deconvolution |  |  |  |  |  | Endocard: 2.6±0.75  Epicard: 3.32±0.93 |

**Table 3d.3: Prospective outcome studies using stress CMR.** Follow-up is expressed in months. HRs are expressed as absolute values followed by 95% CI. All analysis are adjusted/multivariable unless otherwise indicated (†).CAD - coronary artery disease. MRP - magnetic resonance perfusion. DSMR - dobutamine stress magnetic resonance. MI - myocardial infarction. WMA - wall motion abnormalities. UA - unstable angina. LGE - late gadolinium enhancement. PCI - percutaneous coronary intervention. MPRI - myocardial perfusion reserve index. HF - heart failure. ACS – acute coronary syndrome. NICE - National Institute for Health and Care Excellence (UK).

|  | **N** | **Type** | **Population** | **Stress** | **Follow-up** | **Endpoint** | **CMR-outcomes** | | | |
| --- | --- | --- | --- | --- | --- | --- | --- | --- | --- | --- |
| **Lipinski[68]** | 11636 | Meta-analysis | Known or suspected CAD | MRP or DSMR | 32 | CV death,  MI, CV death/MI | MI | HR 7.7 (3.28-18.23) | < 0.0001 |  |
|  |  |  |  |  |  |  | CV death | HR 6.96 (4.13-11.74) | <0.0001 |  |
|  |  |  |  |  |  |  | CV death /MI | HR 6.5 (4.41-9.58) | <0.0001 |  |
|  |  |  |  |  |  |  | Event rate (CMR+ vs CMR -) | | | |
|  |  |  |  |  |  |  | CV death | 2.8±1.6 vs 0.3±0.3% | <0.0001 |  |
|  |  |  |  |  |  |  | MI | 2.6±2 vs 0.4±0.3% | <0.0005 |  |
|  |  |  |  |  |  |  | CV death/MI | 4.9±3.1 vs 0.8±0.7% | <0.0001 |  |
| **Jahnke[69]** | 513 | Observational  Prospective | Known or suspected CAD | MRP and DSMR | 27.6 | CV death /MI | 3-y event free survival | | | |
|  |  |  |  |  |  |  | CMR- | 99.2% | <0.001 |  |
|  |  |  |  |  |  |  | CMR + | 83.5% |  |  |
|  |  |  |  |  |  |  | MRP+ | HR 10.57 (2.86-39.1) | <0.001 |  |
|  |  |  |  |  |  |  | DSMR + | HR 4.72 (1.76-12.64) | 0.002 |  |
| **Bodi [70]** | 601 | Observational  Prospective | Known or suspected CAD | MRP + WMA | 18.4 | CV death /MI/UA | MRP+, WMA- | HR 2.2 (1.2-4.1) | 0.001 |  |
|  |  |  |  |  |  |  | MRP+, WMA+ | HR 3.8 (1.9-7.8) | 0.0002 |  |
|  |  |  |  |  |  |  | Effect of revascularization | | | |
|  |  |  |  |  |  |  | MRP+, WMA- | HR 1.1 (0.5-2.4) | 0.7 |  |
|  |  |  |  |  |  |  | MRP+, WMA+ | HR 0.2 (0.1-0.7) | 0.01 |  |
| **Hundley [71]** | 279 | Observational  Prospective | Known or suspected CAD | DSMR | 20 | CV death, MI | DSMR+ associated with death (HR 2.8, 1-7.5) and CV death/MI (HR 3.3, 1.1-9.7).  Increased risk of CV death/MI with more coronary territories (HR 4.5-7 with more than 1) or number of segments affected (HR 7.1 with 3-5 segments) | | | |
| **Kelle [72]** | 3138 | Observational  Prospective | Known or suspected CAD | DSMR | 40 | CV death /MI | DSMR + | HR 6.5 (4.6-9.3) | <0.001 |  |
|  |  |  |  |  |  |  | LGE | HR 2.2 (1.2-4.1) | <0.001 |  |
|  |  |  |  |  |  |  | Rest WMA | HR 1.6 (1.2-2.3) | <0.001 |  |
|  |  |  |  |  |  |  | Revascularization improved event-free survival only in those with DSMR+ | | | |
| **Kelle[73]** | 1369 | Observational  Prospective | Known or suspected CAD | DSMR | 44 | CV death /MI | 6-year event-free survival | | | |
|  |  |  |  |  |  |  | DSMR + | 92% | 0.001 |  |
|  |  |  |  |  |  |  |  | HR 2.99 (1.6-5.4) |  |  |
|  |  |  |  |  |  |  | DSMR- | 96.9% |  |  |
|  |  |  |  |  |  |  | No effect of revascularization if DSMR - (3.1% vs 3.2% events). In DSMR+ trend to higher events in medically treated (8% vs 5.4%, p=0.234) | | | |
| **Bodi[74]** | 420 | Observational  Prospective | Known or suspected CAD | MRP + WMA | 14 | CV death /MI/UA | MACE (abnormal vs normal) | | | |
|  |  |  |  |  |  |  | Rest WMA | 22 vs 5% | <0.0001 |  |
|  |  |  |  |  |  |  | WMA+ | 21 vs 4% | <0.0001 |  |
|  |  |  |  |  |  |  | MRP+ | 17 vs 5% | <0.0001 |  |
|  |  |  |  |  |  |  | LGE | 20 vs 6% | <0.0001 |  |
|  |  |  |  |  |  |  | Extent of stress WMA | HR 1.15 (1.06-1.24) per segment | 0.0006 |  |
| **Bingham[75]** | 908 | Observational  Prospective | Known or suspected CAD | MRP | 31 | CV death /MI /late revas- cularization | LVEF, aortic flow, LGE and abnormal perfusion predict adverse outcomes. Its combination enhances its prognostic value (increased χ² in Cox proportional model, 55.2, 63.3, 68 and 68.9, all p<0.00001).  Normal CMR 🡪 2.4% events/y (<1% CV death/MI) | | | |
| **Bodi [76]** | 1722 | Observational  Prospective | Known or suspected CAD | MRP + WMA | 10.2 | CV death /MI | CV death/MI (abnormal vs normal) | | | |
|  |  |  |  |  |  |  | Rest WMA | 6.8 vs 2% | <0.001 |  |
|  |  |  |  |  |  |  | WMA+ | 13.5 vs 2.3% | <0.001 |  |
|  |  |  |  |  |  |  | MRP+ | 6.2 vs 1.7% | <0.001 |  |
|  |  |  |  |  |  |  | LGE + | 6 vs 2.6% | 0.001 |  |
|  |  |  |  |  |  |  | Stress WMA is the only predictor of cardiac events | | | |
|  |  |  |  |  |  |  | WMA+ | HR 10.7 (5.22-21.98) | <0.0001 |  |
|  |  |  |  |  |  |  | WMA+ (per segment) | HR 1.17 (1.08-1.27) | 0.0001 |  |
| **Korosoglou [77]** | 1493 | Observational  Prospective | Known or suspected CAD | DSMR (WMA+MRP) | 24 | CV death /MI, late reavascularization | CV death/MI | | | |
|  |  |  |  |  |  |  | WMA+ | HR 5.9 (2.5-13.6) | <0.001 |  |
|  |  |  |  |  |  |  | MRP+ | HR 5.4(2.3-12.9) | <0.001 |  |
|  |  |  |  |  |  |  | Late revascularization | | | |
|  |  |  |  |  |  |  | WMA+ | HR 3.1 (1.7-5.6) | <0.001 |  |
|  |  |  |  |  |  |  | MRP+ | HR 6.2 (3.3-11.3) | <0.001 |  |
| **Buckert[78]** | 1229 | Observational  Prospective | Known or suspected CAD | MRP | 50 | CV death /MI/stroke | MRP+ | HR .21 (2-06-5) | <0.0001 |  |
|  |  |  |  |  |  |  | Event-free survival 🡪 95.6% vs 83.5% (MPR -/+) | | | |
| **Krittayaphong[31]** | 1232 | Observational  Prospective | Known or suspected CAD | MRP | 39 | CV death/ MI/UA/HF | CV death/MI | | | |
|  |  |  |  |  |  |  | MRP+ | HR 6.24 (2.7-14.44) | <0.001 |  |
|  |  |  |  |  |  |  | LGE+ | HR 3.64 (1.95-6.78) | <0.001† |  |
|  |  |  |  |  |  |  | CV death/MI/UA/HF | | | |
|  |  |  |  |  |  |  | MRP+ | HR 2.92 (1.86-4.6) | <0.001 |  |
|  |  |  |  |  |  |  | LGE+ | HR 3.76 (2.58-5.48) | <0.001† |  |
| **Greenwood**  **[79]** | 752 | Observational Prospective | Suspected CAD | MRP  SPECT | 60 | CV death, ACS, unscheduled revascularization or hospital admission for any CV cause | MRP+  SPECT+ | HR 2.77 (1.85-4.16)  HR 1.63 (1.11-2.39) | <0.0001  <0.013 |  |
| **Greenwood [80]** | 1202 | Multicenter, 3-parallel group, randomized clinical trial | Suspected CAD | MRP  SPECT  NICE | 12 | Protocol-defined unnecessary coronary angiography | CMR vs NICE | HR 0.21 (0.12-0.34) | <0.001 |  |
|  |  |  |  |  |  |  | CMR vs SPECT | HR 1.27 (0.79-2.03) | 0.32 |  |
|  |  |  |  |  |  | MACE | CMR vs NICE | 1.37(0.52-3.57) | 0.52 |  |
|  |  |  |  |  |  |  | CMR vs SPECT | 0.95(0.46-1.95) | 0.88 |  |

1. Klocke FJ, Simonetti OP, Judd RM, Kim RJ, Harris KR, Hedjbeli S, et al. Limits of Detection of Regional Differences in Vasodilated Flow in Viable Myocardium by First-Pass Magnetic Resonance Perfusion Imaging. Circulation. 2001;104:2412–6.

2. Lee DC. Magnetic Resonance Versus Radionuclide Pharmacological Stress Perfusion Imaging for Flow-Limiting Stenoses of Varying Severity. Circulation. 2004;110:58–65.

3. Christian TF, Aletras AH, Arai AE. Estimation of absolute myocardial blood flow during first-pass MR perfusion imaging using a dual-bolus injection technique: Comparison to single-bolus injection method. J. Magn. Reson. Imaging. 2008;27:1271–7.

4. Christian TF, Bell SP, Whitesell L, Jerosch-Herold M. Accuracy of Cardiac Magnetic Resonance of Absolute Myocardial Blood Flow With a High-Field System. JACC: Cardiovascular Imaging. 2009;2:1103–10.

5. Hsu L-Y, Groves DW, Aletras AH, Kellman P, Arai AE. A quantitative pixel-wise measurement of myocardial blood flow by contrast-enhanced first-pass CMR perfusion imaging: microsphere validation in dogs and feasibility study in humans. JACC Cardiovasc Imaging. 2012;5:154–66.

6. Schuster A, Zarinabad N, Ishida M, Sinclair M, van den Wijngaard JP, Morton G, et al. Quantitative assessment of magnetic resonance derived myocardial perfusion measurements using advanced techniques: microsphere validation in an explanted pig heart system. Journal of Cardiovascular Magnetic Resonance. 2014;16:1498.

7. Schuster A, Sinclair M, Zarinabad N, Ishida M, van den Wijngaard JPHM, Paul M, et al. A quantitative high resolution voxel-wise assessment of myocardial blood flow from contrast-enhanced first-pass magnetic resonance perfusion imaging: microsphere validation in a magnetic resonance compatible free beating explanted pig heart model. European Heart Journal - Cardiovascular Imaging. 2015;16:1082–92.

8. Schwitter J, Nanz D, Kneifel S, Bertschinger K, Büchi M, Knüsel PR, et al. Assessment of myocardial perfusion in coronary artery disease by magnetic resonance: a comparison with positron emission tomography and coronary angiography. Circulation. 2001;103:2230–5.

9. Ibrahim T, Nekolla SG, Schreiber K, Odaka K, Volz S, Mehilli J, et al. Assessment of coronary flow reserve: comparison between contrast-enhanced magnetic resonance imaging and positron emission tomography. JACC. 2002;39:864–70.

10. Pärkkä JP, Niemi P, Saraste A, Koskenvuo JW, Komu M, Oikonen V, et al. Comparison of MRI and positron emission tomography for measuring myocardial perfusion reserve in healthy humans. Magn. Reson. Med. 2006;55:772–9.

11. Fritz-Hansen T, Hove JD, Kofoed KF, Kelbaek H, Larsson HBW. Quantification of MRI measured myocardial perfusion reserve in healthy humans: A comparison with positron emission tomography. J. Magn. Reson. Imaging. 2008;27:818–24.

12. Pack NA, DiBella EV, Rust TC, Kadrmas DJ, McGann CJ, Butterfield R, et al. Estimating myocardial perfusion from dynamic contrast-enhanced CMR with a model-independent deconvolution method. Journal of Cardiovascular Magnetic Resonance. 2008;10:52.

13. Morton G, Chiribiri A, Ishida M, Hussain ST, Schuster A, Indermuehle A, et al. Quantification of Absolute Myocardial Perfusion in Patients With Coronary Artery Disease. Journal of the American College of Cardiology. 2012;60:1546–55.

14. Jogiya R, Morton G, De Silva K, Reyes E, Hachamovitch R, Kozerke S, et al. Ischemic Burden by 3-Dimensional Myocardial Perfusion Cardiovascular Magnetic Resonance: Comparison With Myocardial Perfusion Scintigraphy. Circ Cardiovasc Imaging [Internet]. 2014;7:647–54. Available from: http://circimaging.ahajournals.org/cgi/doi/10.1161/CIRCIMAGING.113.001620

15. Qayyum AA, Hasbak P, Larsson HBW, Christensen TE, Ghotbi AA, Mathiasen AB, et al. Quantification of myocardial perfusion using cardiac magnetic resonance imaging correlates significantly to rubidium-82 positron emission tomography in patients with severe coronary artery disease: a preliminary study. Eur J Radiol. 2014;83:1120–8.

16. al-Saadi N, Nagel E, Gross M, Bornstedt A, Schnackenburg B, Klein C, et al. Noninvasive detection of myocardial ischemia from perfusion reserve based on cardiovascular magnetic resonance. Circulation. 2000;101:1379–83.

17. al-Saadi N, Gross M, Paetsch I, Schnackenburg B, Bornstedt A, Fleck E, et al. Dobutamine induced myocardial perfusion reserve index with cardiovascular MR in patients with coronary artery disease. J Cardiovasc Magn Reson. 2002;4:471–80.

18. Nagel E, Klein C, Paetsch I, Hettwer S, Schnackenburg B, Wegscheider K, et al. Magnetic resonance perfusion measurements for the noninvasive detection of coronary artery disease. Circulation. 2003;108:432–7.

19. Bunce NH, Reyes E, Keegan J, Bunce C, Davies SW, Lorenz CH, et al. Combined coronary and perfusion cardiovascular magnetic resonance for the assessment of coronary artery stenosis. J Cardiovasc Magn Reson. 2004;6:527–39.

20. Cheng ASH, Pegg TJ, Karamitsos TD, Searle N, Jerosch-Herold M, Choudhury RP, et al. Cardiovascular magnetic resonance perfusion imaging at 3-tesla for the detection of coronary artery disease: a comparison with 1.5-tesla. J. Am. Coll. Cardiol. 2007;49:2440–9.

21. Cury RC, Cattani CAM, Gabure LAG, Racy DJ, de Gois JM, Siebert U, et al. Diagnostic performance of stress perfusion and delayed-enhancement MR imaging in patients with coronary artery disease. Radiology. 2006;240:39–45.

22. Wolff SD, Schwitter J, Coulden R, Friedrich MG. Myocardial first-pass perfusion magnetic resonance imaging a multicenter dose-ranging study. Circulation. 2004.

23. Giang TH, Nanz D, Coulden R, Friedrich M, Graves M, al-Saadi N, et al. Detection of coronary artery disease by magnetic resonance myocardial perfusion imaging with various contrast medium doses: first European multi-centre experience. Eur. Heart J. The Oxford University Press; 2004;25:1657–65.

24. Gebker R, Jahnke C, Paetsch I, Kelle S, Schnackenburg B, Fleck E, et al. Diagnostic performance of myocardial perfusion MR at 3 T in patients with coronary artery disease. Radiology. 2008;247:57–63.

25. Merkle N, Wöhrle J, Grebe O, Nusser T, Kunze M, Kestler HA, et al. Assessment of myocardial perfusion for detection of coronary artery stenoses by steady-state, free-precession magnetic resonance first-pass imaging. Heart. 2007;93:1381–5.

26. Kitagawa K, Sakuma H, Nagata M, Okuda S, Hirano M, Tanimoto A, et al. Diagnostic accuracy of stress myocardial perfusion MRI and late gadolinium-enhanced MRI for detecting flow-limiting coronary artery disease: a multicenter study. Eur Radiol. Springer-Verlag; 2008;18:2808–16.

27. Klein C, Gebker R, Kokocinski T, Dreysse S, Schnackenburg B, Fleck E, et al. Combined magnetic resonance coronary artery imaging, myocardial perfusion and late gadolinium enhancement in patients with suspected coronary artery disease. J Cardiovasc Magn Reson. 2008;10:45.

28. Klein C, Nagel E, Gebker R, Kelle S, Schnackenburg B, Graf K, et al. Magnetic resonance adenosine perfusion imaging in patients after coronary artery bypass graft surgery. JACC Cardiovasc Imaging. 2009;2:437–45.

29. Klem I, Heitner JF, Shah DJ, Sketch MH, Behar V, Weinsaft J, et al. Improved detection of coronary artery disease by stress perfusion cardiovascular magnetic resonance with the use of delayed enhancement infarction imaging. J. Am. Coll. Cardiol. 2006;47:1630–8.

30. Klem I, Greulich S, Heitner JF, Kim H, Vogelsberg H, Kispert E-M, et al. Value of cardiovascular magnetic resonance stress perfusion testing for the detection of coronary artery disease in women. JACC Cardiovasc Imaging. 2008;1:436–45.

31. Krittayaphong R, Boonyasirinant T, Saiviroonporn P, Nakyen S, Thanapiboonpol P, Yindeengam A, et al. Myocardial perfusion cardiac magnetic resonance for the diagnosis of coronary artery disease: do we need rest images? Int J Cardiovasc Imaging. Springer Netherlands; 2009;25 Suppl 1:139–48.

32. Klumpp BD, Seeger A, Doesch C, Doering J, Hoevelborn T, Kramer U, et al. High resolution myocardial magnetic resonance stress perfusion imaging at 3 T using a 1 M contrast agent. Eur Radiol. 2010;20:533–41.

33. Paetsch I, Jahnke C, Wahl A, Gebker R, Neuss M, Fleck E, et al. Comparison of dobutamine stress magnetic resonance, adenosine stress magnetic resonance, and adenosine stress magnetic resonance perfusion. Circulation. American Heart Association Journals; 2004;110:835–42.

34. Plein S, Radjenovic A, Ridgway JP, Barmby D, Greenwood JP, Ball SG, et al. Coronary artery disease: myocardial perfusion MR imaging with sensitivity encoding versus conventional angiography. Radiology. Radiological Society of North America; 2005;235:423–30.

35. Takase B, Nagata M, Kihara T, Kameyawa A, Noya K, Matsui T, et al. Whole-heart dipyridamole stress first-pass myocardial perfusion MRI for the detection of coronary artery disease. Jpn Heart J. 2004;45:475–86.

36. Thomas D, Strach K, Meyer C, Naehle CP, Schaare S, Wasmann S, et al. Combined myocardial stress perfusion imaging and myocardial stress tagging for detection of coronary artery disease at 3 Tesla. J Cardiovasc Magn Reson. BioMed Central; 2008;10:59.

37. Bernhardt P, Spiess J, Levenson B, Pilz G, Höfling B, Hombach V, et al. Combined Assessment of Myocardial Perfusion and Late Gadolinium Enhancement in Patients After Percutaneous Coronary Intervention or Bypass Grafts. JCMG. Elsevier Inc; 2009;2:1292–300.

38. Manka R, Jahnke C, Kozerke S, Vitanis V, Crelier G, Gebker R, et al. Dynamic 3-Dimensional Stress Cardiac Magnetic Resonance Perfusion Imaging. JAC. Elsevier Inc; 2011;57:437–44.

39. Meyer C, Strach K, Thomas D, Litt H, Nähle CP, Tiemann K, et al. High-resolution myocardial stress perfusion at 3 T in patients with suspected coronary artery disease. Eur Radiol. Springer-Verlag; 2008;18:226–33.

40. Greenwood JP, Maredia N, Younger JF, Brown JM, Nixon J, Everett CC, et al. Cardiovascular magnetic resonance and single-photon emission computed tomography for diagnosis of coronary heart disease (CE-MARC): a prospective trial. Lancet. 2012;379:453–60.

41. Greenwood JP, Motwani M, Maredia N, Brown JM, Everett CC, Nixon J, et al. Comparison of cardiovascular magnetic resonance and single-photon emission computed tomography in women with suspected coronary artery disease from the Clinical Evaluation of Magnetic Resonance Imaging in Coronary Heart Disease (CE-MARC) Trial. Circulation. 2014;129:1129–38.

42. Schwitter J, Wacker CM, Wilke N, al-Saadi N, Sauer E, Huettle K, et al. MR-IMPACT II: Magnetic Resonance Imaging for Myocardial Perfusion Assessment in Coronary artery disease Trial: perfusion-cardiac magnetic resonance vs. single-photon emission computed tomography for the detection of coronary artery disease: a comparative multicentre, multivendor trial. Eur. Heart J. The Oxford University Press; 2013;34:775–81.

43. Jaarsma C, Leiner T, Bekkers SC, Crijns HJ, Wildberger JE, Nagel E, et al. Diagnostic Performance of Noninvasive Myocardial Perfusion Imaging Using Single-Photon Emission Computed Tomography, Cardiac Magnetic Resonance, and Positron Emission Tomography Imaging for the Detection of Obstructive Coronary Artery Disease. Journal of the American College of Cardiology. 2012;59:1719–28.

44. Doyle M, Fuisz A, Kortright E, Biederman RW, Walsh EG, Martin ET, et al. The impact of myocardial flow reserve on the detection of coronary artery disease by perfusion imaging methods: an NHLBI WISE study. J Cardiovasc Magn Reson. 2003;5:475–85.

45. Gebker R, Jahnke C, Manka R, Hamdan A, Schnackenburg B, Fleck E, et al. Additional value of myocardial perfusion imaging during dobutamine stress magnetic resonance for the assessment of coronary artery disease. Circ Cardiovasc Imaging. 2008;1:122–30.

46. Pilz G, Bernhardt P, Klos M, Ali E, Wild M, Höfling B. Clinical implication of adenosine-stress cardiac magnetic resonance imaging as potential gatekeeper prior to invasive examination in patients with AHA/ACC class II indication for coronary angiography. Clin Res Cardiol. 2006;95:531–8.

47. Takx RAP, Blomberg BA, Aidi HE, Habets J, de Jong PA, Nagel E, et al. Diagnostic Accuracy of Stress Myocardial Perfusion Imaging Compared to Invasive Coronary Angiography With Fractional Flow Reserve Meta-Analysis. Circulation: Cardiovascular Imaging. 2014;8:e002666–6.

48. Watkins S, McGeoch R, Lyne J, Steedman T, Good R, McLaughlin M-J, et al. Validation of magnetic resonance myocardial perfusion imaging with fractional flow reserve for the detection of significant coronary heart disease. Circulation. American Heart Association, Inc; 2009;120:2207–13.

49. Manka R, Paetsch I, Kozerke S, Moccetti M, Hoffmann R, Schroeder J, et al. Whole-heart dynamic three-dimensional magnetic resonance perfusion imaging for the detection of coronary artery disease defined by fractional flow reserve: determination of volumetric myocardial ischaemic burden and coronary lesion location. European Heart Journal. 2012;33:2016–24.

50. Manka R, Wissmann L, Gebker R, Jogiya R, Motwani M, Frick M, et al. Multicenter evaluation of dynamic three-dimensional magnetic resonance myocardial perfusion imaging for the detection of coronary artery disease defined by fractional flow reserve. Circ Cardiovasc Imaging. Lippincott Williams & Wilkins; 2015;8:e003061–1.

51. Bettencourt N, Chiribiri A, Schuster A, Ferreira N, Sampaio F, Duarte R, et al. Cardiac magnetic resonance myocardial perfusion imaging for detection of functionally significant obstructive coronary artery disease: a prospective study. Int. J. Cardiol. 2013;168:765–73.

52. Ebersberger U, Makowski MR, Schoepf UJ, Platz U, Schmidtler F, Rose J, et al. Magnetic resonance myocardial perfusion imaging at 3.0 Tesla for the identification of myocardial ischaemia: comparison with coronary catheter angiography and fractional flow reserve measurements. European Heart Journal - Cardiovascular Imaging. 2013;14:1174–80.

53. Groothuis JGJ, Beek AM, Brinckman SL, Meijerink MR, van den Oever MLP, Hofman MBM, et al. Combined non-invasive functional and anatomical diagnostic work-up in clinical practice: the magnetic resonance and computed tomography in suspected coronary artery disease (MARCC) study. European Heart Journal. 2013;34:1990–8.

54. Layland J, Rauhalammi S, Watkins S, Ahmed N, McClure J, Lee MMY, et al. Assessment of Fractional Flow Reserve in Patients With Recent Non-ST-Segment-Elevation Myocardial Infarction: Comparative Study With 3-T Stress Perfusion Cardiac Magnetic Resonance Imaging. Circ Cardiovasc Interv. 2015;8:e002207.

55. Li M, Zhou T, Yang L-F, Peng Z-H, Ding J, Sun G. Diagnostic accuracy of myocardial magnetic resonance perfusion to diagnose ischemic stenosis with fractional flow reserve as reference: systematic review and meta-analysis. JACC Cardiovasc Imaging. 2014;7:1098–105.

56. Costa MA, Shoemaker S, Futamatsu H, Klassen C, Angiolillo DJ, Nguyen M, et al. Quantitative magnetic resonance perfusion imaging detects anatomic and physiologic coronary artery disease as measured by coronary angiography and fractional flow reserve. J. Am. Coll. Cardiol. 2007;50:514–22.

57. Huber A, Sourbron S, Klauss V, Schaefer J, Bauner KU, Schweyer M, et al. Magnetic resonance perfusion of the myocardium: semiquantitative and quantitative evaluation in comparison with coronary angiography and fractional flow reserve. Invest Radiol. 2012;47:332–8.

58. Jogiya R, Kozerke S, Morton G, De Silva K, Redwood S, Perera D, et al. Validation of Dynamic 3-Dimensional Whole Heart Magnetic Resonance Myocardial Perfusion Imaging Against Fractional Flow Reserve for the Detection of Significant Coronary Artery Disease. JAC. Elsevier Inc; 2012;60:756–65.

59. Pan J, Huang S, Lu Z, Li J, Wan Q, Zhang J, et al. Comparison of Myocardial Transmural Perfusion Gradient by Magnetic Resonance Imaging to Fractional Flow Reserve in Patients With Suspected Coronary Artery Disease. Am. J. Cardiol. 2015.

60. Bernhardt P, Walcher T, Rottbauer W, Wöhrle J. Quantification of myocardial perfusion reserve at 1.5 and 3.0 Tesla: a comparison to fractional flow reserve. Int J Cardiovasc Imaging. 2012;28:2049–56.

61. Kirschbaum SW, Springeling T, Rossi A, Duckers E, Gutiérrez-Chico JL, Regar E, et al. Comparison of adenosine magnetic resonance perfusion imaging with invasive coronary flow reserve and fractional flow reserve in patients with suspected coronary artery disease. Int. J. Cardiol. 2011;147:184–6.

62. Rieber J, Huber A, Erhard I, Mueller S, Schweyer M, Koenig A, et al. Cardiac magnetic resonance perfusion imaging for the functional assessment of coronary artery disease: a comparison with coronary angiography and fractional flow reserve. Eur. Heart J. 2006;27:1465–71.

63. Kühl HP, Katoh M, Buhr C, Krombach GA, Hoffmann R, Rassaf T, et al. Comparison of magnetic resonance perfusion imaging versus invasive fractional flow reserve for assessment of the hemodynamic significance of epicardial coronary artery stenosis. Am. J. Cardiol. 2007;99:1090–5.

64. Larghat AM, Maredia N, Biglands J, Greenwood JP, Ball SG, Jerosch-Herold M, et al. Reproducibility of first-pass cardiovascular magnetic resonance myocardial perfusion. J Magn Reson Imaging. 2013;37:865–74.

65. Morton G, Jogiya R, Plein S, Schuster A, Chiribiri A, Nagel E, et al. Quantitative cardiovascular magnetic resonance perfusion imaging: inter-study reproducibility. Eur Heart J Cardiovasc Imaging. 2012;13:954-60.

66. Chih S, Macdonald PS, Feneley MP, Law M, Graham RM, McCrohon JA. Reproducibility of adenosine stress cardiovascular magnetic resonance in multi-vessel symptomatic coronary artery disease. J Cardiovasc Magn Reson. BioMed Central; 2010;12:42.

67. Bakir M, Wei J, Nelson MD, Mehta PK, Haftbaradaran A, Jones E, et al. Cardiac magnetic resonance imaging for myocardial perfusion and diastolic function-reference control values for women. Cardiovasc Diagn Ther. 2016;6:78-86.

68. Lipinski MJ, McVey CM, Berger JS, Kramer CM, Salerno M. Prognostic value of stress cardiac magnetic resonance imaging in patients with known or suspected coronary artery disease: a systematic review and meta-analysis. J. Am. Coll. Cardiol. 2013;62:826–38.

69. Jahnke C, Nagel E, Gebker R, Kokocinski T, Kelle S, Manka R, et al. Prognostic value of cardiac magnetic resonance stress tests: adenosine stress perfusion and dobutamine stress wall motion imaging. Circulation. 2007;115:1769–76.

70. Bodi V, Sanchis J, Lopez-Lereu MP, Nunez J, Mainar L, Monmeneu JV, et al. Prognostic and therapeutic implications of dipyridamole stress cardiovascular magnetic resonance on the basis of the ischaemic cascade. Heart. 2009;95:49–55.

71. Hundley WG, Morgan TM, Neagle CM, Hamilton CA, Rerkpattanapipat P, Link KM. Magnetic resonance imaging determination of cardiac prognosis. Circulation. 2002;106:2328–33.

72. Kelle S, Nagel E, Voss A, Hofmann N, Gitsioudis G, Buss SJ, et al. A bi-center cardiovascular magnetic resonance prognosis study focusing on dobutamine wall motion and late gadolinium enhancement in 3,138 consecutive patients. J. Am. Coll. Cardiol. 2013;61:2310–2.

73. Kelle S, Chiribiri A, Vierecke J, Egnell C, Hamdan A, Jahnke C, et al. Long-term prognostic value of dobutamine stress CMR. JACC Cardiovasc Imaging. 2011;4:161–72.

74. Bodi V, Sanchis J, Lopez-Lereu MP, Nunez J, Mainar L, Monmeneu JV, et al. Prognostic value of dipyridamole stress cardiovascular magnetic resonance imaging in patients with known or suspected coronary artery disease. J. Am. Coll. Cardiol. 2007;50:1174–9.

75. Bingham SE, Hachamovitch R. Incremental prognostic significance of combined cardiac magnetic resonance imaging, adenosine stress perfusion, delayed enhancement, and left ventricular function over preimaging information for the prediction of adverse events. Circulation. 2011;123:1509–18.

76. Bodi V, Husser O, Sanchis J, Nunez J, Monmeneu JV, Lopez-Lereu MP, et al. Prognostic implications of dipyridamole cardiac MR imaging: a prospective multicenter registry. Radiology. 2012;262:91–100.

77. Korosoglou G, Elhmidi Y, Steen H, Schellberg D, Riedle N, Ahrens J, et al. Prognostic value of high-dose dobutamine stress magnetic resonance imaging in 1,493 consecutive patients: assessment of myocardial wall motion and perfusion. J. Am. Coll. Cardiol. 2010;56:1225–34.

78. Buckert D, Dewes P, Walcher T, Rottbauer W, Bernhardt P. Intermediate-term prognostic value of reversible perfusion deficit diagnosed by adenosine CMR: a prospective follow-up study in a consecutive patient population. JACC Cardiovasc Imaging. 2013;6:56–63.

79. Greenwood JP, Maredia N, Younger JF, Brown JM, Nixon J, Everett CC, et al. Cardiovascular magnetic resonance and single-photon emission computed tomography for diagnosis of coronary heart disease (CE-MARC): a prospective trial. The Lancet. 2012;379:453–60.

80. Greenwood JP, Ripley DP, Berry C, McCann GP, Plein S, Bucciarelli-Ducci C, et al. Effect of Care Guided by Cardiovascular Magnetic Resonance, Myocardial Perfusion Scintigraphy, or NICE Guidelines on Subsequent Unnecessary Angiography Rates: The CE-MARC 2 Randomized Clinical Trial. JAMA. 2016;316:1051-60.
