# Supplementary material for: Society for Cardiovascular Magnetic Resonance (SCMR) expert consensus for CMR imaging endpoints in clinical research: part I - analytical validation and clinical qualification
Source: J Cardiovasc Magn Reson. 2018 Sep 20;20:67. doi: 10.1186/s12968-018-0484-5 (PMC6147157; doi:10.1186/s12968-018-0484-5)
Supplement: Supplementary file 8 — Vascular CMR measurements. Table 3e.1: Comparative (validation) studies for CMR vascular endpoints with alternative techniques. Table 3e.2: Reproducibility of vascular endpoints. Table 3: Normal values for aortic dimensions (Table A), PWV and aortic distensibility (Table B) with CMR according to age and sex. Table 4: Outcome studies with PWV with CMR confirming the predictive associations. (DOCX 49 kb) [file 12968_2018_484_MOESM8_ESM.docx]

## Vascular CMR measurements

**Table 3e.1: Comparative (validation) studies for CMR vascular endpoints with alternative techniques.** Agreement is expressed as Pearson´s correlation coefficient (R), coefficient of variation (CoV, derived from SD of the difference between the measurements divided by mean value, expressed as %) or linear regression index (R^2^). PWV – pulse wave velocity (expressed as m/s). CAD - coronary artery disease. Ao - aorta. T-p - through-plane velocity-encoding CMR. I-p - in-plane velocity-encoding CMR. BA-PWV - brachial-ankle pulse wave velocity. DST - distensibility. AA - ascending aorta. DA - descending aorta. C-F PWV - carotid-femoral pulse wave velocity. TOF – time of flight angiography

| **Study** | **N** | **Population** | **Reference** | **Parameter** | **PWV(invasive) vs. CMR** | | |
| --- | --- | --- | --- | --- | --- | --- | --- |
|  |  |  |  |  | **Agreement** | |  |
| Grotenhuis [1] | 18 | Suspected CAD | Invasive PWV | PWV (total, proximal, distal) | Ao_total_ | 6.5±1.1 vs 6.2±1.1 | 0.08 |
|  |  |  |  |  |  | R=0.53 |  |
|  |  |  |  |  | Ao_prox_ | 6.5±1.3 vs 6.2±1.1 | 0.16 |
|  |  |  |  |  |  | R=0.69 |  |
|  |  |  |  |  | Ao_distal_ | 6.9±1.1 vs 6.1±1.0 | <0.01 |
|  |  |  |  |  |  | R=0.71 |  |
| Westenberg[2] | 14 | Suspected CAD | Invasive PWV | PWV Ao_total_ | T-p | R=0.58 | 0.03 |
|  |  |  |  |  |  | CoV 10% |  |
|  |  |  |  |  | I-p | R=0.75 | 0.003 |
|  |  |  |  |  |  | CoV 12% |  |
| Westenberg [3] | 17 | Suspected CAD | Invasive PWV | PWV (proximal and distal) | I-p Ao _prox_ | R=0.69 | 0.002 |
|  |  |  |  |  |  | CoV 24% |  |
|  |  |  |  |  | I-p Ao _distal_ | PCC=0.91 | <0.001 |
|  |  |  |  |  |  | CoV 19% |  |
| Kim [4] | 124 | Healthy volunteers | BA-PWV | PWV (total, proximal, distal, abdominal)  DST (AA, DAproximal, DAdistal, abdominal) | BA-PWV yields higher values (45% average) | | |
|  |  |  |  |  | PWV Ao_total_ | R^2^=0.697 | <0.001 |
|  |  |  |  |  | PWV Ao_prox_ | R^2^=0.588 | <0.001 |
|  |  |  |  |  | PWV Ao_distal_ | R^2^=0.468 | <0.001 |
|  |  |  |  |  | PWV Ao_abd_ | R^2^=0.418 | <0.001 |
|  |  |  |  |  | DST AA | R^2^=0.673 | <0.001 |
|  |  |  |  |  | DST DA_prox_ | R^2^=0.626 | <0.001 |
|  |  |  |  |  | DST DA_distal_ | R^2^=0.596 | <0.001 |
|  |  |  |  |  | DST _Abd_ | R^2^=0.583 | <0.001 |
| Nelson [5] | 20 | Healthy volunteers | Aplanation tonometry  (C-F PWV) | DST (AA, DAproximal, DAdistal) | DST AA | R^2^=0.57 |  |
|  |  |  |  |  | DST DA_prox_ | R^2^=0.60 |  |
|  |  |  |  |  | DST DA_distal_ | R^2^=0.72 |  |
| [Biasiolli](https://www.ncbi.nlm.nih.gov/pubmed/?term=Biasiolli%20L%5BAuthor%5D&cauthor=true&cauthor_uid=23953780) [6] | 18 | Patients with significant carotid artery stenosis | TOF | Arterial Wall measurement |  | Cohen's κ = 0.68 |  |
| Chai [7] | 26 | Patients with significant carotid artery stenosis | Histology | T2 value (T2 mapping) | Plaque type (AHA classification) | R^2^=0.808  (Cohen’s κ = 0.73) |  |
|  |  |  |  |  | Lipid content (area) | R^2^=0.85 | <0.001 |

**Table 3e.2. Reproducibility of vascular endpoints.** Reproducibility is presented as coefficient of variation (CoV, derived from SD of the difference between the measurements divided by mean value, expressed as %), Pearson´s R§ or mean difference±SD*. PWV: pulse wave velocity. DST: distensibility. Ao: aorta. T-p: through-plane velocity- encoding CMR. I-p: In- plane velocity-encoding CMR.

| **Anatomical measures** | **Chan**§[8] | **Roes**[9] | | |
| --- | --- | --- | --- | --- |
|  | Patients (=16) | Healthy volunteers (n=10) | | |
| Imaging technique | T2W-TSE | HASTE (T2-3D BB) | | |
| Thoracic descending aorta | Interstudy | Intraobserver | Interobserver | Interstudy |
| Lumen area | 0.99 |  |  |  |
| Mean vessel wall volume | 0.85 | 2.3% | 3.5% | 0.945 5.8% |

| **Functional measurements** | **Westenberg**[2] | | | | | | **Grotenhuis**[1] | | | **Noda***[10] | **Nelson**[5] |
| --- | --- | --- | --- | --- | --- | --- | --- | --- | --- | --- | --- |
|  | **PWV** | | | | | | | | | | **DST** |
| Measurement | Total Ao | | Proximal Ao | | Distal Ao | | Total Ao | Proximal Ao | Distal Ao | Proximal Ao |  |
| Method | T-p | I-p | T-p | I-p | T-p | I-p | T-p | T-p | T-p | T-p | T-p |
| Intraobserver | 3% | 3% | 6% | 4% | 2% | 3% |  |  |  | 0.1±0.6 | 1% |
| Interobserver | 3% | 4% | 11% | 8% | 5% | 5% |  |  |  | 0.1±1.4 | 2% |
| Interstudy | 13% | 7% | 17% | 10% | 16% | 12% | 9% | 13% | 9% | -0.05±3.0 |  |

**Table 3: Normal values for aortic dimensions (Table A), PWV and aortic distensibility (Table B) with CMR according to age and sex.** Dimensions expressed in mm (mean value followed by 95% CI in brackets, or mean value±SD); PWV m/s and distensibility as kPa ^−1^ x 10 ^−3^ or §mmHg-^1^. PWV – pulse wave velocity. DST - distensibility. AA - ascending aorta. DA - descending aorta. AbdA – abdominal aortaBSA – body surface area.

| **Table A** | **Sex** | **Age (years)** | | | | | | |  | |  | | |  | |
| --- | --- | --- | --- | --- | --- | --- | --- | --- | --- | --- | --- | --- | --- | --- | --- |
| **Turkbey** [11] | **AA** | **45-54** | | **55-64** | | **65-74** | | | **75-84** | |  | | |  | |
|  | Male | 31.6(27.2-37.3) | | 32.8(28.2-40.7) | | 34.2(28.1-40.7) | | | 34.7(28.6-40.8) | |  | | |  | |
|  | BSA indexed (mm/m^2^) | 15.9 (13.3-19.5) | | 16.8 (13.6-21.1) | | 17.8 (14.2-21.8) | | | 18.6 (15.2-22.6) | |  | | |  | |
|  | Female | 28.8(24.6-34.4) | | 30.1 (25.7-36.4) | | 30.6 (26.1-36.3) | | | 31.1 (26.8-37.1) | |  | | |  | |
|  | BSA indexed (mm/m^2^) | 16.7 (13.5-20.7) | | 17.6 (14.8-22.1) | | 8.1 (14.5-22.1) | | | 9.7 (15.3-28.2) | |  | | |  | |
| **Burman** [12] | **Aortic root cusp-commissure** | **20-29** | | **30-39** | | **40-49** | | | **50-59** | | **60-69** | | | **70-79** | |
|  |  | **Systole** | **Diastole** | **Systole** | **Diastole** | **Systole** | **Diastole** | **Systole** | | **Diastole** | **Systole** | **Diastole** | **Systole** | | **Diastole** |
|  | Male | 34.4(26–43) | 32.8(25-40) | 33.8 (26–41) | 32.0(24-40) | 36.0 (31–41) | 34.1(30-40) | 36.3(25-48) | | 35.2(24-47) | 37.4(32-43) | 36.2(31-41) | 37.8(28-44) | | 37.9)30-44) |
|  | BSA indexed (mm/m^2^) | 17.7 (14–22) | 16.9(13–21) | 17.2 (13–21) | 16.2(12-20) | 17.4 (15–20) | 16.5 (14-19) | 18.5 (14–23) | | 17.9 (13-23) | 19.2 (15–24) | 18.6 (15-23) | 19.4 (17–22) | | 19.0 (16–22) |
|  | Female | 30.2 (21-40) | 28.4(19-38) | 30.0(24-36) | 28.7(23-35) | 33.9(29-39) | 32.8(28-38) | 31.4(26-36) | | 30.6(25-36) | 32.8(28-37) | 32.0(28-36) | 32.9(30-36) | | 32.0(29-35) |
|  | BSA indexed (mm/m^2^) | 17.6(13-22) | 16.6(12-21) | 18.4(16-21) | 17.6(15-20) | 19.0(14-24) | 18.4(13-23) | 18.9(16-22) | | 18.5(15-22) | 19.0(14-24) | 18.6(15-22) | 19.8(18-22) | | 19.3(17-21) |
| **Redheuil** [13] | **AA** | **46±16** | **<30** | **>70** |  |  |  |  | |  |  |  |  | |  |
|  | Male | 31±4 | 27.5±2.8 | 33.2±4.3 |  |  |  |  | |  |  |  |  | |  |
|  | Female | 30±4 |  |  |  |  |  |  | |  |  |  |  | |  |
|  | **DA** |  |  |  |  |  |  |  | |  |  |  |  | |  |
|  | Male | 24±3 | 20.3±1.6 | 24.3±2.7 |  |  |  |  | |  |  |  |  | |  |
|  | Female | 22±3 |  |  |  |  |  |  | |  |  |  |  | |  |
|  | **AbdA** |  |  |  |  |  |  |  | |  |  |  |  | |  |
|  | Male | 21±3 | 18.3±1.4 | 21.5±13.3 |  |  |  |  | |  |  |  |  | |  |
|  | Female | 20±2 |  |  |  |  |  |  | |  |  |  |  | |  |

| **Table B** | Numbers of participants (male) | **Biomarker** | **Age** | | | | | |
| --- | --- | --- | --- | --- | --- | --- | --- | --- |
|  |  |  | **20-29** | **30-39** | **40-49** | **50-59** | **60-69** | **≥70** |
| **Redheuil** [14] | 122 (60) | Central PWV | 3.5±0.5 | 3.9±1.1 | 5.6±1.4 | 7.2±2.3 | 9.7±2.9 | 11.1±4.6 |
|  |  | AA DST | 74±23 | 61±23 | 31±18 | 18±7 | 12±7 | 10±6 |
|  |  | DA DST | 72±18 | 70±24 | 38±17 | 29±13 | 18±8 | 17±6 |
|  |  |  | **Sex** | | | | | |
|  |  |  | **Men** | | | **Women** | | |
| **Rose**[15] | 26 (13) | AA DST | 6.1±2.5 | | | 8.6±2.7 | | |
|  |  | DA DST | 5.1±2.4 | | | 7.2±1.6 | | |
| **Redheuil** [13] | 100 (45) | AA DST | 3.8±2.7 | | | 3.8±3.1 | | |
|  |  | DA DST | 4.4±3.0 | | | 4.5±2.4 | | |
|  |  | AbdA DST | 8.8±3.8 | | | 7.3±4.7 | | |
| **Voges** [16] | 71 (30) | Central PWV | 3.7 ± 0.9 | | | 3.5 ± 0.6 | | |
|  |  | AA DST | 8.5 ± 4.2 | | | 9.2 ± 3.0 | | |
|  |  | DA DST | 7.7 ± 2.7 | | | 8.8 ± 3.1 | | |

**Table 4: Outcome studies with PWV with CMR confirming the predictive associations.**

Dimensions expressed in mm; PWV m/s and distensibility as kPa ^−1^ x 10 ^−3^ or §mmHg-^1^. DST - distensibility. AA - ascending aorta. PWV – pulse wave velocity. * adjusted for age, sex, weight, height, ethnicity, mean BP, smoking, antihypertensive medication, total cholesterol, HDL cholesterol, and diabetes. § adjusted for age, sex, ethnicity, systolic blood pressure, use of blood pressure medication, resting heart rate, diabetes mellitus, current smoking, body mass index, and hypercholesterolemia.

|  | **Study type** | **Population** | **Age, number of participants (male, %)** | **Follow up (years)** | **Endpoint** | **Outcome (event rate)** | **Statistics (HR (95%CI)** |
| --- | --- | --- | --- | --- | --- | --- | --- |
| **Redheuil** [17] | Observational prospective | Asymptomatic participants | 61±10  3675 (56) | 8.5 | AA DST | All-cause mortality (9.3%) | Univariate:  6.5 (2.4-12.3), p<0.001 (for upper quintile) |
|  |  |  |  |  |  |  | Multivariate*:  2.3 (1.2-4.4), p=0.009 |
|  |  |  |  |  |  | CV mortality (6.7%) | Univariate:  5.7 (2.8-11.7), p<0.001 (for upper quintile) |
|  |  |  |  |  |  |  | Multivariate*:  1.9 (0.9-3.8), p=0.09 |
| **Maroules** [18] | Observational prospective | Asymptomatic participants | 44±10  2122 (44) | 7.8±1.5 | AA DST | CV mortality (6.9%) | Univariate:  1.63 (1.50-1.77), p<0.001 |
|  |  |  |  |  |  |  | Multivariate§:  1.18(0.95-1.46), p=0.08 |
|  |  |  |  |  | PWV |  | Univariate:  1.25 (1.11-1.41), p<0.001 |
|  |  |  |  |  |  |  | Multivariate§:  1.11(0.89-1.32), p=0.28 |

References

1. Grotenhuis HB, Westenberg JJM, Steendijk P, van der Geest RJ, Ottenkamp J, Bax JJ, et al. Validation and reproducibility of aortic pulse wave velocity as assessed with velocity-encoded MRI. J Magn Reson Imaging. 2009; 30:521–6.

2. Westenberg JJM, de Roos A, Grotenhuis HB, Steendijk P, Hendriksen D, van den Boogaard PJ, et al. Improved aortic pulse wave velocity assessment from multislice two-directional in-plane velocity-encoded magnetic resonance imaging. J Magn Reson Imaging. 2010;32:1086–94.

3. Westenberg JJ, van Poelgeest EP, Steendijk P, Grotenhuis HB, Jukema JW, de Roos A. Bramwell-Hill modeling for local aortic pulse wave velocity estimation: a validation study with velocity-encoded cardiovascular magnetic resonance and invasive pressure assessment. J Cardiovasc Magn Reson. 2012;14:2.

4. Kim EK, Chang S-A, Jang SY, Choi KH, Huh EH, Kim JH, et al. Brachial-ankle pulse wave velocity as a screen for arterial stiffness: a comparison with cardiac magnetic resonance. Yonsei Med J. 2015;56:617–24.

5. Nelson AJ, Worthley SG, Cameron JD, Willoughby SR, Piantadosi C, Carbone A, et al. Cardiovascular magnetic resonance-derived aortic distensibility: validation and observed regional differences in the elderly. J Hypertens. 2009;27:535–42.

6. Biasiolli L, Lindsay AC, Chai JT, Choudhury RP, Robson MD. In-vivo quantitative T2 mapping of carotid arteries in atherosclerotic patients: segmentation and T2 measurement of plaque components. J Cardiovasc Magn Reson. 2013;15:69.

7. Chai JT, Biasiolli L, Li L, Alkhalil M, Galassi F, Darby C, et al. Quantification of Lipid-Rich Core in Carotid Atherosclerosis Using Magnetic Resonance T2 Mapping: Relation to Clinical Presentation. JACC: Cardiovascular Imaging. 2017;10:747–56.

8. Chan SK, Jaffer FA, Botnar RM, Kissinger KV, Goepfert L, Chuang ML, et al. Scan reproducibility of magnetic resonance imaging assessment of aortic atherosclerosis burden. J Cardiovasc Magn Reson. 2001;3:331–8.

9. Roes SD, Westenberg JJM, Doornbos J, van der Geest RJ, Angelié E, de Roos A, et al. Aortic vessel wall magnetic resonance imaging at 3.0 Tesla: a reproducibility study of respiratory navigator gated free-breathing 3D black blood magnetic resonance imaging. Magn Reson Med. 2009;61:35–44.

10. Noda C, Ambale-Venkatesh B, Ohyama Y, Liu C-Y, Chamera E, Redheuil A, et al. Reproducibility of functional aortic analysis using magnetic resonance imaging: the MESA. Eur Heart J Cardiovasc Imaging. 2016;17:909–17.

11. Turkbey EB, Jain A, Johnson C, Redheuil A, Arai AE, Gomes AS, et al. Determinants and normal values of ascending aortic diameter by age, gender, and race/ethnicity in the Multi-Ethnic Study of Atherosclerosis (MESA). J Magn Reson Imaging. 2014;39(2):360–8.

12. Burman ED, Keegan J, Kilner PJ. Aortic root measurement by cardiovascular magnetic resonance: specification of planes and lines of measurement and corresponding normal values. Circulation: Cardiovascular Imaging. 2008;1(2):104–13.

13. Redheuil A, Yu W-C, Mousseaux E, Harouni AA, Kachenoura N, Wu CO, et al. Age-related changes in aortic arch geometry: relationship with proximal aortic function and left ventricular mass and remodeling. J Am Coll Cardiol. 2011;58:1262–70.

14. Redheuil A, Yu W-C, Wu CO, Mousseaux E, de Cesare A, Yan R, et al. Reduced ascending aortic strain and distensibility: earliest manifestations of vascular aging in humans. Hypertension. 2010;55(2):319–26.

15. Rose J-L, Lalande A, Bouchot O, Bourennane E-B, Walker PM, Ugolini P, et al. Influence of age and sex on aortic distensibility assessed by MRI in healthy subjects. Magnetic Resonance Imaging.2010;28(2):255–63.

16. Voges I, Jerosch-Herold M, Hedderich J, Pardun E, Hart C, Gabbert DD, et al. Normal values of aortic dimensions, distensibility, and pulse wave velocity in children and young adults: a cross-sectional study. J Cardiovasc Magn Reson. 2012;14:77.

17. Redheuil A, Wu CO, Kachenoura N, Ohyama Y, Yan RT, Bertoni AG, et al. Proximal aortic distensibility is an independent predictor of all-cause mortality and incident CV events: the MESA study. J Am Coll Cardiol. 2014;64:2619–29.

18. Maroules CD, Khera A, Ayers C, Goel A, Peshock RM, Abbara S, et al. Cardiovascular outcome associations among cardiovascular magnetic resonance measures of arterial stiffness: the Dallas heart study. Journal of Cardiovascular Magnetic Resonance. 2014;16:33.

.
